# Identification of hub necroptosis-related lncRNAs for prognosis prediction of esophageal carcinoma

**DOI:** 10.18632/aging.204763

**Published:** 2023-06-01

**Authors:** Zhengdong Luo, E Ding, Longchen Yu, Wenwu Wang, Qining Guo, Xinyang Li, Yifeng Wang, Tingting Li, Yi Zhang, Xin Zhang

**Affiliations:** 1 Department of Clinical Laboratory, Qilu Hospital of Shandong University, Jinan, Shandong Province, China; 2 Hangzhou Lin’an District Fourth People’s Hospital, Hangzhou, Zhejiang Province, China

**Keywords:** esophageal carcinoma, necroptosis, long non-coding RNA, prognosis, LncRNA PVT1

## Abstract

Necroptosis is a newly identified programmed cell death associated with the biological process of various cancers, including esophageal carcinoma (ESCA). Meanwhile, the dysregulation of long non-coding RNAs (lncRNAs) is greatly implicated in ESCA progression and necroptosis regulation. However, the lncRNAs involved in regulating necroptosis in ESCA are still unclear. In this study, we aim to explore the expression profile of necroptosis-related lncRNAs (NRLs), and evaluate their roles in ESCA prognosis and treatment. In the present study, 198 differentially expressed NRLs were identified between the ESCA and adjacent normal tissues through screening the data extracted from the Cancer Genome Atlas (TCGA) database. And, a prognostic panel consisting of 6 NRLs was constructed using the LASSO algorithm and multivariate Cox regression analysis. The ESCA patients with high risks had a markedly reduced survival time and higher mortality prevalence. Moreover, C-index of 6 NRLs-panel was superior to 48 published prognostic models based on lncRNAs or mRNAs for ESCA. There were significant differences between the high-risk and low-risk groups in tumor-related pathways, genetic mutations, and drug sensitivity responses. *In vitro* analysis revealed that inhibition of PVT1 impeded the proliferation, migration, and colony formation of ESCA cells, increased the expressions of p-RIP1 and p-MLKL and promoted necroptosis. By contrast, PVT1 overexpression resulted in a decrease in necroptotic cell death events, thus promoting tumor progression. Collectively, the established 6-NRLs panel was a promising biomarker for the prognostic prediction of ESCA. Moreover, our current findings provided potential targets for individualized therapy for ESCA patients.

## INTRODUCTION

As one of the most common gastrointestinal tumors worldwide, esophageal carcinoma (ESCA) is often associated with delayed diagnosis and inferior outcomes [1, 2]. At present, surgery remains the only curative measure for treating early ESCA, while it has little clinical success for patients with advanced cancer [3]. Despite the effective improvement in early detection and combination therapy regimens, the 5-year survival rate of ESCA patients is still as low as 20% [4, 5]. Although some combined therapeutic regimens have ameliorated the survival time of patients with advanced diseases [6, 7], they are still controversial in clinical responses. The effective prediction of the prognosis is important for appropriate treatment planning. Therefore, it is urgently necessary to identify novel biomarkers to predict the prognosis of ESCA.

As a newly identified programmed cell death, necroptosis is regulated by TNF-α and RIPK1/RIPK3-dependent phosphorylation of MLKL [8, 9], and such a process is different from apoptosis, necrosis, pyroptosis, ferroptosis, and so on [10]. Accumulating evidence has shown that necroptosis is widely associated with tumorigenesis and the progression of malignancies [11, 12]. It has been reported that immunogenic necroptotic cells can be adopted as vaccines to induce efficient antitumor immunity to eliminate tumor cells [13]. Furthermore, Zheng et al. [14] have revealed that STAT3β is up-regulated in the cytoplasm of esophageal squamous cell carcinoma (ESCC), leading to enhanced sensitivity to concurrent chemoradiotherapy (CCRT) via inducing necroptosis. Importantly, studies have found that necroptosis is closely related to tumor-infiltrating lymphocytes, which can be adopted as an independent prognostic parameter of ESCC [15]. Collectively, necroptosis-related regulators may be potential prognostic biomarkers for ESCA.

Long non-coding RNAs (lncRNAs) are a group of special non-coding RNAs with transcript lengths over 200 nucleotides [16], which are generally involved in the regulation of protein-encoding genes through epigenetic modulations, transcriptional regulation, and post-transcriptional regulation [17, 18]. Increasing studies have implicated that lncRNAs contribute to the development of ESCA [19]. Studies have found that LINC00680, a competing endogenous RNA (CeRNA), can sponge microRNA-423-5p to induce the expression of PAK6 and promote the progression of ESCC [20]. Furthermore, lncRNAs VESTAR and CASC9 are overexpressed in ESCC and promote cancer metastasis through other mechanisms, including transcriptional regulation and epigenetic modification [21, 22]. At present, many reports have revealed that the necroptosis-related lncRNAs (NRLs) may be used as prognostic biomarkers for some cancers [23–25]. However, their potential prognostic value in ESCA remains largely unexplored.

LncRNA plasmacytoma variant translocation 1(PVT1) is located on chromosome 8 of the c-Myc gene and is abnormally expressed in multiple malignancies, including nasopharyngeal carcinoma [26], colorectal cancer [27], ovarian cancer [28], and so on. PVT1 usually interacts with c-Myc to mediate malignant tumor progression, such as proliferation, invasion, metastasis, therapeutic resistance, etc. In general, PVT1 overexpression is often associated with poor prognosis in patients, and it has been regarded as a novel carcinogenic factor [29]. Studies discovered that PVT1 is highly expressed in renal clear cell carcinoma tissues and with poor prognosis in patients. Functional experiments demonstrated that PVT1 could stabilize the expression of HIF2α by regulating the ubiquitination-dependent degradation pathway, thereby promoting the invasion and metastasis of cancer cells [30]. Furthermore, PVT1 can act as a sponge to competitively bind miR-128-3p and elevate FOXQ1, inducing epithelial-mesenchymal transformation of cancer cells [31]. In addition, HAT1 promoted the expression of PVT1 by promoting the binding of BRD4 to the PVT1 promoter, thereby mediating gemcitabine resistance [32]. Intriguingly, PVT1 may also serve as a good prognostic indicator for the early stages of some cancers, such as ovarian carcinoma [33]. Nevertheless, the detailed mechanism by which PVT1 regulated esophageal carcinoma progression remains poorly understood and thus needs to be further elucidated.

In the present work, we thoroughly explored the expression pattern of NRLs in ESCA and established a prognostic panel based on six NRLs by least absolute shrinkage and selection operator (LASSO) and multi-Cox regression. Moreover, we assessed its predictive value using principal component analysis (PCA), time-dependent receiver operating characteristic (ROC), concordance index (C-index), nomogram, tumor mutational burden (TMB) analysis, and chemotherapy response analysis of ESCA patients. Besides, we further performed the gene set enrichment analysis (GSEA) to explore its underlying mechanisms. Finally, the expression of one NRL, PVT1, in ESCA cells was detected, suggesting its role in necroptosis. Collectively, these data provided valuable insights into the progression and prognosis of ESCA.

## MATERIALS AND METHODS

### Information acquirement and data manipulation

A total of 162 ESCA tissue samples and 11 adjacent non-tumor tissue samples were acquired from The Cancer Genome Atlas (TCGA, https://portal.gdc.cancer.gov/), and their corresponding transcriptomic FPKM data, somatic mutations, and clinical information were also collected. Patients with missing information on survival time or those with survival time < 1 month were excluded from the analysis. In addition, 159 necroptosis-related genes (NRGs) were acquired from the Kyoto Encyclopedia of Genes and Genomes (KEGG, https://www.kegg.jp/) (Supplementary Table 1).

### Enrichment and interaction analysis of differentially expressed NRGs (DE-NRGs)

The DE-NRGs between ESCA and adjacent normal tissues were identified using the “limma” package. Subsequently, Metascape (https://metascape.org/gp/index.html#/) was adopted to conduct the functional enrichment analysis and protein-protein interaction (PPI) network of DE-NRGs. Besides, NRG mutations were also assessed in ESCA using the cBioPortal website (http://www.cbioportal.org/).

### Identification of prognostic differentially expressed NRLs (DE-NRLs)

To identify the lncRNA co-expressed with NRGs in ESCA, we obtained lncRNA expression matrix from the TCGA. Then, necroptosis-related lncRNAs (NRLs) were identified using Pearson correlation analysis (|coefficient | >0.4 and p < 0.001, Wilcoxon test) based on their expression. Subsequently, DE-NRLs were screened using the “limma” package.

### Construction of the NRLs-associated prognostic panel and nomogram

The optimal panel of prognostic NRLs was determined using the LASSO-Cox algorithm, and a corresponding prognostic panel was constructed. In the meantime, we equally divided all patients into training and testing groups, the risk score of each patient with ESCA was calculated using the formula as follows:


$$Risk\;Score=\sum_{i=1}^{n}Coef\left(i\right)\times x\left(i\right)$$

Coef (i) represents the coefficient, and x(i) represents the standardized level of each NRL. According to the median risk score of each patient, all patients were categorized into low-risk and high-risk groups. Subsequently, the predictive capacity of the 6-NRLs prognostic panel was assessed for various clinicopathological characteristics by the log-rank method. Besides, a nomogram was generated, and the 1-, 3- and 5-year recurrence rates of ESCA patients were predicted based on such a nomogram.

### Models comparison

We retrieved the published prognostic signatures of ESCA constructed based on lncRNAs or mRNAs since 2017 using multiple databases. Then, “timeROC” and “survcomp” packages were applied for model comparison to evaluate the predictive ability of each model.

### Pathway enrichment analysis and TMB

The potential enrichment pathways among different risk groups were identified using the GSEA with the “clusterProfiler” package. Moreover, “maftools”, “survival,” and “survminer” were applied to reveal the difference and survival of the TMB between the above-mentioned two groups of patients.

### Identification of potential compounds in the treatment response of ESCA

The “pRRophetic”, “ggpub”, and “ggplot2” packages were used to identify the potential chemotherapeutic drugs that might be applied for ESCA therapy. Moreover, the Wilcoxon signed-rank test was adopted to determine the half-maximal inhibitory concentration (IC50) of common compounds between high-risk and low-risk groups.

### Cell culture and reagents

The human esophageal epithelial cells (HEECs) and human esophageal cell lines, including ECA109, KYSE150, and KYSE510, were maintained in RPMI-1640 medium (Gibco, Thermo Fisher Scientific, USA) supplemented with 10% fetal bovine serum (Gibco, Thermo Fisher Scientific, USA) and penicillin-streptomycin cocktail (1:100; Solarbio, Beijing, China) at 37° C in a humidified atmosphere containing 5% CO_2_. TSZ (TNF-alpha, Smac, and z-VAD) (Selleck, Houston, TX, USA) and necrostatin-1 (MedChemExpress, Monmouth Junction, NJ, USA) were used as previously described [34].

### RNA extraction and RT-qPCR

Total RNA was isolated from the cells using TRIzol Reagent (Invitrogen, Eugene, OR, USA). Subsequently, purified RNA was reversely transcribed to cDNA using HiScript III RT SuperMix (Vazyme, Nanjing, China). RT-qPCR was performed using ChamQ Universal SYBR qPCR Master Mix (Vazyme, Nanjing, China). The PVT1 plasmid was adopted as a positive control, while the negative control contained all components except for cDNA. Each experiment was carried out three times. GAPDH was employed as the housekeeping gene. The relative expression of PVT1 was determined using the 2^^−ΔΔCt^ method. Supplementary Table 3 lists the primer sequences.

### Vector construction and transfections

For PVT1 overexpression, the full-length sequence of PVT1 was synthesized and cloned into the pcDNA3.1(+) vector (Hanbio Biotechnology, Shanghai, China). The shRNAs targeting PVT1 were synthesized by Sangon Biotech Co., Ltd. (Shanghai, China), and Supplementary Table 3 lists the corresponding sequences. Afterward, the transfection assays were carried out using Lipofectamine™ 3000 according to the manufacturer’s instructions (Invitrogen, Thermo Fisher Scientific, USA).

### Migration assay

To conduct migration assays, uncoated transwell inserts with 8 μm pores were inserted into 24-well plates. Next, we added 700 μl of RPMI-1640 medium with 20% FBS to each lower chamber, and a total of 5×104 infected ECA-109, KYSE-150, or KYSE-510 cells were resuspended in 200 μl serum-free medium in each upper chamber. The remaining cells in the upper chamber were wiped clean with a cotton swab after 24 hours of incubation (48 hours for KYSE-510 cells) at 37° C, 5%CO2. The bottom membrane with invaded cells was fixed with 4% paraformaldehyde and dyed with 0.1% crystal violet (Solarbio, Beijing, China) for 30 min, respectively. The migrated cells were counted in five random fields at 200× magnification using ImageJ software. The results were expressed as the mean number of migrated cells per field.

### Colony formation assay

For colony formation assay, 1000 infected ECA-109, KYSE-150, or KYSE-510 cells were inoculated into the six-well plates and incubated for 12 days at 37° C, 5% CO2. When clones were visible to the naked eye, the cells were washed with phosphate-buffered saline (PBS) twice and fixed with 4% paraformaldehyde for 20 min. When clonal colonies were visible (≥ 50 cells/colony), the cells were washed twice with phosphate-buffered saline (PBS) and fixed with 4% paraformaldehyde for 20 minutes, followed by staining with 0.1% crystal violet solution for 30 min. After being air-dried, the colonies were photographed and counted.

### Cell counting kit-8 assay

To evaluate the proliferative capacity of infected cells, a cell counting kit-8 (CCK-8) experiment was conducted. Specifically, infected ECA-109, KYSE-150, or KYSE-510 cells were inoculated into 96-well plates at a density of 3000 cells per well for proliferation for 0, 1, 2, 3, 4, and 5 days, then 10ul CCK-8 reagent (Solarbio, Beijing, China) was added to each well and incubated for 2 h at 37° C. for 2 hours. The absorbance at 450 nm was measured using a microplate reader (Thermo Fisher Scientific, USA).

### Western blotting analysis

Briefly, ESCA cells under different treatments were rinsed with cold PBS two times and then lysed using RIPA buffer (Thermo Fisher Scientific, USA) containing protease inhibitors (Solarbio, Beijing, China) and phosphatase inhibitors (Solarbio, Beijing, China). Moreover, the contents of soluble proteins were determined by the BCA protein detection kit (Thermo Fisher Scientific, USA). Subsequently, Western blotting analysis was carried out as previously described [34] using antibodies against RIP (#3493), phospho-RIP (Ser166) (#65746), MLKL (#14993), phospho-MLKL (Ser358) (#91689), and GAPDH (#8884) (1:1,000; Cell Signaling Technology, Danvers, MA, USA).

### Data availability

The data supporting the conclusions of this study could be acquired from TCGA database (https://portal.gdc.cancer.gov/) and KEGG website (https://www.kegg.jp/). Other details are available from the corresponding author upon a reasonable request.

## RESULTS

### Differentially expressed NRGs

**Figure 1:**
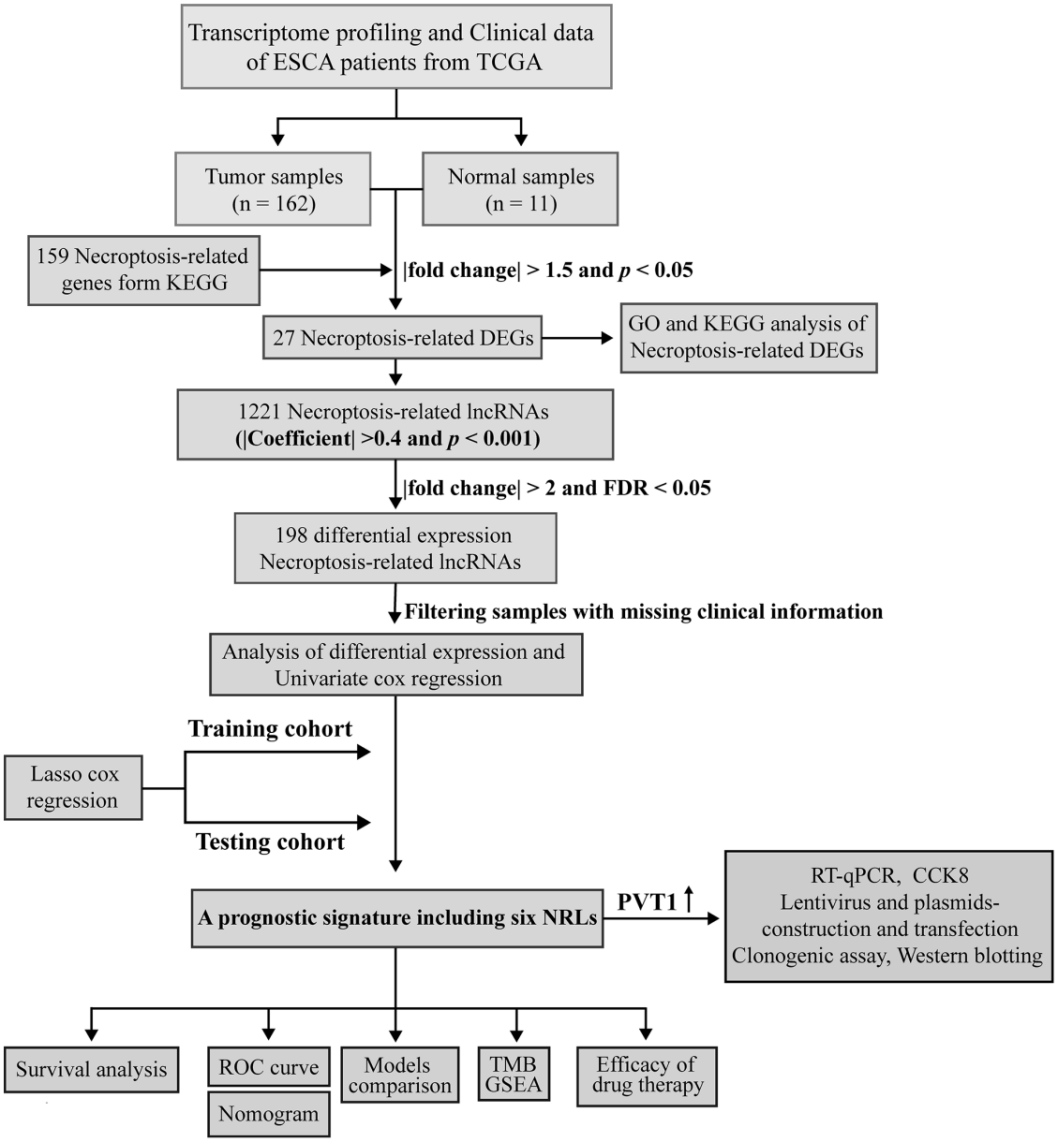
Workflow based on a comprehensive analysis of NRLs.

Figure 1 illustrates the flowchart for the current work. Supplementary Table 2 shows the clinical details of ESCA patients. We identified 27 DE-NRGs between the tumor and adjacent normal tissues (|fold change| > 1.5 and FDR < 0.05) (Supplementary Figure 1A and Supplementary Table 4). Moreover, we performed enrichment analyses of the DE-NRGs using the Metascape database. Unsurprisingly, these NRGs primarily participated in necroptosis, influenza A, measles virus infection, TNF-α signaling pathway, and so on (Supplementary Figure 1B), and a PPI network of DE-NRGs was established (Supplementary Figure 1C and Supplementary Table 7). Lastly, we found that the genetic alteration of seven NRGs exhibited a mutation rate of ≥3% using cBioPortal, among which FADD had the highest mutation rate (14%) (Supplementary Figure 1D).

### Construction of an NRLs-based prognostic signature in ESCA

**Figure 2:**
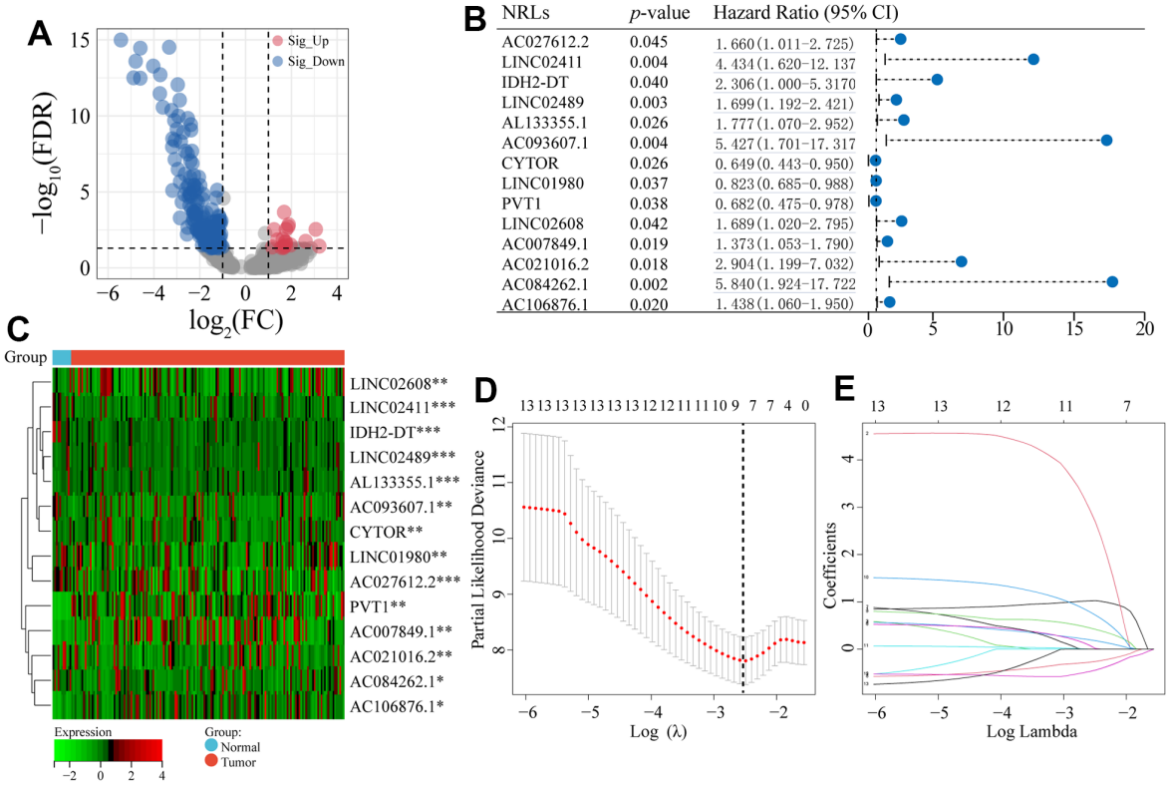
**Differentially co-expressed NRLs and LASSO regression.** (**A**) The volcano plot of the significant differential expression of NRLs. (**B**, **C**) A forest plot and a heatmap of the 14 prognostic NRLs. (**D**) Ten-fold cross-validation for error rate. (**E**) Least absolute shrinkage and selection operator regression. Asterisks (*) stand for significance levels, ** p < 0.01, * p < 0.05.

**Figure 3:**
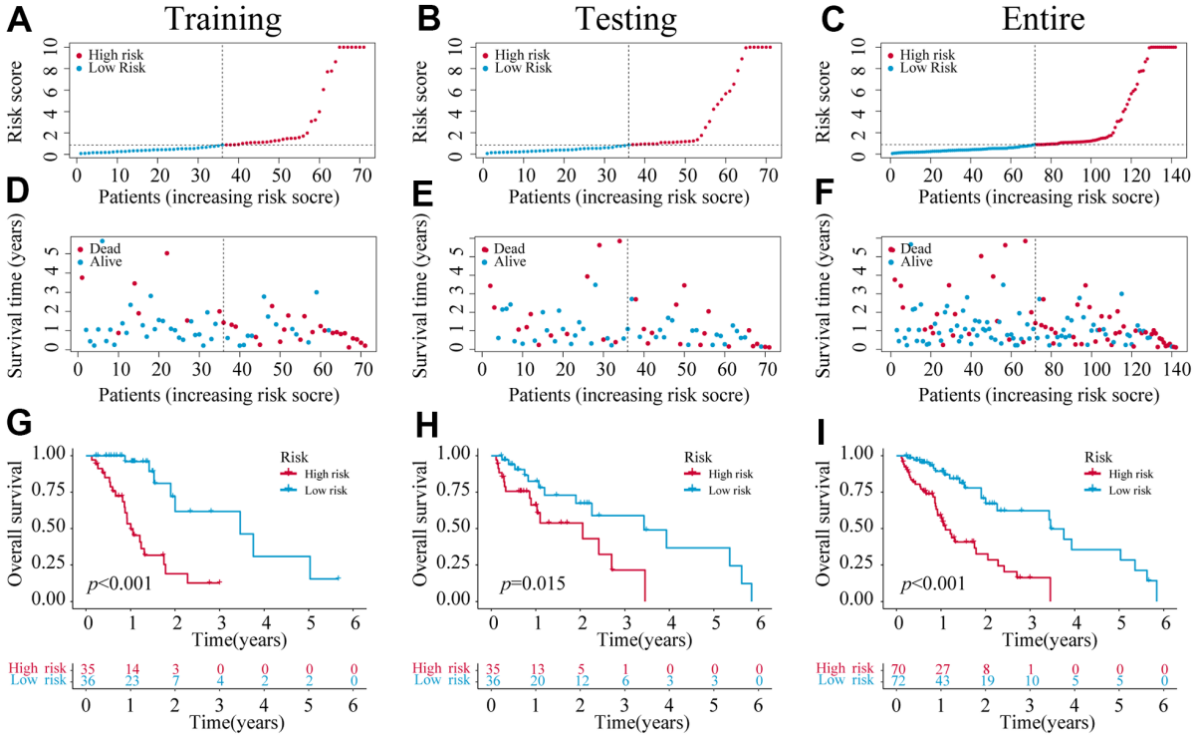
**The construction of a 6-NRLs prognostic signature.** (**A**–**C**) The risk score of six prognostic NRLs. (**D**–**F**) Survival status distribution of six prognostic NRLs. (**G**–**I**) Kaplan–Meier survival curves of high-risk and low-risk patients in training, testing, and entire groups.

We identified 1,221 NRLs with a co-expression correlation in ESCA (Supplementary Table 5). Among them, 198 DE-NRLs were identified between the ESCA and adjacent normal tissues, including 20 up-regulated and 178 down-regulated NRLs (|fold change| > 2 and FDR < 0.05) (Figure 2A). Subsequently, the prognostic significance of NRLs was shown by the univariate Cox regression and heatmap (Figure 2B, 2C). Subsequently, a prognostic panel consisting of 6 NRLs was constructed using the LASSO algorithm and multivariate Cox regression analysis (Figure 2D, 2E). The risk score = (7.7678) × AC027612.2 + (0.6060) × IDH2-DT + (0.5578) × PVT1 + (0.7428) × LINC02608 + (-1.0974) × AC021016.2 + (1.6266) × AC084262.1). The ESCA patients were grouped based on their median risk scores. Figure 3 exhibits the status of survival, the distribution of risk scores, and the overall survival (OS) of six NRLs in the training, testing, and entire cohort. The ESCA patients with lower risk scores exhibited a reduced risk of death and longer survival time (Figure 3G–3I). Importantly, compared with AGs, NRGs, and NRLs, PCA indicated that the 6-risk NRLs had optimal discrimination capacity, which could better distinguish high- and low-risk groups (Supplementary Figure 2).

### Assessment of clinicopathological indicators of the NRLs panel

**Figure 4:**
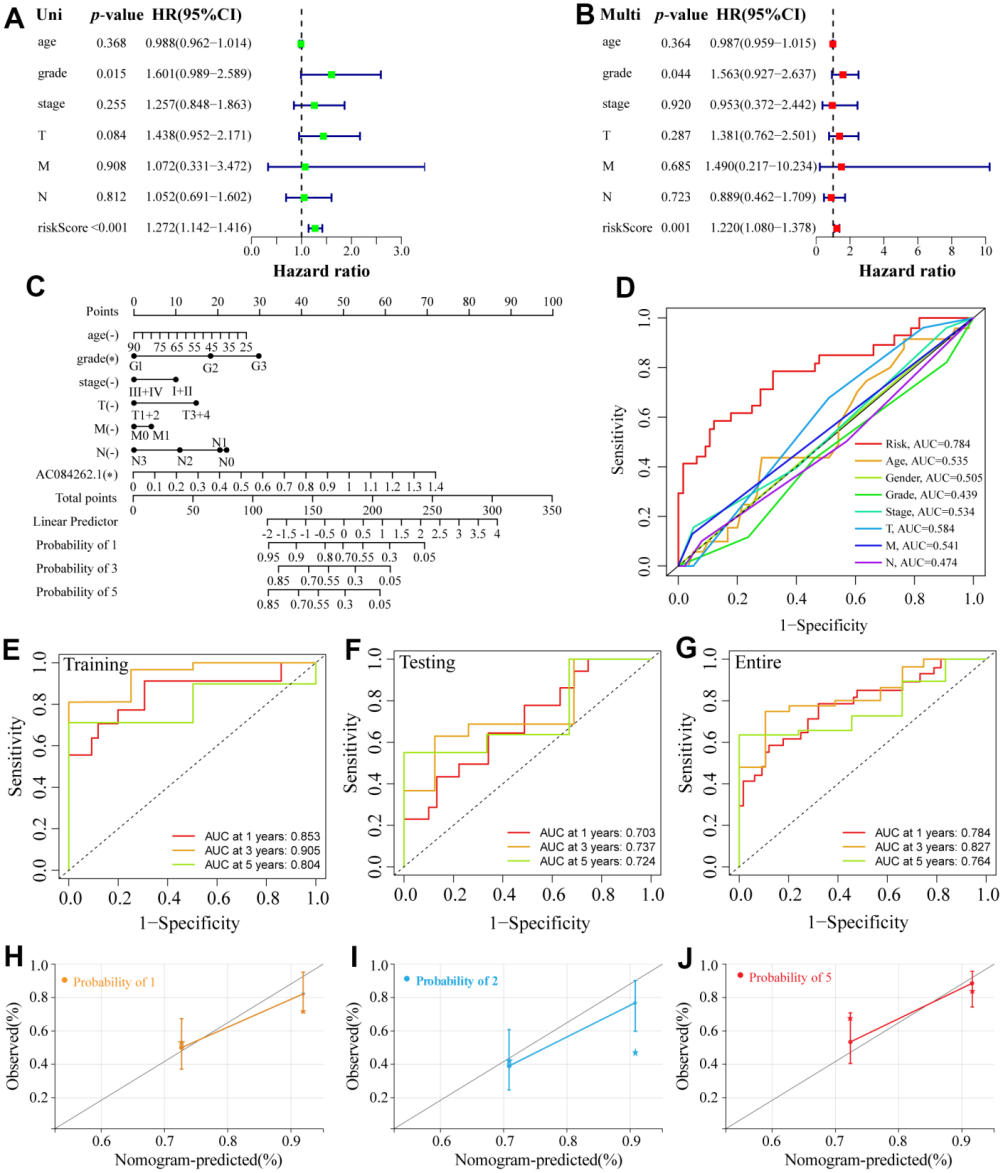
**Establishment of a nomogram and ROC analysis.** (**A**, **B**) The forest plot of the univariate and multivariate Cox regression. (**C**) A nomogram combining clinicopathological variables and risk score predicts 1-, 3-, and 5-year OS of ESCA patients. (**D**) The ROCs curve for different clinicopathological variables. (**E**–**G**) The ROCs curve for 1-, 3-, and 5-year survival time training, testing, and entire groups are based on the risk score. (**H**–**J**) Calibration curve of the nomogram to predict the probability of the 1-, 3-, and 5-year OS.

A forest plot was established to further determine independent prognostic indicators for ESCA patients based on univariate and multivariate Cox analyses (Figure 4A, 4B). Our results revealed that grade was an independent factor influencing the prognosis of ESCA. Next, the 1-, 3- and 5-year overall recurrence rates in ESCA patients were predicted using a nomogram containing clinicopathological features (Figure 4C). In addition, our data indicated that the area under ROC curves (AUCs) of 1-year in the entire cohort was 0.784 (Figure 4D), which performed better than other clinicopathological features in the prognostic prediction of ESCA patients. Besides, the 3- and 5-year AUC for the entire cohort were 0.827 and 0.764, respectively (Figure 3G). Moreover, the AUCs of both training and testing sets also displayed good predictive performance. In the training cohort, the AUCs of 1-, 3-, and 5-year survival were 0.853, 0.905, and 0.804, respectively (Figure 4E). In the testing cohort, the AUCs of 1-, 3- and 5-year survival were 0.703, 0.737, and 0.724, respectively (Figure 4F). Finally, the calibration curves displayed good consistency between the actual and predicted survival possibility at 1-, 3-, and 5 years (Figure 4D–4J). Subsequently, we assigned ESCA patients into high- and low-risk groups based on age, gender, grade, stage, T, and N. For each clinicopathological variable, the patients with high risks had a markedly reduced survival time and higher mortality prevalence compared with the low-risk group (Supplementary Figure 3).

### Evaluation of the NRLs signature

**Figure 5:**
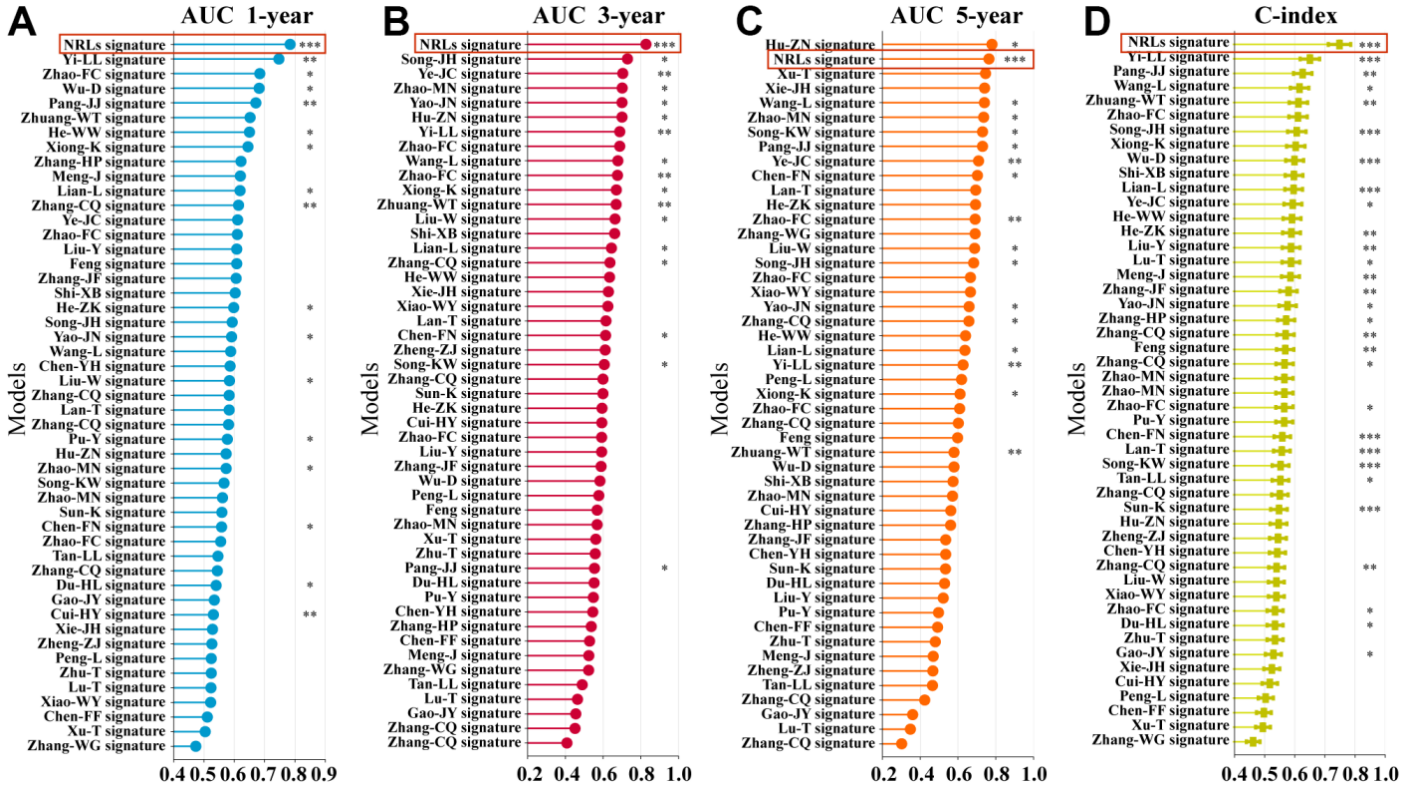
**Comparison of multiple prognostic models.** (**A**–**C**) Comparison of AUC in multiple prognostic models at 1-, 3-, and 5-year. (**D**) C-index comparison of six NRLs prognostic features with other prognostic features.

A total of 48 prognostic signatures for ESCA based on lncRNA or mRNA were included in the comparison (Supplementary Table 6). The results showed that the 1- and 3-year AUC of the six-NRLs panel for the entire cohort were higher than those of other prognostic models, respectively (Figure 5A, 5B), and 5-year AUC was higher than those of most prognostic models (Figure 5C). Furthermore, we assessed NRLs discrimination by C-index. Our data displayed that the C-index [95% confidence interval] of six NRLs was 0.759 [0.721, 0.797], which was superior to other prognostic models (Figure 5D).

### Pathway enrichment analysis

To elucidate the underlying molecular mechanism of the 6-NRLs prognostic panel in ESCA, we investigated critical pathways between the two groups via GSEA. We found that the citrate and TCA cycle, mTOR signaling pathway, and oxidative phosphorylation were remarkably enriched in the high-risk group (Supplementary Figure 4A), while the notch signaling pathway, antigen processing and presentation, pentose phosphate pathway, and TGF-β and hedgehog signaling pathway were enriched in the low-risk group (Supplementary Figure 4B).

### TMB analysis

**Figure 6:**
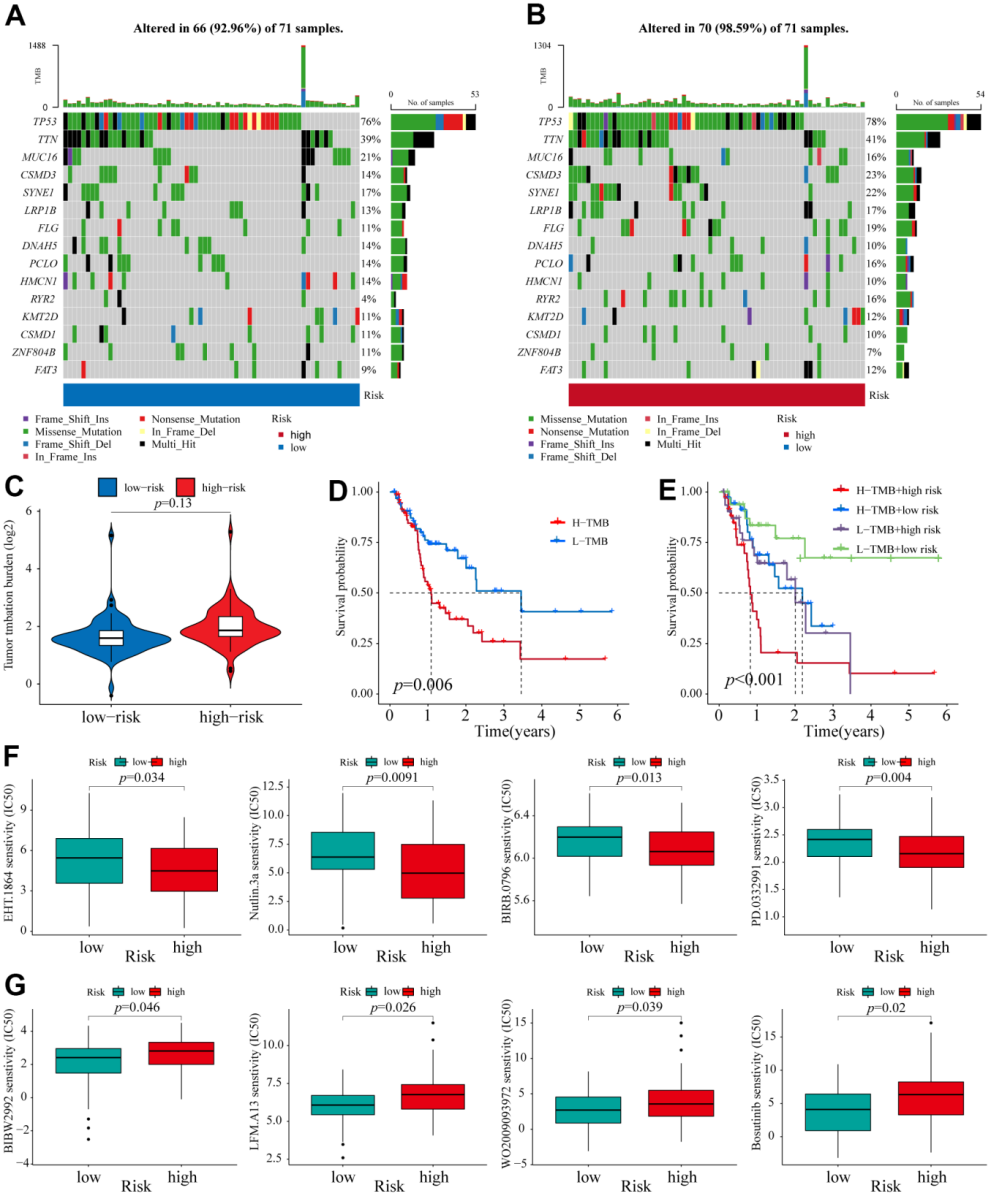
**TMB analysis and Identification of potential compounds.** (**A**, **B**) The waterfall plot of the mutation landscape of the top 15 genes with high mutation frequencies in the low- and high-risk groups. (**C**) TMB difference of patients in low- and high-risk groups. (**D**) Survival analysis for patients in high- and low-TMB. (**E**) Survival analysis for patients classified based on the TMB and 6-NRLs risk signature. The drug sensitivity of the high-risk group was higher than those of the low-risk group. (**F**) EHT.1864, Nutlin.3a, BIRB.0796, and PD.0332991. The drug sensitivity of the low-risk group was higher than those of the high-risk group. (**G**) BIBW2992, LFM.A13, WO2009093972, and Bosutinib.

Accumulating evidence has revealed that TMB has become a promising biomarker for the immune response to malignancies [35, 36]. To assess the correlation between different risk groups and TMB, we calculated TMB scores of high and low-risk groups (the top 15 genes with the highest mutation) according to TGCA somatic mutation data. We found that the high-risk group had a higher mutation frequency for most genes than the low-risk group, except for MUC16, DNAH5, HMCN1, ZNF804B, and CSMD1 (Figure 6A, 6B). However, as a whole, no significant difference in TMB was found between these two groups (Figure 6C). Next, the ESCA patients were assigned into high- and low-mutation groups based on the TMB scores. We found that the high-mutation group had a reduced survival possibility compared with the low-mutation group (Figure 6D). Interestingly, we confirmed that the 6-NRLs panel performed better than TMB in the prognostic prediction and found that integrating two signatures might be a more appropriate clinical strategy (Figure 6E).

### Identification of potential compounds for therapeutic response to ESCA

Eight therapeutic compounds exhibited marked differences in drug sensitivity between high- and low-risk groups, indicating that the IC50 values of four drugs, EHT.1864 (RAC family inhibitor), Nutlin.3a (Rebemadlin), BIRB.0796 (Doramapimod), and PD.0332991 (Palbociclib), in the low-risk group, were higher compared with the high-risk group (Figure 6F), while the other four drugs, BIBW2992 (Afatinib), LFM.A13 (BTK inhibitor), WO2009093972 (PI3K inhibitor), and Bosutinib, had higher sensitivity compared with the low-risk group (Figure 6G). These findings contributed to exploring better personalized therapeutical strategies.

### Effects of PVT1 on the proliferation, migration, and necroptosis of ESCA cells

**Figure 7:**
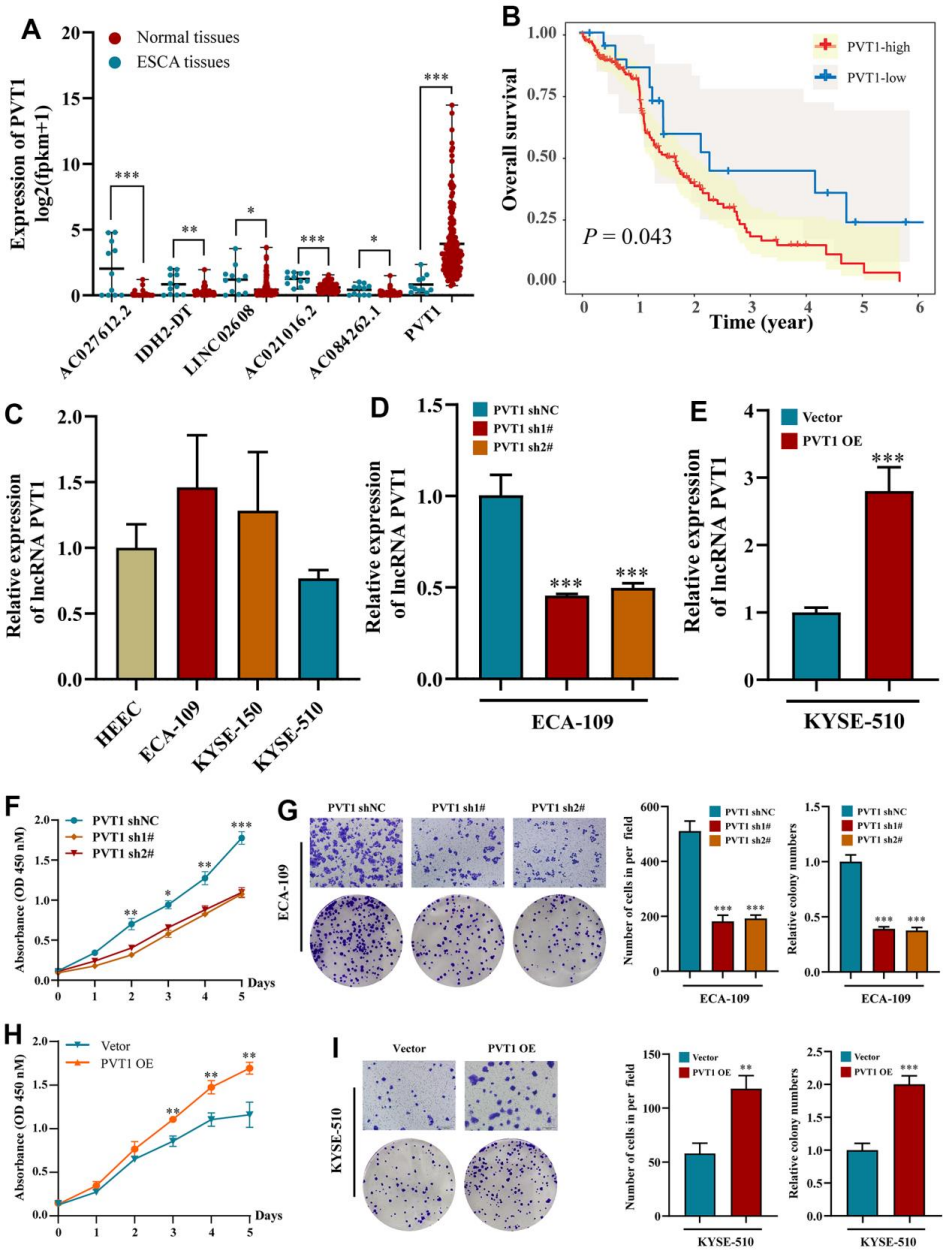
**Effects of inhibiting the expression of PVT1 in cell proliferation and migration *in vitro*.** (**A**) The expression of PVT1 in normal and ESCA tissues. (**B**) KM survival analysis for ESCA patients with different PVT1 expressions; (**C**) The expression of PVT1 in HEEC and ESCA cells; (**D**) The transfection efficiency of PVT1-sh1#/sh2# in ECA-150 cell; (**E**) The transfection efficiency of PVT1-OE in KYSE-510 cell; (**F**, **G**) Knockdown of PVT1 inhibited ECA-150 cell proliferation and colony formation ability. (**H**, **I**) Overexpression of PVT1 promoted KYSE-510 cell proliferation and colony formation ability. Data are presented as mean ± SD. (*p < 0.05, **p < 0.01, ***p < 0.001).

**Figure 8:**
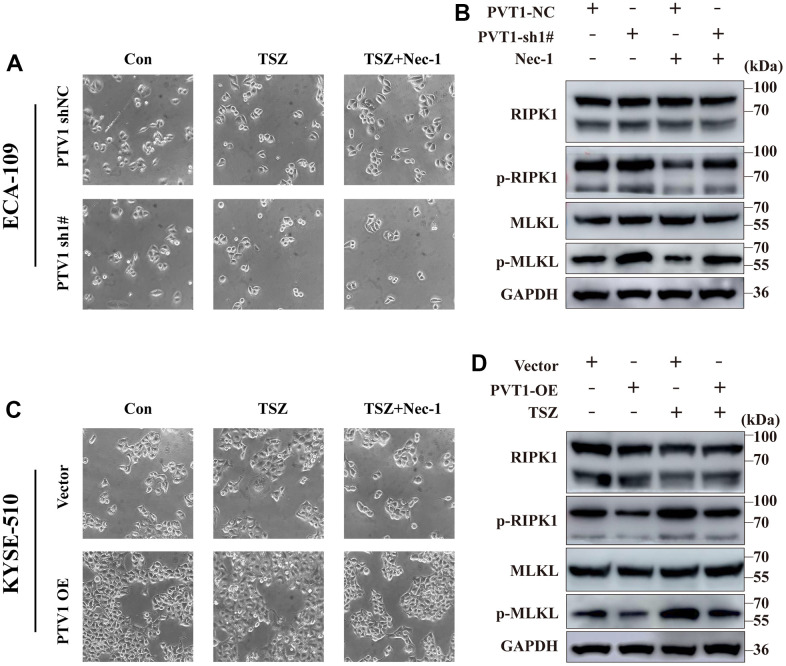
**Induction of necroptotic cell death in PVT1-sh#/ PVT1-OE ESCA cells.** (**A**) ECA-109 cells were treated with Nec-1 (50 μM) for 4 h and then treated with TSZ (30ng/ml TNF-α, 200nM Smac mimetics, 20μM zVAD). After 24 h of drug treatment, the morphological changes of treated cells were imaged under a phase-contrast microscope. (**B**) Western blot from ECA-109 cell was performed to detect RIP1, p-RIP1, MLKL, and p-MLKL protein levels. (**C**) KYSE-510 cells were treated with Nec-1 (50 μM) for 4 h and then treated with TSZ (30ng/ml TNF-α, 200nM Smac mimetics, 20μM zVAD). After 24 h of drug treatment, the morphological changes of treated cells were imaged under a phase-contrast microscope. (**D**) Western blot KYSE-510 cell was performed to detect RIP1, p-RIP1, MLKL, and p-MLKL protein levels.

Consistent with the above results, only the expression of PVT1 was increased in ESCA tissues among the six prognostic NRLs compared with normal tissues (Figure 7A). High expression of PVT1 was positively correlated with the poor outcomes of ESCA patients (Figure 7B). To explore the potential roles of PVT1 in the progression of ESCA, we first assessed the expression pattern of PVT1 in normal HEECs and available ESCA cell lines (ECA-109, KYSE-150, and KYSE-510). Compared with HEEC cells, ECA-109 and KYSE-150 showed high expression of PVT1, while KYSE-510 exhibited low expression of PVT1 (Figure 7C). Accordingly, ECA-109 and KYSE-150 were selected for deletion of PVT1, KYSE-510 was chosen for overexpression of PVT1, and the transfection efficiencies were estimated by RT-qPCR (Figure 7D, 7E and Supplementary Figure 5A). *In vitro* experiments revealed that depletion of PVT1 significantly inhibited the proliferation and migration of ECA-109 and KYSE-150 cells compared with the controls (Figure 7F, 7G and Supplementary Figure 5B, 5C), while overexpression of PVT1 in KYSE-510 displayed the opposite results (Figure 7H, 7I). To further confirm whether PVT1 was an essential regulator for necroptosis, we observed the necroptosis of ESCA cells treated with TSZ (1:1,000) and Nec-1 (50 μm/mL). Figure 8A reveals that compared with the control group, more ECA-109 PVT1-sh cells underwent necroptosis, and this phenotype was strengthened upon TSZ treatment, while Nec-1 alleviated such necroptotic death. Likewise, the Western blotting analysis exhibited that depletion of PVT1 increased the expressions of p-RIP1 and p-MLKL at the protein level (Figure 8B), and similar results were confirmed in KYSE-150 PVT1-sh cells (Supplementary Figure 5D, 5E). In contrast, overexpression of PVT1 inhibited necroptotic cell death, while TSZ significantly promoted necroptotic phenotype in ECA-109 PVT1-sh cells (Figure 8C), and overexpression of PVT1 reduced the expressions of p-RIP1 and p-MLKL at the protein level (Figure 8D). Taken together, these findings indicated the underlying role of PVT1 in the necroptosis of ESCA cells.

## DISCUSSION

This work put forward several new revelations. First, a 6-NRLs signature was constructed and internally validated for predicting the prognosis of ESCA. Second, we discovered the good accuracy and stability of 6-NRLs panel using multi-models comparison. Third, we have confirmed that lncRNA PVT1 can inhibit necroptosis, thus promoting the progression of ESCA.

As a regulated form of necrosis, necroptosis can result in organelle swelling, cell membrane rupture, and decomposition of cytoplasm and nucleus [37]. Accumulating evidence reveals that necroptosis participates in the pathogenesis of various malignancies and plays a fundamental role in the prognosis and treatment response of patients [38]. Therefore, targeting necroptosis has emerged as a potential antitumor strategy [39]. Recently, some lncRNA-based signatures related to necroptosis have been used to evaluate the outcomes of patients with various tumors, including lung adenocarcinoma [23], colon cancer [25], and stomach cancer [24]. However, the prognostic value of NRLs in ESCA has not been well documented.

In the present study, a total of 27 differentially expressed NRGs was identified, many of these DE-NRGs may contribute to activating necroptosis, such as IL33, GLUL, and RIPK1. Interestingly, we observed that compared with normal tissues, these genes were down-regulated in ESCA tissues, suggesting that necroptosis inhibition may be a driving factor mediating the development of ESCA. Next, we chose 14 prognosis-related NRLs from 198 DE-NRLs, and then a prognostic model was constructed using six NRLs (AC027612.2, IDH2-DT, PVT1, LINC02608, AC021016.2, and AC084262.1) based on LASSO and multi-Cox regression. Simultaneously, a nomogram, internal testing cohort, PCA, and TMB analysis were adopted to assess the validity of the prognostic panel. Besides, we further investigated the predictive capability of this prognostic model in various clinicopathological features and multiple prognostic signatures. The results showed that regardless of age, sex, grade, and other clinicopathologic indicators, the prognosis model could effectively divide ESCA patients into low- and high-risk groups. On the other hand, this 6-NRLs panel has more predictive power than other prognostic models. Overall, our results showed that the 6-NRLs prognostic panel had a good performance in predicting the prognosis of ESCA patients.

As one of the most common malignant tumors worldwide, ESCA originates from the esophageal mucosa epithelium. Some studies have confirmed that the dysregulation of lncRNAs is extensively involved in the biological processes of ESCA, including proliferation, apoptosis, metastasis, angiogenesis, and treatment resistance [19, 40, 41]. These findings indicate that lncRNAs have great clinical value and are candidate biomarkers for early diagnosis, treatment responses, and clinical outcomes. Although some lncRNA-related prognostic models in ESCA have been constructed, there is still a lack of effective evaluation of the prognostic models of ESCA [42–44]. Toll-like receptor (TLR) activates necroptosis through the interaction between TRIF and necrosome [45]. Liu et al. [46] have reported a promising 4-lncRNA prognostic signature for ESCA. In the present work, we identified 198 DE-NRLs between adjacent normal and ESCA tissues and constructed a 6-NRLs prognostic signature using LASSO-Cox regression. Among them, neither AC027612.2 nor LINC02608 have been previously reported in ESCA or other diseases. Studies have found that the combination of seven lncRNAs, including AC021016.2, AC079630.1, AC116407.1, and so on, can predict the prognosis of lung adenocarcinoma (LUAD) patients [47]. Interestingly, they have found that AC021016.2 also exhibits significant prognostic value in the validation cohort [47]. It has been reported that a prognostic scoring feature containing AC084262.1 has the potential to predict the clinical outcomes of ESCC, supporting our findings [48]. Increasing evidence has shown that IDH mutations have vital implications for the progression and treatment of various malignancies [49]. Zheng et al. [50] have found that wild-type IDH2 contributes to acute myeloid leukemia (AML) via inducing the conversion of α-KG to isocitrate for lipid synthesis and promoting c-Myc expression. These findings provide the possibility for targeting therapy of metabolic vulnerability. Additionally, it seems that the combination of enasidenib and azacitidine can be used as a feasible strategy for treating AML patients harboring IDH2 mutations [51]. Furthermore, previous reports have confirmed that PVT1 is widely involved in the proliferation, invasion, metastasis, and multidrug resistance of digestive tract tumor cells, such as esophageal adenocarcinoma [52], colorectal cancer [53], gallbladder cancer [54], and pancreatic cancer [55]. Numerous evidences have revealed that exosomal non-coding RNAs have significant clinical values in disease diagnosis and prognosis, and have become one of that new frontier biomarkers of cancer liquid biopsy [56]. Studies shown that aberrant expression of exosomal lncRNA PVT1 may contribute to disease progression [57, 58]. Collectively, PVT1 has become an emerging biomarker for early screening, efficacy evaluation, and prognostic prediction. Nevertheless, though many efforts have been made in NRLs research, the underlying mechanisms of NRLs in ESCA remain largely unexplored.

To further investigate the underlying molecular mechanisms of the signature, the GSEA was carried out between different risk groups. The signaling pathways related to metabolisms, such as the TCA cycle, ribosome, glycosaminoglycan degradation, mTOR signaling pathway, and primary bile acid biosynthesis, were mainly enriched in the high-risk group. Conversely, TGF, notch, pentose phosphate, and Hedgehog pathways were enriched in the low-risk group. It has been found that the inhibition of mitochondrial oxidative phosphorylation mediated via clomipramine helps restrain ESCC progression [59]. The mutations of key regulators in the mTOR pathway significantly affect the survival and prognosis of cancer patients [60]. Studies have shown that ipriflavone and apatinib enhance the chemosensitivity of ESCC through mTOR-related signaling pathways [61, 62]. Furthermore, notch and Hedgehog pathways are associated with the radioresistance of ESCC [63]. Finally, we predicted some compounds that might be used to treat patients of different risk groups. For instance, Nutlin.3a (Rebemadlin) is an effective MDM2 inhibitor, and inhibition of MDM2 can stabilize p53 protein and thus induce autophagy and apoptosis [64]. Our results indicate that high-risk ESCA patients may be more sensitive to Nutlin.3a (Figure 6F), suggesting that Nutlin.3a could be used as a treatment for these patients. Taken together, these findings supported better individualized therapeutic strategies for ESCA patients.

In addition, the functional phenotype of PVT1 in ESCA cell lines was explored through experimental studies. We first verified the expression level of PVT1 from the TCGA database and cell lines. *In vitro* analysis revealed that overexpression of PVT1 facilitated the proliferation, migration, and colony formation in ESCA cells, and depletion of PVT1 effectively alleviated this phenotype. Interestingly, inhibiting PVT1 increased the expressions of p-RIP1 and p-MLKL and enhanced necroptosis, suggesting that PVT1 was a potential NRLs in ESCA.

However, our research has some limitations. First, our results were mainly based on the TCGA dataset. Therefore, other large-scale case series data and clinical samples for external verification are required to further evaluate the applicability of the signature. Second, we only verified that PVT1 was a possible oncogenic lncRNA, while the specific mechanism of necroptosis regulating ESCA is still unclear, and the remaining NRGs and NRLs and more detailed molecular mechanisms need to be further proved via *in vitro* and *in vivo* experiments.

## CONCLUSIONS

In this study, we constructed a promising prognostic panel consisting of six NRLs, which was not only an independent predictor but also a potential therapeutic target. Furthermore, we also found that PVT1 was a potential regulator of necroptosis, and it participated in the development of ESCA. Collectively, our current findings provided valuable insights into the tumor progression and clinical outcomes of ESCA patients.

## Supplementary Material

Supplementary Figures

Supplementary Table 1

Supplementary Tables 2 and 3

Supplementary Table 4

Supplementary Table 5

Supplementary Tables 6 and 7
