# Supplementary material for: Identification of hub necroptosis-related lncRNAs for prognosis prediction of esophageal carcinoma
Source: Aging (Albany NY). 2023 Jun 1;15(11):4794–819. doi: 10.18632/aging.204763 (PMC10292891; doi:10.18632/aging.204763)
Supplement: Supplementary Figures [file aging-15-204763-s001.pdf]

SUPPLEMENTARY FIGURES

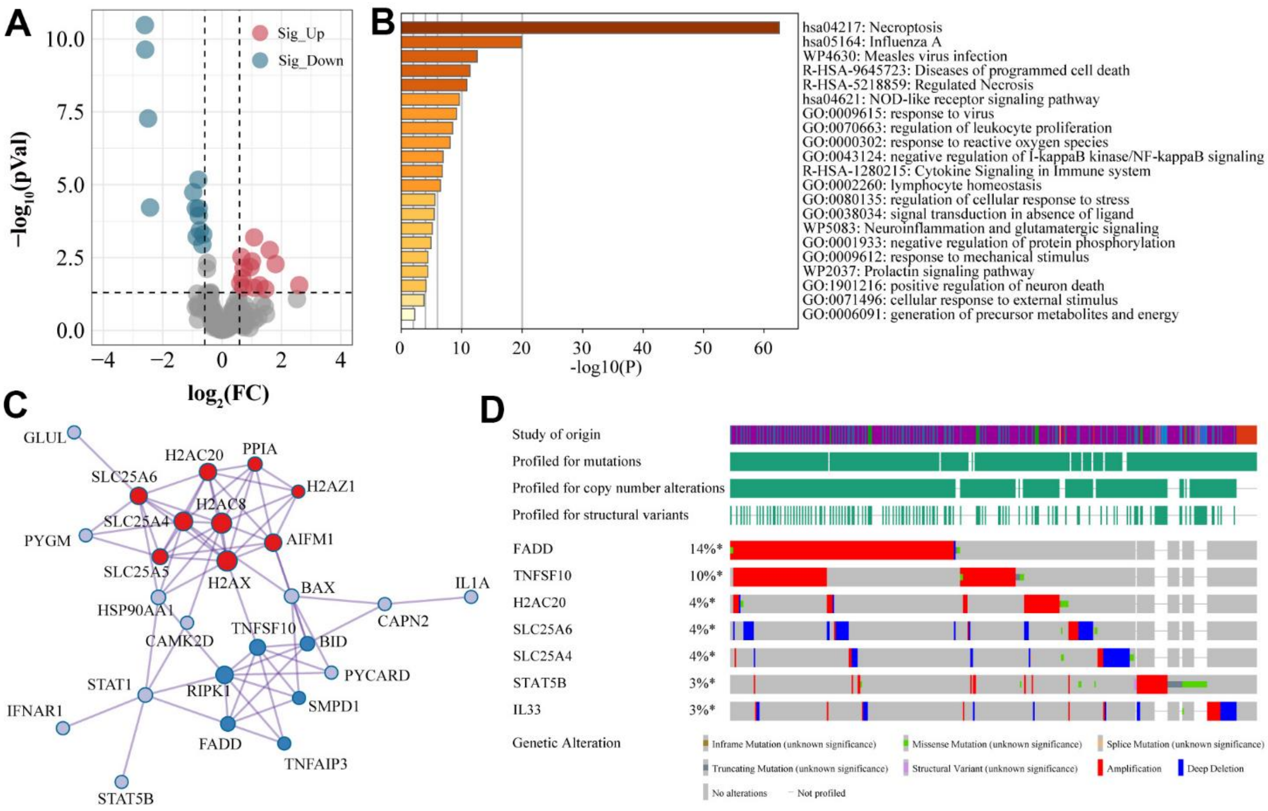

Supplementary Figure 1. The differentially expressed, interaction, and mutation analysis of NRGs. (**A**) The volcano plot of the significantly different expression of NRGs in ESCA and adjacent tissues. (**B**) KEGG enrichment analysis of differentially expressed NRGs. (**C**) A PPI network showed interactions of NRGs. (**D**) A total of seven NRGs have a mutation rate of ≥3%.

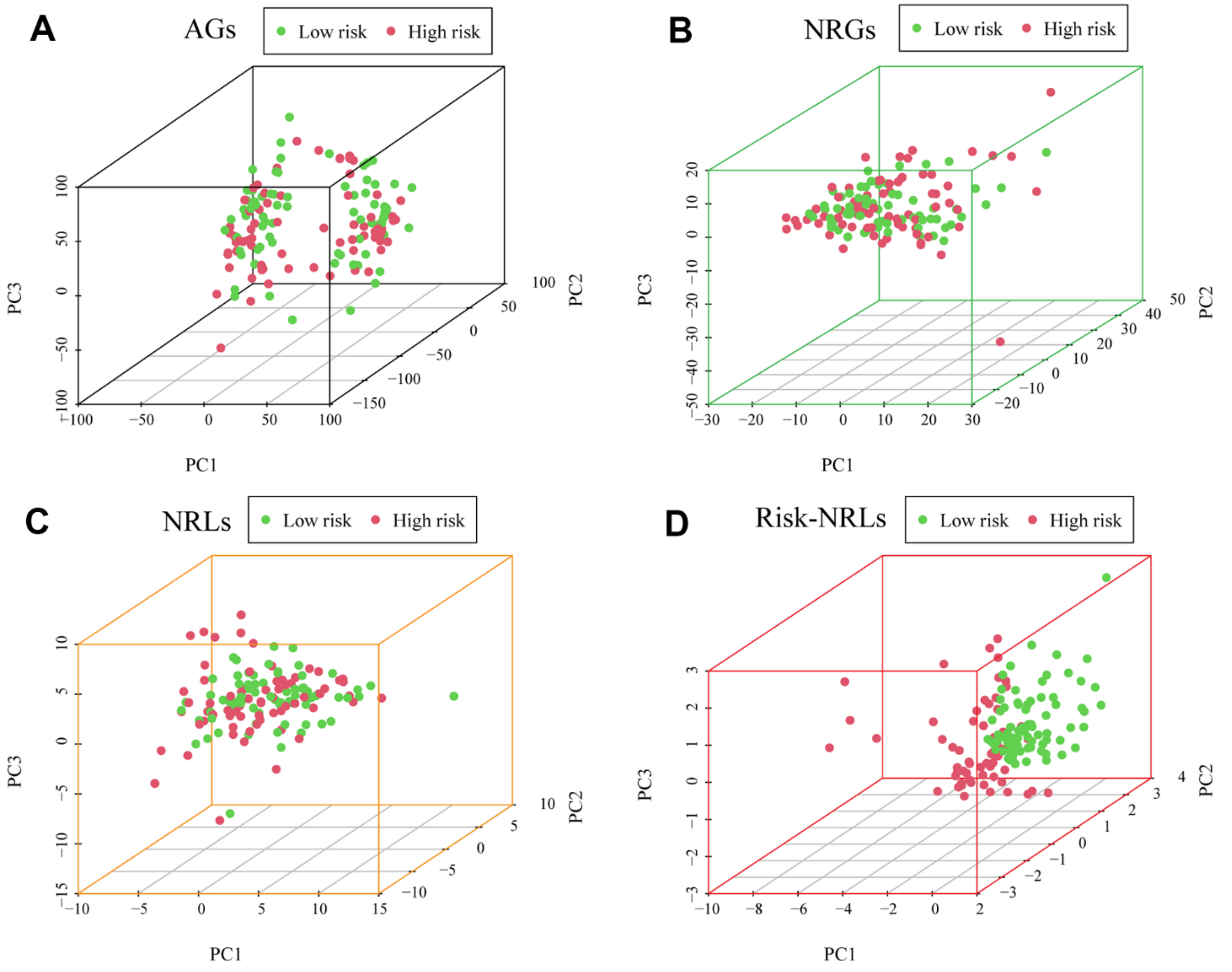

Supplementary Figure 2. Comparison of PCA models based on different gene sets. (**A**) PCA of all genes. (**B**) PCA of all necroptosis-related genes. (**C**) PCA of all co-expressed necroptosis lncRNAs. (**D**) PCA of six prognostic necroptosis-related lncRNAs.

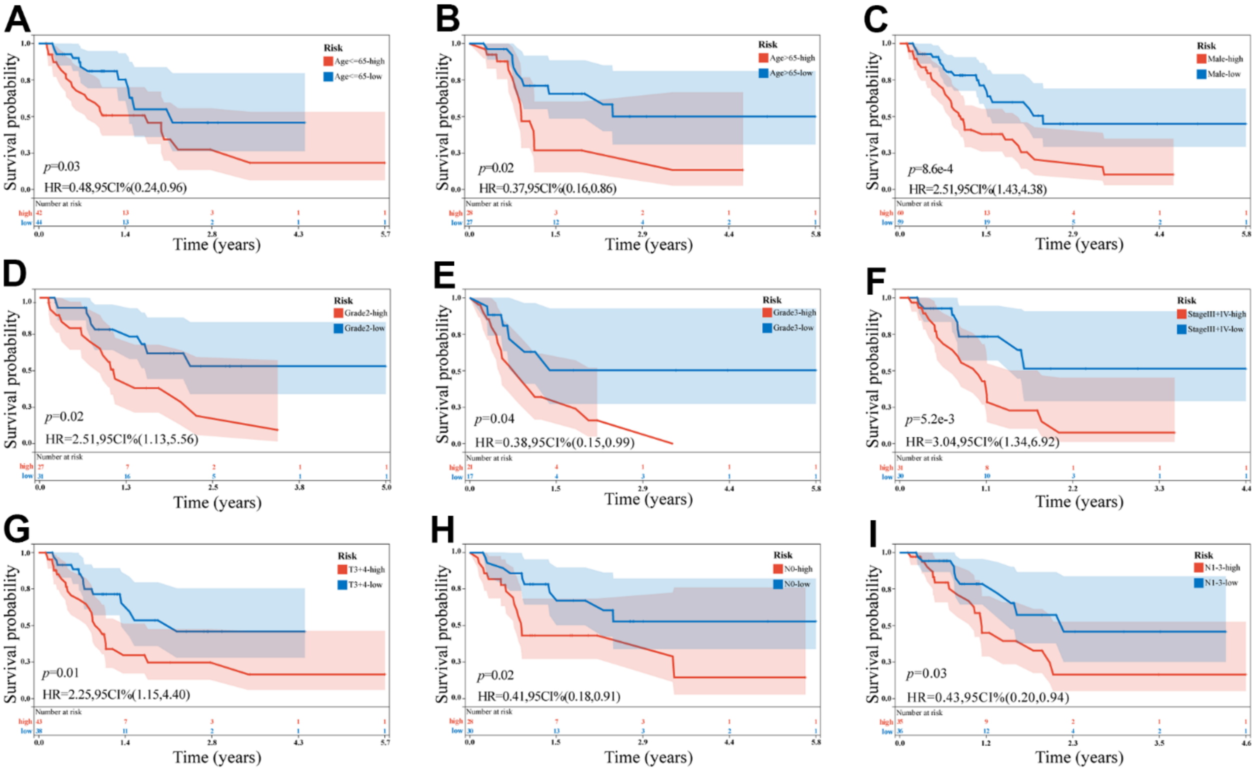

Supplementary Figure 3. Survival analysis of patients in high- and low-risk groups based on various clinicopathological features. Kaplan–Meier survival curves of (**A**, **B**) Age. (**C**, **D**) Male. (**D**, **E**) Grade. (**F**) Stage. (**G**) T. (**H**, **I**) N.

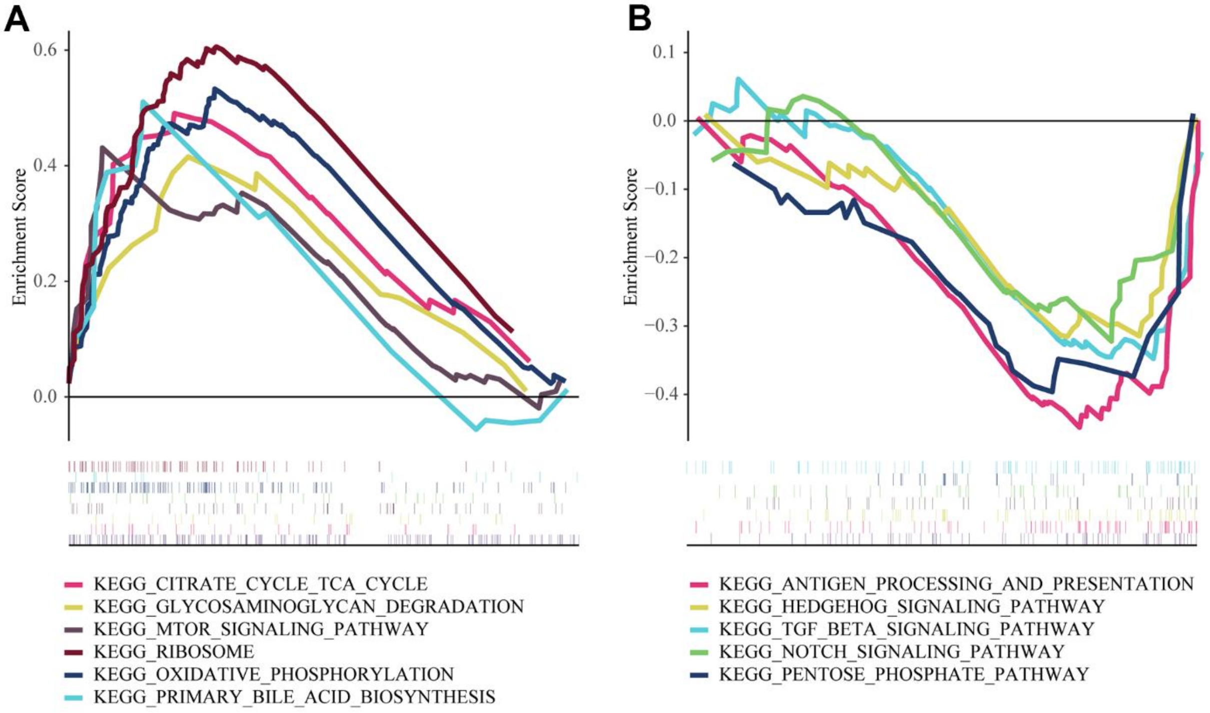

Supplementary Figure 4. GSEA enrichment analysis in ESCA patients from distinct risk groups. (**A**) GSEA enrichment analysis of ESCA patients in the high-risk group. (**B**) GSEA enrichment analysis of ESCA patients in the low-risk group.

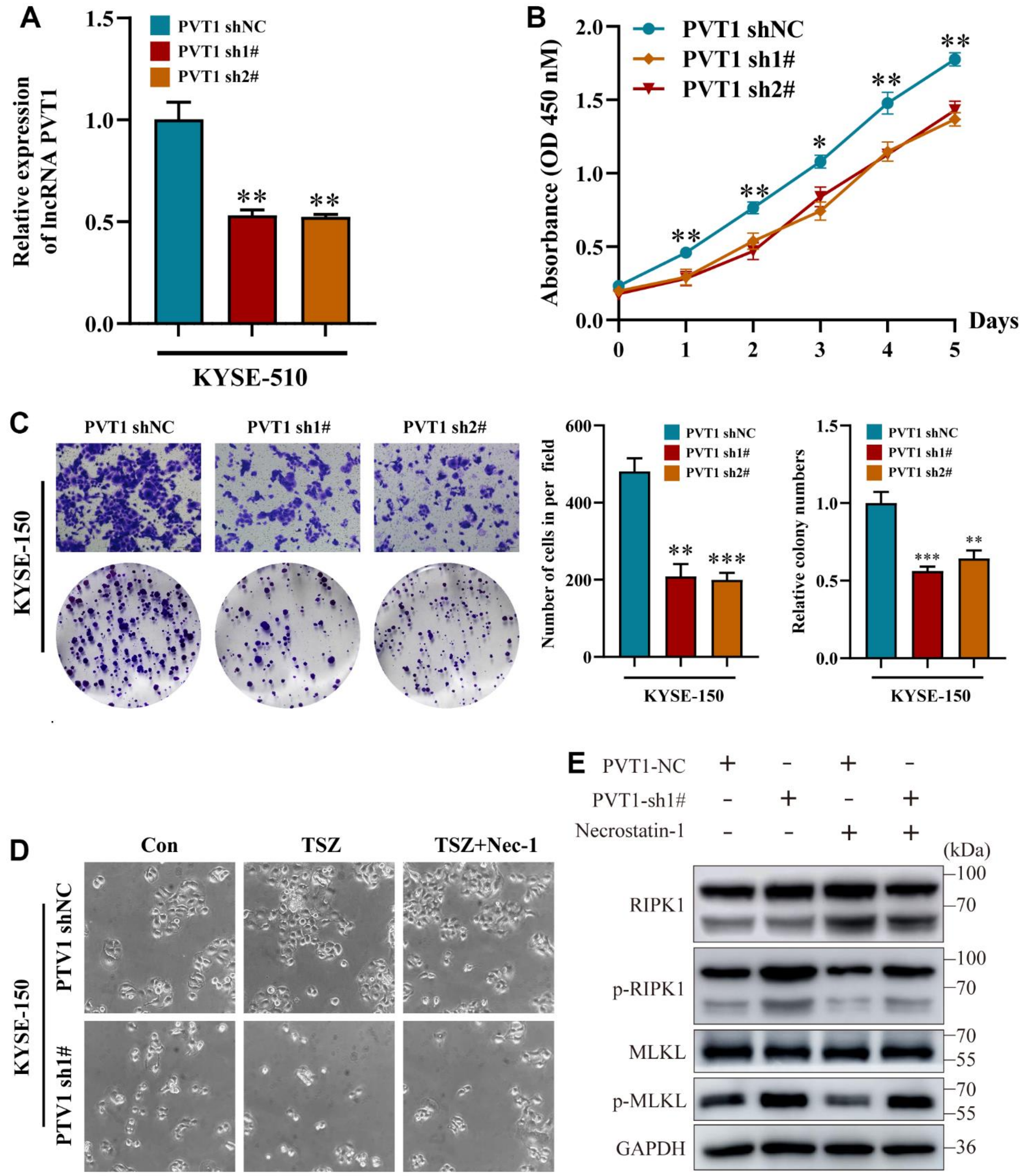

Supplementary Figure 5. PVT1 promoted KYSE-150 cell proliferation, migration, and inhibited necroptosis *in vitro*. (**A**) The transfection efficiency of PVT1 was downregulated using shRNAs. (**B**, **C**) Knockdown of PVT1 inhibited KYSE-150 cell proliferation and colony formation ability. (**D**) Both control and KYSE-150 PVT1-sh cells were treated with Nec-1 (50 μM) for 4 h and then treated with TSZ. After 24 h of drug treatment, the morphological changes of treated cells were imaged under a phase-contrast microscope. (**E**) Western Blot was performed to detect RIP1, p-RIP1, MLKL, and p-MLKL protein levels. Data are presented as mean ± SD (*p < 0.05; **p < 0.01; ***p < 0.001).
