# Supplementary material for: Identification of hub necroptosis-related lncRNAs for prognosis prediction of esophageal carcinoma
Source: Aging (Albany NY). 2023 Jun 1;15(11):4794–819. doi: 10.18632/aging.204763 (PMC10292891; doi:10.18632/aging.204763)
Supplement: Supplementary Table 1 [file aging-15-204763-s002.docx]

**Supplementary Table 1. A total of 159 necroptosis-related genes from KEGG.**

| 1 | TNF | tumor necrosis factor |
| --- | --- | --- |
| 2 | TNFRSF1A | TNF receptor superfamily member 1A |
| 3 | TRADD | TNFRSF1A associated via death domain |
| 4 | TRAF2 | TNF receptor associated factor 2 |
| 5 | TRAF5 | TNF receptor associated factor 5 |
| 6 | RIPK1 | receptor interacting serine/threonine kinase 1 |
| 7 | BIRC2 | baculoviral IAP repeat containing 2 |
| 8 | BIRC3 | baculoviral IAP repeat containing 3 |
| 9 | XIAP | X-linked inhibitor of apoptosis |
| 10 | RBCK1 | RANBP2-type and C3HC4-type zinc finger containing 1 |
| 11 | RNF31 | ring finger protein 31 |
| 12 | SHARPIN | SHANK associated RH domain interactor |
| 13 | SPATA2L | spermatogenesis associated 2 like |
| 14 | SPATA2 | spermatogenesis associated 2 |
| 15 | CYLD | CYLD lysine 63 deubiquitinase |
| 16 | FADD | Fas associated via death domain |
| 17 | CASP8 | caspase 8 |
| 18 | CFLAR | CASP8 and FADD like apoptosis regulator |
| 19 | RIPK3 | receptor interacting serine/threonine kinase 3 |
| 20 | CYBB | cytochrome b-245 beta chain |
| 21 | CAMK2A | calcium/calmodulin dependent protein kinase II alpha |
| 22 | CAMK2D | calcium/calmodulin dependent protein kinase II delta |
| 23 | CAMK2B | calcium/calmodulin dependent protein kinase II beta |
| 24 | CAMK2G | calcium/calmodulin dependent protein kinase II gamma |
| 25 | SLC25A4 | solute carrier family 25 member 4 |
| 26 | SLC25A5 | solute carrier family 25 member 5 |
| 27 | SLC25A6 | solute carrier family 25 member 6 |
| 28 | SLC25A31 | solute carrier family 25 member 31 |
| 29 | PPID | peptidylprolyl isomerase D |
| 30 | VDAC1 | voltage dependent anion channel 1 |
| 31 | VDAC2 | voltage dependent anion channel 2 |
| 32 | VDAC3 | voltage dependent anion channel 3 |
| 33 | GLUD2 | glutamate dehydrogenase 2 |
| 34 | GLUD1 | glutamate dehydrogenase 1 |
| 35 | GLUL | glutamate-ammonia ligase |
| 36 | PYGL | glycogen phosphorylase L |
| 37 | PYGM | glycogen phosphorylase, muscle associated |
| 38 | PYGB | glycogen phosphorylase B |
| 39 | MAPK8 | mitogen-activated protein kinase 8 |
| 40 | MAPK10 | mitogen-activated protein kinase 10 |
| 41 | MAPK9 | mitogen-activated protein kinase 9 |
| 42 | FTH1 | ferritin heavy chain 1 |
| 43 | FTL | ferritin light chain |
| 44 | PLA2G4E | phospholipase A2 group IVE |
| 45 | PLA2G4A | phospholipase A2 group IVA |
| 46 | JMJD7-PLA2G4B | JMJD7-PLA2G4B readthrough |
| 47 | PLA2G4B | phospholipase A2 group IVB |
| 48 | PLA2G4C | phospholipase A2 group IVC |
| 49 | PLA2G4D | phospholipase A2 group IVD |
| 50 | PLA2G4F | phospholipase A2 group IVF |
| 51 | ALOX15 | arachidonate 15-lipoxygenase |
| 52 | CAPN1 | calpain 1 |
| 53 | CAPN2 | calpain 2 |
| 54 | SMPD1 | sphingomyelin phosphodiesterase 1 |
| 55 | MLKL | mixed lineage kinase domain like pseudokinase |
| 56 | PGAM5 | PGAM family member 5 |
| 57 | DNM1L | dynamin 1 like |
| 58 | NLRP3 | NLR family pyrin domain containing 3 |
| 59 | PYCARD | PYD and CARD domain containing |
| 60 | CASP1 | caspase 1 |
| 61 | IL1B | interleukin 1 beta |
| 62 | CHMP2A | charged multivesicular body protein 2A |
| 63 | CHMP2B | charged multivesicular body protein 2B |
| 64 | CHMP3 | charged multivesicular body protein 3 |
| 65 | RNF103-CHMP3 | RNF103-CHMP3 readthrough |
| 66 | CHMP4B | charged multivesicular body protein 4B |
| 67 | CHMP4A | charged multivesicular body protein 4A |
| 68 | CHMP4C | charged multivesicular body protein 4C |
| 69 | CHMP6 | charged multivesicular body protein 6 |
| 70 | VPS4B | vacuolar protein sorting 4 homolog B |
| 71 | VPS4A | vacuolar protein sorting 4 homolog A |
| 72 | CHMP1B | charged multivesicular body protein 1B |
| 73 | CHMP1A | charged multivesicular body protein 1A |
| 74 | CHMP5 | charged multivesicular body protein 5 |
| 75 | CHMP7 | charged multivesicular body protein 7 |
| 76 | TRPM7 | transient receptor potential cation channel subfamily M member 7 |
| 77 | IL1A | interleukin 1 alpha |
| 78 | IL33 | interleukin 33 |
| 79 | HMGB1 | high mobility group box 1 |
| 80 | TNFSF10 | TNF superfamily member 10 |
| 81 | TNFRSF10A | TNF receptor superfamily member 10a |
| 82 | TNFRSF10B | TNF receptor superfamily member 10b |
| 83 | FASLG | Fas ligand |
| 84 | FAS | Fas cell surface death receptor |
| 85 | FAF1 | Fas associated factor 1 |
| 86 | IFNA1 | interferon alpha 1 |
| 87 | IFNA2 | interferon alpha 2 |
| 88 | IFNA4 | interferon alpha 4 |
| 89 | IFNA5 | interferon alpha 5 |
| 90 | IFNA6 | interferon alpha 6 |
| 91 | IFNA7 | interferon alpha 7 |
| 92 | IFNA8 | interferon alpha 8 |
| 93 | IFNA10 | interferon alpha 10 |
| 94 | IFNA13 | interferon alpha 13 |
| 95 | IFNA14 | interferon alpha 14 |
| 96 | IFNA16 | interferon alpha 16 |
| 97 | IFNA17 | interferon alpha 17 |
| 98 | IFNA21 | interferon alpha 21 |
| 99 | IFNB1 | interferon beta 1 |
| 100 | IFNG | interferon gamma |
| 101 | IFNAR1 | interferon alpha and beta receptor subunit 1 |
| 102 | IFNAR2 | interferon alpha and beta receptor subunit 2 |
| 103 | IFNGR1 | interferon gamma receptor 1 |
| 104 | IFNGR2 | interferon gamma receptor 2 |
| 105 | JAK1 | Janus kinase 1 |
| 106 | JAK2 | Janus kinase 2 |
| 107 | JAK3 | Janus kinase 3 |
| 108 | TYK2 | tyrosine kinase 2 |
| 109 | STAT1 | signal transducer and activator of transcription 1 |
| 110 | STAT2 | signal transducer and activator of transcription 2 |
| 111 | STAT3 | signal transducer and activator of transcription 3 |
| 112 | STAT4 | signal transducer and activator of transcription 4 |
| 113 | STAT5A | signal transducer and activator of transcription 5A |
| 114 | STAT5B | signal transducer and activator of transcription 5B |
| 115 | STAT6 | signal transducer and activator of transcription 6 |
| 116 | IRF9 | interferon regulatory factor 9 |
| 117 | EIF2AK2 | eukaryotic translation initiation factor 2 alpha kinase 2 |
| 118 | TLR4 | toll like receptor 4 |
| 119 | TICAM2 | toll like receptor adaptor molecule 2 |
| 120 | TICAM1 | toll like receptor adaptor molecule 1 |
| 121 | TLR3 | toll like receptor 3 |
| 122 | ZBP1 | Z-DNA binding protein 1 |
| 123 | USP21 | ubiquitin specific peptidase 21 |
| 124 | SQSTM1 | sequestosome 1 |
| 125 | HSP90AA1 | heat shock protein 90 alpha family class A member 1 |
| 126 | HSP90AB1 | heat shock protein 90 alpha family class B member 1 |
| 127 | TNFAIP3 | TNF alpha induced protein 3 |
| 128 | PARP1 | poly(ADP-ribose) polymerase 1 |
| 129 | BID | BH3 interacting domain death agonist |
| 130 | BAX | BCL2 associated X, apoptosis regulator |
| 131 | AIFM1 | apoptosis inducing factor mitochondria associated 1 |
| 132 | H2AX | H2A.X variant histone |
| 133 | H2AC20 | H2A clustered histone 20 |
| 134 | H2AC12 | H2A clustered histone 12 |
| 135 | H2AC1 | H2A clustered histone 1 |
| 136 | H2AW | H2A.W histone |
| 137 | H2AB3 | H2A.B variant histone 3 |
| 138 | H2AC8 | H2A clustered histone 8 |
| 139 | H2AC4 | H2A clustered histone 4 |
| 140 | MACROH2A2 | macroH2A.2 histone |
| 141 | MACROH2A1 | macroH2A.1 histone |
| 142 | H2AC19 | H2A clustered histone 19 |
| 143 | H2AJ | H2A.J histone |
| 144 | H2AB1 | H2A.B variant histone 1 |
| 145 | H2AC17 | H2A clustered histone 17 |
| 146 | H2AC18 | H2A clustered histone 18 |
| 147 | H2AC11 | H2A clustered histone 11 |
| 148 | H2AC21 | H2A clustered histone 21 |
| 149 | H2AZ2 | H2A.Z variant histone 2 |
| 150 | H2AC7 | H2A clustered histone 7 |
| 151 | H2AZ1 | H2A.Z variant histone 1 |
| 152 | H2AC15 | H2A clustered histone 15 |
| 153 | H2AC6 | H2A clustered histone 6 |
| 154 | H2AC13 | H2A clustered histone 13 |
| 155 | H2AC14 | H2A clustered histone 14 |
| 156 | H2AC16 | H2A clustered histone 16 |
| 157 | H2AB2 | H2A.B variant histone 2 |
| 158 | PPIA | peptidylprolyl isomerase A |
| 159 | BCL2 | BCL2 apoptosis regulator |
