# Supplementary material for: Identification of hub necroptosis-related lncRNAs for prognosis prediction of esophageal carcinoma
Source: Aging (Albany NY). 2023 Jun 1;15(11):4794–819. doi: 10.18632/aging.204763 (PMC10292891; doi:10.18632/aging.204763)
Supplement: Supplementary Tables 2 and 3 [file aging-15-204763-s003.pdf]

Supplementary Table 2. The clinicopathological features of the ESCA patients.

Characteristic	Type	Training	Testing	Entire	*p*-value
Age	<=65	45(63.38%)	41(57.75%)	86(60.56%)	0.607
	>65	26(36.62%)	30(42.25%)	56(39.44%)	
Gender	FEMALE	12(16.90%)	10(14.08%)	22(15.49%)	0.817
	MALE	59(83.10%)	61(85.92%)	120(84.51%)	
Grade	G1	8(13.79%)	7(12.73%)	15(13.27%)	0.482
	G2	33(56.9%)	26(47.27%)	59(52.21%)	
	G3	17(29.31%)	22(40.00%)	39(34.51%)	
Stage	I	7(9.86%)	7(9.86%)	14(9.86%)	0.673
	II	30(42.25%)	36(50.70%)	66(46.48%)	
	III	27(38.03%)	24(33.80%)	51(35.92%)	
	IV	7(9.86%)	4(5.63%)	11(7.75%)	
T	T1	10(14.49%)	10(14.08%)	20(14.29%)	0.744
	T2	16(23.19%)	22(30.99%)	38(27.14%)	
	T3	40(57.97%)	37(52.11%)	77(55.00%)	
	T4	3(4.35%)	2(2.82%)	5(3.57%)	
M	M0	55(87.3%)	62(93.94%)	117(90.70%)	0.320
	M1	8(12.7%)	4(6.06%)	12(9.30%)	
N	N0	29(47.54%)	30(42.86%)	59(45.04%)	0.075
	N1	27(44.26%)	32(45.71%)	59(45.04%)	
	N2	3(4.92%)	5(7.14%)	8(6.11%)	
	N3	2(3.28%)	3(4.29%)	5(3.82%)	
Race	ASIAN	18(28.12%)	16(26.67%)	34(27.42%)	0.556
	WHITE	45(70.31%)	41(68.33%)	86(69.35%)	
	BLACK OR AFRICAN AMERICAN	1(1.56%)	3(5.00%)	4(3.23%)	
BMI	≤25	41(57.75%)	41(57.75%)	82(57.75%)	0.963
	>25	30(42.25%)	30(42.25%)	60(42.25%)	
New_events	Distant Metastasis	17(56.67%)	12(40%)	29(48.33%)	0.422
	Locoregional Recurrence	12(40.00%)	17(56.67%)	29(48.33%)	
	New Primary Tumor	1(3.33%)	1(3.33%)	2(3.33%)	
Radiotherapy	Local Recurrence	1(5.26%)	1(5.26%)	2(5.26%)	0.801
	Primary Tumor Field	8(42.11%)	10(52.63%)	18(47.37%)	
	Regional site	10(52.63%)	8(42.11%)	18(47.37%)	
Chemotherapy	NO	6(23.08%)	5(27.78%)	11(25.00%)	0.992
	YES	20(76.92%)	13(72.22%)	33(75.00%)	
Reflux_history	NO	33(61.11%)	38(62.3%)	71(61.74%)	0.438
	YES	21(38.89%)	23(37.7%)	44(38.26%)	

Supplementary Table 3. Primers and shRNA sequences used in this study.

**Primers**	
PVT1	**F:** 5'- TCAAGATGGCTGTGCCTGTC -3'<br>**R:** 5'- TTCCACCAGCGTTATTCCCC -3'
GAPDH	**F:** 5'-GAACGGGAAGCTTGTCATCAA-3'<br>**R:** 5'-ATCGCCCCACTTGATTTTGG-3'
**shRNA**	
shPVT1-1#	**F:**CCGGCCTGTTACACCTGGGATTTAGCTCGAGCTAAATCCCAGGTGTAACAGGTTTTTG<br>**R:**AATTCAAAAACCTGTTACACCTGGGATTTAGCTCGAGCTAAATCCCAGGTGTAACAGG
shPVT1-2#	**F:**CCGGCCGGCGCTCAGCTGGGCTTGACTCGAGTCAAGCCCAGCTGAGCGCCGGTTTTTG<br>**R:**AATTCAAAAACCGGCGCTCAGCTGGGCTTGACTCGAGTCAAGCCCAGCTGAGCGCCGG
