# Supplementary material for: Identification of hub necroptosis-related lncRNAs for prognosis prediction of esophageal carcinoma
Source: Aging (Albany NY). 2023 Jun 1;15(11):4794–819. doi: 10.18632/aging.204763 (PMC10292891; doi:10.18632/aging.204763)
Supplement: Supplementary Table 4 [file aging-15-204763-s004.docx]

**Supplementary Table 4. Differential expression of necroptosis-related genes.**

| **NRGs** | **Fold Change** | **log2FC** | ***p-value*** | **FDR** |
| --- | --- | --- | --- | --- |
| SLC25A4 | -6.075406479 | -2.602980936 | 2.39E-28 | 3.32E-26 |
| GLUL | -6.026179744 | -2.591243705 | 3.30E-23 | 2.30E-21 |
| IL33 | -5.63482474 | -2.494370739 | 1.14E-09 | 5.30E-08 |
| IFNAR1 | -1.742059198 | -0.80079365 | 1.93E-07 | 6.71E-06 |
| SMPD1 | -1.960905063 | -0.971519689 | 6.49E-07 | 1.80E-05 |
| PYGM | -5.380860306 | -2.427836853 | 2.61E-06 | 6.05E-05 |
| CAMK2D | -1.848519398 | -0.886370183 | 3.46E-06 | 6.43E-05 |
| CAPN2 | -1.726776712 | -0.788081541 | 3.70E-06 | 6.43E-05 |
| AIFM1 | -1.726984218 | -0.788254899 | 7.44E-06 | 0.000114921 |
| STAT5B | -1.682309363 | -0.75044303 | 2.67E-05 | 0.000370922 |
| RIPK1 | -1.553464006 | -0.635488814 | 4.03E-05 | 0.000509211 |
| SLC25A6 | -1.813100869 | -0.858459189 | 5.25E-05 | 0.000608232 |
| H2AX | 2.116300212 | 1.081544298 | 6.01E-05 | 0.000642358 |
| SLC25A5 | -1.585894065 | -0.665296405 | 0.000113431 | 0.001126205 |
| TNFSF10 | 3.041550989 | 1.60480719 | 0.000187116 | 0.001733946 |
| PPIA | 1.568955789 | 0.6498047 | 0.000347479 | 0.00301872 |
| PYCARD | 2.007454436 | 1.005367243 | 0.000521562 | 0.004264537 |
| CHMP3 | -1.413140135 | -0.498904538 | 0.000620603 | 0.004792431 |
| H2AC8 | 3.486595997 | 1.801819204 | 0.000722589 | 0.005286307 |
| STAT1 | 1.939891077 | 0.955975649 | 0.000970228 | 0.006743087 |
| BAX | 1.653050924 | 0.725131169 | 0.001094923 | 0.007247349 |
| VPS4A | -1.426762928 | -0.512745635 | 0.001222499 | 0.007723971 |
| H2AZ1 | 1.598933832 | 0.677110238 | 0.002611566 | 0.015782944 |
| HSP90AA1 | 1.530054945 | 0.613583462 | 0.004129509 | 0.023916738 |
| IL1A | 6.055865745 | 2.598333222 | 0.005040814 | 0.028026925 |
| FADD | 2.398003323 | 1.261833658 | 0.005278197 | 0.028218051 |
| BID | 1.639008319 | 0.712823177 | 0.006549297 | 0.033716753 |
| TNFAIP3 | 2.100365545 | 1.070640435 | 0.006880107 | 0.034154816 |
| H2AC20 | 2.741931877 | 1.455192728 | 0.00792558 | 0.037988124 |
| STAT6 | -1.308548569 | -0.387967474 | 0.010621791 | 0.0492143 |
| CFLAR | -1.443558025 | -0.529629099 | 0.01193674 | 0.053522802 |
| TLR3 | -1.790414726 | -0.840293808 | 0.012999698 | 0.054756304 |
| GLUD1 | -1.306706824 | -0.38593549 | 0.012726453 | 0.054756304 |
| JAK1 | -1.318352576 | -0.398736251 | 0.013765928 | 0.056278353 |
| MAPK9 | -1.42114246 | -0.507051182 | 0.016588407 | 0.065879672 |
| HSP90AB1 | 1.452883544 | 0.538919068 | 0.018290464 | 0.070621513 |
| TRPM7 | -1.409337805 | -0.495017454 | 0.02054786 | 0.07719331 |
| ALOX15 | 5.742493648 | 2.521677356 | 0.023003563 | 0.084144614 |
| CHMP2A | -1.32334578 | -0.404190076 | 0.024532554 | 0.08743654 |
| CAMK2G | -1.380186342 | -0.464863062 | 0.031840218 | 0.110644757 |
| PGAM5 | 1.353138792 | 0.436309825 | 0.035266392 | 0.116714965 |
| IL1B | 2.526051829 | 1.33688424 | 0.035030253 | 0.116714965 |
| TNFRSF10B | 1.479058013 | 0.56467864 | 0.038096635 | 0.123149588 |
| BCL2 | -1.778720881 | -0.830840139 | 0.04055233 | 0.125261641 |
| CHMP7 | -1.286944998 | -0.363950396 | 0.039912425 | 0.125261641 |
| CYLD | -1.364425707 | -0.448293842 | 0.041955373 | 0.126778194 |
| FTH1 | -1.34461837 | -0.427196765 | 0.047030991 | 0.136193912 |
| CASP1 | 1.69202268 | 0.758748907 | 0.046678999 | 0.136193912 |
| PARP1 | 1.321769825 | 0.402470965 | 0.054505125 | 0.151984501 |
| TNF | 2.706567619 | 1.436464432 | 0.054670684 | 0.151984501 |
| TLR4 | -1.738853064 | -0.798136028 | 0.055845648 | 0.152206766 |
| MAPK10 | -1.848271235 | -0.886176489 | 0.058840562 | 0.157285348 |
| PLA2G4F | -1.730138469 | -0.790887506 | 0.06722788 | 0.176314629 |
| H2AW | 1.601555425 | 0.679473726 | 0.071661776 | 0.18446272 |
| TRAF2 | 1.369606821 | 0.453761792 | 0.073303844 | 0.185258805 |
| TNFRSF10A | 1.388585968 | 0.473616497 | 0.077949226 | 0.193481114 |
| H2AZ2 | -1.223524739 | -0.291043272 | 0.083684581 | 0.204072926 |
| VPS4B | -1.305302741 | -0.384384452 | 0.085639011 | 0.205238319 |
| TRAF5 | -1.464784882 | -0.550688806 | 0.099071059 | 0.222487024 |
| CHMP2B | -1.279867054 | -0.355993958 | 0.100839442 | 0.222487024 |
| PPID | -1.253057623 | -0.32545276 | 0.099762117 | 0.222487024 |
| EIF2AK2 | 1.287506553 | 0.364579775 | 0.09877497 | 0.222487024 |
| H2AC17 | 1.9260426 | 0.945639613 | 0.094882208 | 0.222487024 |
| MAPK8 | -1.293857685 | -0.37167894 | 0.107277636 | 0.225933204 |
| VDAC1 | -1.200834126 | -0.264036883 | 0.105138137 | 0.225933204 |
| IFNGR2 | 1.230540247 | 0.299291844 | 0.107067744 | 0.225933204 |
| HMGB1 | 1.264928965 | 0.339056369 | 0.110332744 | 0.228899274 |
| STAT2 | 1.276804983 | 0.352538187 | 0.135449354 | 0.272861742 |
| PLA2G4E | 2.787178943 | 1.478805629 | 0.134306934 | 0.272861742 |
| PYGL | 1.77081407 | 0.824412741 | 0.149579873 | 0.29702289 |
| FAF1 | -1.199277708 | -0.262165772 | 0.162647265 | 0.318422111 |
| VDAC3 | 1.348106061 | 0.430934003 | 0.170874578 | 0.329882866 |
| CHMP1B | -1.182188566 | -0.241460172 | 0.18237162 | 0.34725555 |
| H2AC14 | 2.342456877 | 1.228022489 | 0.188230036 | 0.35356723 |
| VDAC2 | -1.193373011 | -0.255045055 | 0.199106568 | 0.364155434 |
| H2AC16 | 2.029541722 | 1.021153998 | 0.197323711 | 0.364155434 |
| RIPK3 | -1.302128424 | -0.380871743 | 0.20725438 | 0.369337933 |
| MACROH2A2 | 1.637895694 | 0.711843485 | 0.204808449 | 0.369337933 |
| H2AC11 | 1.606512596 | 0.683932292 | 0.235014571 | 0.413506651 |
| PLA2G4D | -1.752934783 | -0.809772323 | 0.245056141 | 0.425785045 |
| RBCK1 | 1.169943486 | 0.226438842 | 0.256022031 | 0.439346448 |
| STAT3 | -1.139365933 | -0.188231175 | 0.263176143 | 0.444125163 |
| H2AC18 | 2.13538382 | 1.094495407 | 0.26519704 | 0.444125163 |
| TICAM1 | -1.17618405 | -0.234113831 | 0.287717593 | 0.476104112 |
| CHMP4C | -1.233052354 | -0.302234056 | 0.299873917 | 0.490382052 |
| FAS | 1.273533168 | 0.348836534 | 0.314164879 | 0.507778119 |
| H2AC6 | 1.283148895 | 0.359688589 | 0.318522219 | 0.508903316 |
| RNF31 | -1.147691276 | -0.198734615 | 0.331505925 | 0.523628676 |
| XIAP | -1.141419456 | -0.190829059 | 0.346918072 | 0.541815865 |
| ZBP1 | 1.67385367 | 0.743173411 | 0.35298335 | 0.545163174 |
| JAK2 | -1.232669088 | -0.301785558 | 0.357050623 | 0.545385018 |
| JMJD7-PLA2G4B | -1.26275245 | -0.336571841 | 0.369186448 | 0.557792569 |
| BIRC2 | -1.112458961 | -0.153752116 | 0.376404849 | 0.562583592 |
| CHMP6 | -1.108307113 | -0.148357708 | 0.404057567 | 0.589536648 |
| CHMP4B | 1.089260187 | 0.123348606 | 0.405027558 | 0.589536648 |
| H2AC7 | 1.224598464 | 0.292308779 | 0.407162001 | 0.589536648 |
| H2AJ | -1.143154419 | -0.193020299 | 0.415294348 | 0.595112519 |
| GLUD2 | -1.25568481 | -0.328474378 | 0.426108993 | 0.604379082 |
| PLA2G4A | 1.255041091 | 0.3277346 | 0.439179615 | 0.616625924 |
| TYK2 | -1.075300859 | -0.104740369 | 0.470517991 | 0.64255309 |
| H2AC13 | 1.228503009 | 0.296901391 | 0.463245248 | 0.64255309 |
| H2AC4 | 1.71336874 | 0.776835673 | 0.471513778 | 0.64255309 |
| CAMK2B | -1.241368361 | -0.311931282 | 0.47828247 | 0.645449159 |
| JAK3 | 1.271813265 | 0.346886861 | 0.497115345 | 0.664413779 |
| H2AC12 | 1.6028113 | 0.680604586 | 0.512188839 | 0.678040463 |
| PYGB | -1.133372965 | -0.180622695 | 0.534602679 | 0.701035589 |
| TRADD | -1.073031311 | -0.101692175 | 0.549172925 | 0.713411556 |
| IFNGR1 | 1.085244625 | 0.118020277 | 0.576201905 | 0.741593193 |
| PLA2G4B | -1.242906701 | -0.313718004 | 0.611976669 | 0.766349162 |
| BIRC3 | -1.146209692 | -0.196871001 | 0.608922482 | 0.766349162 |
| CYBB | 1.198024163 | 0.260657006 | 0.603754588 | 0.766349162 |
| TNFRSF1A | -1.066208684 | -0.092489838 | 0.619883851 | 0.769320137 |
| SQSTM1 | 1.091704907 | 0.126582942 | 0.62567658 | 0.769637563 |
| CASP8 | 1.050585155 | 0.071193104 | 0.641396809 | 0.775925496 |
| H2AC19 | 1.65816556 | 0.72958806 | 0.641952749 | 0.775925496 |
| H2AB1 | 1.894144525 | 0.921546414 | 0.682865414 | 0.818261142 |
| STAT5A | -1.065703371 | -0.091805933 | 0.707140257 | 0.840106802 |
| FTL | 1.089070154 | 0.123096891 | 0.717457453 | 0.845140559 |
| MACROH2A1 | 1.032503409 | 0.046146544 | 0.740548781 | 0.865010761 |
| CHMP1A | -1.031344012 | -0.044525634 | 0.771022595 | 0.885720171 |
| IRF9 | 1.125496129 | 0.170561094 | 0.766443889 | 0.885720171 |
| CAPN1 | -1.040322941 | -0.057031444 | 0.792071043 | 0.902441598 |
| NLRP3 | -1.102968851 | -0.141392049 | 0.799382922 | 0.903367693 |
| SHARPIN | -1.018967312 | -0.027107771 | 0.818148432 | 0.917118 |
| SPATA2 | 1.039135887 | 0.055384326 | 0.859014952 | 0.955224627 |
| DNM1L | -1.017135587 | -0.024512007 | 0.877830993 | 0.968400858 |
| FASLG | -1.084698472 | -0.117294054 | 0.972253459 | 1 |
| IFNAR2 | -1.003061093 | -0.004409478 | 0.994144223 | 1 |
| CAMK2A | 1.001947614 | 0.002807081 | 0.955858587 | 1 |
| PLA2G4C | 1.002757218 | 0.003972352 | 1 | 1 |
| USP21 | 1.012434471 | 0.017828534 | 1 | 1 |
| CHMP5 | 1.014825149 | 0.021231177 | 0.996214991 | 1 |
| MLKL | 1.018209657 | 0.026034654 | 1 | 1 |
| SPATA2L | 1.022036389 | 0.031446563 | 0.930842937 | 1 |
| CHMP4A | 1.027136793 | 0.038628331 | 1 | 1 |
| RNF103-CHMP3 | 1.038616454 | 0.054662986 | 1 | 1 |
| STAT4 | 1.092535329 | 0.127679932 | 1 | 1 |
| IFNG | 1.428405855 | 0.514405952 | 0.930096065 | 1 |
| H2AC21 | 1.548184671 | 0.63057757 | 1 | 1 |
