# Supplementary material for: Identification of hub necroptosis-related lncRNAs for prognosis prediction of esophageal carcinoma
Source: Aging (Albany NY). 2023 Jun 1;15(11):4794–819. doi: 10.18632/aging.204763 (PMC10292891; doi:10.18632/aging.204763)
Supplement: Supplementary Table 5 [file aging-15-204763-s005.docx]

**Supplementary Table 5. Differential expression of necroptosis-related lncRNAs.**

| **NRLs** | **log2FC** | ***p-value*** | **FDR** |
| --- | --- | --- | --- |
| PRDM16-DT | -5.440608834 | 5.16E-19 | 1.02E-15 |
| ZNF710-AS1 | -3.3328804 | 2.62E-18 | 3.05E-15 |
| AC116407.1 | -4.592848751 | 4.51E-18 | 3.49E-15 |
| RP11-554A11.9 | -4.798587334 | 4.39E-17 | 2.55E-14 |
| AC079305.4 | -4.029759751 | 1.14E-16 | 5.28E-14 |
| C9orf147 | -3.724261206 | 5.07E-16 | 1.96E-13 |
| AC008268.1 | -4.888597805 | 1.03E-15 | 3.13E-13 |
| AC027612.2 | -4.594882445 | 1.14E-15 | 3.31E-13 |
| SEMA3B-AS1 | -2.960633676 | 3.44E-15 | 8.88E-13 |
| GATA6-AS1 | -3.740616804 | 1.47E-14 | 3.42E-12 |
| LINC02381 | -2.874827525 | 4.38E-14 | 9.24E-12 |
| AC092834.1 | -3.607501482 | 1.4E-13 | 2.71E-11 |
| H2BP1 | -3.230100932 | 2.41E-13 | 4.31E-11 |
| LINC00365 | -2.929323228 | 6.14E-13 | 1.02E-10 |
| AC091563.1 | -2.52021815 | 9.51E-13 | 1.47E-10 |
| AC129492.2 | -3.108572196 | 1.4E-12 | 2.03E-10 |
| AL158206.1 | -2.38663092 | 2.73E-12 | 3.72E-10 |
| AC244453.3 | -3.000063801 | 5.85E-12 | 7.54E-10 |
| AL049838.1 | -2.374137744 | 6.92E-12 | 8.46E-10 |
| RGMB-AS1 | -2.709278252 | 1.04E-11 | 1.21E-09 |
| PCAT18 | -3.187771302 | 2.88E-11 | 3.18E-09 |
| NCOA7-AS1 | -3.084495147 | 3.9E-11 | 4.12E-09 |
| AL512328.1 | -2.827533698 | 4.9E-11 | 4.95E-09 |
| PGM5-AS1 | -3.198157625 | 1.12E-10 | 1.09E-08 |
| AC023794.4 | -2.623490698 | 2.22E-10 | 2.06E-08 |
| FENDRR | -2.337050444 | 2.33E-10 | 2.08E-08 |
| PWAR6 | -2.529490265 | 3.65E-10 | 3.14E-08 |
| HCG21 | -3.089638695 | 8.86E-10 | 7.34E-08 |
| AC124312.2 | -2.406951707 | 1.15E-09 | 9.17E-08 |
| AC004982.1 | -2.320700371 | 1.72E-09 | 0.000000133 |
| AC053503.3 | -2.951691119 | 4.41E-09 | 0.00000033 |
| AC078906.1 | -2.644071156 | 9.29E-09 | 0.000000674 |
| AL157935.2 | -2.255336404 | 1.15E-08 | 0.000000811 |
| AC007637.1 | -2.038359488 | 1.59E-08 | 0.00000109 |
| AC015908.3 | -2.417119627 | 1.66E-08 | 0.0000011 |
| LINC02411 | -2.857491595 | 3.35E-08 | 0.00000216 |
| AC023794.5 | -2.244569055 | 6.11E-08 | 0.00000373 |
| MIR29B2CHG | -2.250005659 | 6.11E-08 | 0.00000373 |
| AC005180.2 | -2.497957159 | 6.32E-08 | 0.00000377 |
| AC004982.2 | -1.98072672 | 0.000000065 | 0.00000377 |
| SULT1C2P1 | -2.588571242 | 0.000000105 | 0.00000596 |
| ELN-AS1 | -2.129905191 | 0.00000011 | 0.00000607 |
| TMEM220-AS1 | -2.274015964 | 0.000000125 | 0.00000675 |
| AC024075.1 | -1.237557745 | 0.000000147 | 0.00000774 |
| LINC00261 | -3.189061494 | 0.000000156 | 0.00000805 |
| AP001528.1 | -2.216215125 | 0.000000195 | 0.00000984 |
| AC139491.2 | -2.438358087 | 0.000000245 | 0.0000121 |
| AL121895.2 | -1.841929092 | 0.000000299 | 0.0000145 |
| AC005920.4 | -2.65333098 | 0.000000312 | 0.0000148 |
| AC005180.1 | -2.42535699 | 0.000000325 | 0.0000151 |
| AC007193.2 | -2.377200463 | 0.000000423 | 0.0000192 |
| AC093583.1 | -2.480844909 | 0.000000479 | 0.0000214 |
| AC093010.3 | -1.281181923 | 0.000000573 | 0.0000251 |
| AC135050.6 | -0.96367261 | 0.000000604 | 0.000026 |
| AC010442.1 | -1.876228007 | 0.000000714 | 0.0000302 |
| ERVE-1 | -2.163250537 | 0.000000907 | 0.0000376 |
| AC091588.1 | -2.087233788 | 0.00000106 | 0.000043 |
| AC103563.7 | -2.779668506 | 0.00000116 | 0.0000464 |
| AF001548.1 | -2.442653627 | 0.00000191 | 0.0000751 |
| AL158847.1 | -2.363854534 | 0.00000357 | 0.000138007 |
| AC020978.4 | -1.468681472 | 0.00000394 | 0.000149991 |
| AC064807.2 | -2.145684679 | 0.00000449 | 0.00016827 |
| AC124312.3 | -2.28015546 | 0.00000512 | 0.000188682 |
| AC025280.1 | -2.259403927 | 0.00000602 | 0.000218297 |
| AC099850.4 | 1.684343064 | 0.00000612 | 0.00021872 |
| AC018926.3 | -1.711306138 | 0.0000067 | 0.000235804 |
| IDH2-DT | -2.279696304 | 0.00000723 | 0.00025072 |
| AL158163.2 | -1.877708732 | 0.00000779 | 0.000262919 |
| LINC02489 | -2.154226187 | 0.00000781 | 0.000262919 |
| AC012409.1 | -2.201582007 | 0.00000987 | 0.000327237 |
| AC084036.1 | -1.16922466 | 0.0000115 | 0.000377456 |
| BX284668.2 | -2.172503426 | 0.0000138 | 0.000446465 |
| AC108449.2 | -1.139497706 | 0.0000142 | 0.00045063 |
| SNHG14 | -1.800616063 | 0.0000151 | 0.000475313 |
| CYP2AB1P | -2.85949576 | 0.0000169 | 0.000515058 |
| AC019181.2 | -1.935536907 | 0.0000183 | 0.000550776 |
| ADAMTS9-AS1 | -2.283795547 | 0.0000216 | 0.000644282 |
| AL138930.1 | -2.238209225 | 0.0000274 | 0.000795453 |
| LINC01184 | -1.055471105 | 0.0000273 | 0.000795453 |
| AC008764.2 | -1.114377248 | 0.0000286 | 0.000812755 |
| RAB11B-AS1 | -1.166705685 | 0.0000287 | 0.000812755 |
| AL158212.3 | -1.300619731 | 0.000032 | 0.000895131 |
| AL133355.1 | -1.363508952 | 0.0000325 | 0.000897738 |
| TMEM161B-AS1 | -1.457257296 | 0.000037 | 0.001011206 |
| AC093607.1 | -2.318657657 | 0.0000382 | 0.00103017 |
| LINC01082 | -1.820760742 | 0.0000413 | 0.001102475 |
| AC024075.2 | -1.087815181 | 0.0000467 | 0.001227768 |
| AL391834.2 | -1.181657741 | 0.0000476 | 0.001227768 |
| AP003486.1 | -1.193827362 | 0.0000474 | 0.001227768 |
| TP73-AS1 | -1.244264825 | 0.0000527 | 0.001343757 |
| CYTOR | 1.883800322 | 0.0000577 | 0.001457185 |
| AC139795.1 | -1.231141225 | 0.0000599 | 0.001496514 |
| TRG-AS1 | -1.828368527 | 0.0000647 | 0.001599281 |
| AC007405.4 | -1.32496769 | 0.0000687 | 0.001679595 |
| AC027117.1 | -1.45802124 | 0.0000717 | 0.001734877 |
| AL132642.1 | -1.892486669 | 0.0000734 | 0.001756664 |
| MIR4435-2HG | 1.858730906 | 0.0000852 | 0.002019085 |
| ACTA2-AS1 | -1.640906147 | 0.0000891 | 0.002088903 |
| AC138956.2 | -1.189603412 | 0.0000907 | 0.002107061 |
| AL139275.2 | -2.52516273 | 0.0000933 | 0.002144751 |
| AC139769.1 | -1.7768738 | 0.0000994 | 0.002262169 |
| AL161431.1 | 4.181913132 | 0.000107943 | 0.002433439 |
| CHRM3-AS2 | -1.85386539 | 0.000118657 | 0.002636336 |
| RP11-701H24.5 | -1.981851794 | 0.000119214 | 0.002636336 |
| AC087477.2 | -1.93585288 | 0.000121989 | 0.002672247 |
| TSSC2 | -1.831519871 | 0.000127667 | 0.002770484 |
| AL020997.3 | -1.608329591 | 0.00013091 | 0.002814563 |
| LINC01980 | 4.052554086 | 0.000132395 | 0.002820378 |
| AC005520.4 | -1.288315912 | 0.000134512 | 0.002829965 |
| AC093797.1 | -1.851464413 | 0.000135283 | 0.002829965 |
| AC004147.4 | -2.126806845 | 0.000143693 | 0.002926794 |
| AC008808.2 | -2.132843497 | 0.000143693 | 0.002926794 |
| U62317.1 | 3.065493225 | 0.00014203 | 0.002926794 |
| AC098869.2 | -1.482398532 | 0.000145157 | 0.002930912 |
| PVT1 | 1.764777133 | 0.000149614 | 0.002969258 |
| SH3BP5-AS1 | -1.175379085 | 0.000148708 | 0.002969258 |
| AL158163.1 | -1.530715234 | 0.000151875 | 0.002988584 |
| AC130371.2 | -1.480486478 | 0.000154704 | 0.003018678 |
| AC026401.3 | 1.246914909 | 0.000156927 | 0.003036529 |
| LINC02608 | -1.691417918 | 0.000168386 | 0.003231343 |
| AL590004.3 | -1.863615497 | 0.000180519 | 0.003435783 |
| AL928768.3 | -2.07960792 | 0.000182682 | 0.003448675 |
| AC100823.1 | -1.958259484 | 0.000190577 | 0.003568715 |
| AC119424.1 | -1.98648604 | 0.000214967 | 0.003993231 |
| LINC01336 | -1.807830906 | 0.000244446 | 0.004504798 |
| AC018752.1 | -1.352237515 | 0.000249693 | 0.00453818 |
| NR2F2-AS1 | -1.955848397 | 0.000250167 | 0.00453818 |
| LINC01644 | -2.025614488 | 0.000275749 | 0.004925301 |
| AL357033.4 | -1.262236459 | 0.000281146 | 0.004983373 |
| AL035661.1 | -1.180821823 | 0.000284039 | 0.004996509 |
| AC008669.1 | -1.405998758 | 0.000286594 | 0.00500354 |
| AC007849.1 | -1.493020523 | 0.000290645 | 0.005036404 |
| LINC02777 | -1.513938139 | 0.000294616 | 0.005067387 |
| AC002546.1 | -1.90645887 | 0.000312501 | 0.005296548 |
| MIR1-1HG-AS1 | -1.929995271 | 0.000312501 | 0.005296548 |
| SNHG1 | 0.855736417 | 0.000317769 | 0.005346803 |
| AC024075.3 | -1.070261571 | 0.00034582 | 0.005776934 |
| AC103563.2 | -2.55463752 | 0.000364877 | 0.006051747 |
| DNMBP-AS1 | -1.456295663 | 0.000367858 | 0.006057923 |
| CTD-2245F17.3 | -1.441941525 | 0.000376924 | 0.0061635 |
| C8orf49 | -1.901618387 | 0.000427081 | 0.006934845 |
| COLCA1 | -1.720333583 | 0.000444664 | 0.00717021 |
| AL133370.1 | -2.017091315 | 0.000465171 | 0.007449158 |
| AC011473.3 | -1.814009141 | 0.000478082 | 0.007603466 |
| AC012181.1 | -1.335023806 | 0.000493855 | 0.007800901 |
| AL354872.2 | -1.588171533 | 0.00055159 | 0.008653994 |
| AC021016.2 | -1.380947657 | 0.000577877 | 0.009005574 |
| AL596325.2 | -1.095561654 | 0.000616257 | 0.009539659 |
| AC104825.1 | -1.164597936 | 0.000668317 | 0.01027703 |
| LINC00092 | -1.715612098 | 0.00072791 | 0.011119786 |
| CASC9 | 1.630291934 | 0.000853976 | 0.01287619 |
| AL109936.3 | -1.541465537 | 0.000871875 | 0.013061252 |
| AC012181.2 | -1.285667477 | 0.00090221 | 0.013429053 |
| SOCS2-AS1 | -1.58271921 | 0.000957137 | 0.014155873 |
| AGAP2-AS1 | 1.395722176 | 0.000979871 | 0.014400382 |
| KCNMB2-AS1 | 2.623336168 | 0.001213618 | 0.017723403 |
| AC007405.2 | -1.792652556 | 0.001262778 | 0.018180073 |
| AC092070.2 | -1.352634988 | 0.001268377 | 0.018180073 |
| LIFR-AS1 | -1.654981401 | 0.001259527 | 0.018180073 |
| AC015914.1 | -1.444610685 | 0.001299281 | 0.018508779 |
| MELTF-AS1 | 1.730891703 | 0.001388204 | 0.019654931 |
| LINC01748 | 1.759489717 | 0.001505169 | 0.021181839 |
| AC007036.2 | -1.379704151 | 0.001640733 | 0.022813069 |
| MAGOH2P | -1.284741659 | 0.001640733 | 0.022813069 |
| AC084262.1 | -1.752391441 | 0.001746384 | 0.0234399 |
| AC123912.4 | -1.649051001 | 0.001746384 | 0.0234399 |
| ADAMTS9-AS2 | -1.70073086 | 0.001746384 | 0.0234399 |
| AP001107.9 | -1.265602062 | 0.001711611 | 0.0234399 |
| RP11-380L11.4 | -1.417250331 | 0.001721384 | 0.0234399 |
| AC100830.1 | -1.609760212 | 0.001855104 | 0.024474724 |
| LINC00950 | -1.533523539 | 0.001855104 | 0.024474724 |
| AC105942.1 | -1.14620506 | 0.001894944 | 0.024719441 |
| AL157392.2 | -1.622245128 | 0.001890051 | 0.024719441 |
| AC022893.1 | -1.270959906 | 0.001998743 | 0.025927832 |
| ZNF542P | -1.197342515 | 0.002108537 | 0.027200131 |
| AC104083.1 | -1.157866869 | 0.002132964 | 0.027363211 |
| CMAHP | -1.095911493 | 0.002177523 | 0.027629553 |
| MAP4K3-DT | -1.252430325 | 0.002174606 | 0.027629553 |
| AC120498.2 | -1.589157079 | 0.002228695 | 0.028125165 |
| AC010478.1 | -1.61601547 | 0.002360905 | 0.029315619 |
| AL445426.1 | -1.677212389 | 0.002360905 | 0.029315619 |
| LINC01554 | -1.756011058 | 0.002360905 | 0.029315619 |
| HOXC-AS1 | 1.967588938 | 0.002516779 | 0.031084893 |
| AC013275.1 | -1.894527553 | 0.002539689 | 0.031201899 |
| AC103702.2 | 1.752873256 | 0.002603286 | 0.031814899 |
| GNG12-AS1 | -1.429123814 | 0.002910982 | 0.035389004 |
| LINC02657 | 3.217005388 | 0.003064549 | 0.037061886 |
| MAFG-DT | 1.682137618 | 0.003237395 | 0.038949384 |
| MIR600HG | -1.004461598 | 0.003383237 | 0.040494213 |
| BBOX1-AS1 | 1.166734995 | 0.003545499 | 0.042218716 |
| AL357033.3 | -1.148027829 | 0.003755039 | 0.04448572 |
| AC108472.1 | -1.478481253 | 0.003789504 | 0.044666139 |
| AL035701.1 | -1.426169957 | 0.003911809 | 0.045504771 |
| BX470102.1 | 1.567162291 | 0.003939043 | 0.045504771 |
| DNAH10OS | -1.423844496 | 0.003920494 | 0.045504771 |
| DUXAP8 | 1.663402774 | 0.004026845 | 0.046288785 |
| AL359715.1 | -1.293675928 | 0.004061701 | 0.046459457 |
| AC106876.1 | -1.446022914 | 0.004130634 | 0.047016335 |
| AC253536.3 | -0.997608298 | 0.004227142 | 0.047880119 |
| AC100830.2 | -1.169960787 | 0.00439511 | 0.049443798 |
| C3orf86 | -1.523510245 | 0.00440778 | 0.049443798 |
| HAND2-AS1 | -1.767428052 | 0.004502696 | 0.050082905 |
| AC011632.1 | 2.855612191 | 0.004879199 | 0.053407835 |
| LINC00491 | 2.238725916 | 0.004912996 | 0.053407835 |
| LINC01503 | 1.558995884 | 0.00508945 | 0.054711586 |
| LINC02577 | 2.532278414 | 0.005225505 | 0.055404672 |
| ROR1-AS1 | -1.538924845 | 0.005213267 | 0.055404672 |
| Z97653.1 | -1.289916246 | 0.005194637 | 0.055404672 |
| AC245041.2 | 2.00585567 | 0.005337366 | 0.056333472 |
| SNHG30 | 0.921260692 | 0.005375636 | 0.056480664 |
| MECOM-AS1 | -1.357115306 | 0.005650721 | 0.05883845 |
| AC004637.1 | -1.55923738 | 0.00570843 | 0.058959392 |
| HNF1A-AS1 | -1.628844524 | 0.005735248 | 0.058959392 |
| SNHG17 | 0.770365058 | 0.005738511 | 0.058959392 |
| U62317.2 | 1.237174346 | 0.005935117 | 0.060284663 |
| AP000866.2 | -1.170738682 | 0.006081563 | 0.060338304 |
| SLC25A21-AS1 | -1.134254676 | 0.006081563 | 0.060338304 |
| VPS9D1-AS1 | 1.089428784 | 0.00610659 | 0.060338304 |
| AC010275.1 | 2.647066541 | 0.006236191 | 0.06086578 |
| AC022031.2 | 2.321601453 | 0.006238611 | 0.06086578 |
| AP004608.1 | -1.661536546 | 0.006265437 | 0.060871731 |
| AC136475.3 | 2.06484197 | 0.006352199 | 0.061457528 |
| AP001107.5 | -1.540742146 | 0.006582089 | 0.063155412 |
| MAGI1-AS1 | -1.505122704 | 0.006575476 | 0.063155412 |
| MYOSLID | 2.591791674 | 0.006809737 | 0.064277277 |
| AC103746.1 | -1.50280574 | 0.006868874 | 0.064398642 |
| TYMSOS | 1.449890263 | 0.006878063 | 0.064398642 |
| LINC02435 | -1.621564792 | 0.006909835 | 0.064436289 |
| AC139887.4 | -1.047608914 | 0.007125434 | 0.065917362 |
| AC241952.2 | -1.0118133 | 0.007125434 | 0.065917362 |
| AL138789.1 | 2.476782794 | 0.007431531 | 0.068117997 |
| TUSC8 | -1.706429487 | 0.007480659 | 0.068117997 |
| LINC02428 | 2.192046967 | 0.007556607 | 0.068540784 |
| LINC02560 | -1.476899898 | 0.007853566 | 0.070682097 |
| ZNF300P1 | -1.341427651 | 0.007908504 | 0.07090172 |
| AC025917.1 | -1.084827172 | 0.008055266 | 0.07193972 |
| AC122129.1 | -0.981407533 | 0.008148717 | 0.072495483 |
| AC036176.1 | -0.984967149 | 0.008239613 | 0.072746701 |
| AC012085.2 | -1.599412382 | 0.008858502 | 0.076751645 |
| AC024337.1 | -1.574433176 | 0.008858502 | 0.076751645 |
| LINC01252 | -1.282619937 | 0.009069194 | 0.078285014 |
| LINC01124 | -1.321470794 | 0.009633315 | 0.082237342 |
| AC093895.2 | 1.998080224 | 0.009843894 | 0.083727185 |
| LINC02561 | 2.816157274 | 0.010060361 | 0.08525605 |
| LINC02820 | 2.288209011 | 0.010311393 | 0.086750195 |
| AC012236.1 | -1.15570925 | 0.010842666 | 0.09023896 |
| LINC00942 | 4.171334797 | 0.011012556 | 0.090677854 |
| AC109347.2 | -1.022650687 | 0.011103624 | 0.090783854 |
| TMEM51-AS1 | -1.087668677 | 0.011103624 | 0.090783854 |
| AC015908.2 | -1.606868686 | 0.011398544 | 0.092868136 |
| AL513318.2 | 1.967758561 | 0.011748468 | 0.095384419 |
| AC011472.4 | -1.032363013 | 0.0120797 | 0.095788389 |
| AL049869.3 | -1.069233805 | 0.011989533 | 0.095788389 |
| CD44-AS1 | 1.283058361 | 0.011925591 | 0.095788389 |
| FAM83A-AS1 | 2.408102892 | 0.012307091 | 0.097200902 |
| ALOX12P2 | 1.511332559 | 0.012402881 | 0.097625386 |
| AL445309.1 | -1.098537687 | 0.012579688 | 0.098682553 |
| AC104041.1 | 1.813643498 | 0.013017754 | 0.101433641 |
| AC027117.2 | -1.091581837 | 0.013144476 | 0.102078503 |
| HCP5 | 0.901340631 | 0.013298223 | 0.102586292 |
| TSTD3 | -0.830484222 | 0.013296626 | 0.102586292 |
| RPLP0P2 | 1.559825563 | 0.013407188 | 0.103084404 |
| ARHGEF2-AS1 | -1.344241433 | 0.013514564 | 0.103567059 |
| AC008770.3 | -1.118504293 | 0.013911664 | 0.104624802 |
| TTC39A-AS1 | -1.321120428 | 0.013922939 | 0.104624802 |
| AC022075.1 | 1.676711861 | 0.014131467 | 0.105849248 |
| HOXD-AS2 | 1.986680959 | 0.014200562 | 0.105894577 |
| LINC02701 | -1.346561972 | 0.014290164 | 0.106012019 |
| AL356124.2 | -1.505866475 | 0.014801996 | 0.108766566 |
| AC019197.1 | -1.274524944 | 0.015777298 | 0.114484017 |
| AL390208.1 | -1.07524144 | 0.015748494 | 0.114484017 |
| AC011379.1 | -1.336241969 | 0.016002681 | 0.115757708 |
| AC025575.2 | 1.462465674 | 0.016327461 | 0.117013468 |
| LINC02593 | -1.305710108 | 0.016494023 | 0.117843447 |
| SCAT1 | 1.833219041 | 0.016965638 | 0.120841138 |
| SNHG15 | 0.737633982 | 0.017264349 | 0.122592714 |
| TMPO-AS1 | 1.207651448 | 0.017998332 | 0.126642807 |
| KRT8P12 | 0.895201187 | 0.018103764 | 0.126677788 |
| LINC02595 | 1.113461302 | 0.018462162 | 0.128350721 |
| AC007991.2 | 2.360980358 | 0.018644844 | 0.128360901 |
| AC012485.2 | -1.227711059 | 0.018758272 | 0.128360901 |
| AC018926.1 | -1.168461901 | 0.018795308 | 0.128360901 |
| AP001189.1 | -1.327145569 | 0.018558613 | 0.128360901 |
| AL109741.1 | -1.192766095 | 0.01918504 | 0.130256323 |
| LINC01615 | 2.469875356 | 0.019551204 | 0.132355378 |
| CA3-AS1 | -1.117578703 | 0.019741362 | 0.132867948 |
| AC139768.1 | -0.967615733 | 0.020291402 | 0.136175248 |
| AP001094.1 | -1.287001026 | 0.021400491 | 0.141572481 |
| LINC00519 | 2.386340104 | 0.021905932 | 0.143998729 |
| AC012557.1 | -0.984801782 | 0.023715057 | 0.153388194 |
| FAM230C | -1.428971019 | 0.024118827 | 0.155566431 |
| AC019117.2 | 2.087214352 | 0.02445028 | 0.155923678 |
| LINC01018 | -1.088808234 | 0.024545192 | 0.155923678 |
| LINC02185 | -1.389940358 | 0.024577117 | 0.155923678 |
| FAM13A-AS1 | -1.127076125 | 0.02478136 | 0.156791056 |
| NKILA | 1.783153729 | 0.025053054 | 0.158079324 |
| HMGB1P5 | 0.738659068 | 0.025891989 | 0.160751866 |
| AC009093.6 | -1.204291213 | 0.026132818 | 0.160966386 |
| AC087491.1 | 2.67778222 | 0.026493293 | 0.16231511 |
| RP11-539I5.1 | -0.934122282 | 0.026890875 | 0.163457099 |
| AL049555.1 | 0.894782236 | 0.02712469 | 0.164019613 |
| U62317.4 | 1.997477756 | 0.027560028 | 0.165788564 |
| AC073842.1 | -1.317056253 | 0.028654182 | 0.171555569 |
| AL365181.3 | 1.395515431 | 0.028947698 | 0.172793202 |
| AC112236.2 | -1.163678619 | 0.029307446 | 0.174045754 |
| AC107072.2 | -1.060635608 | 0.029649767 | 0.175629487 |
| PSMB8-AS1 | 0.792376218 | 0.029925358 | 0.176463776 |
| SMIM25 | 1.261112511 | 0.030021187 | 0.176478975 |
| AL121832.2 | 0.924265767 | 0.030612258 | 0.179499151 |
| AC015660.1 | 1.769617184 | 0.030936511 | 0.18048889 |
| AC015912.3 | 0.917258951 | 0.030870054 | 0.18048889 |
| AC113346.1 | 2.188679212 | 0.03101509 | 0.180493835 |
| AC018755.3 | -0.974714668 | 0.032351242 | 0.186446862 |
| AL513008.1 | -0.861673534 | 0.03235921 | 0.186446862 |
| AC010136.1 | -1.08126325 | 0.033178599 | 0.190223968 |
| AP003059.1 | -1.117773581 | 0.033178599 | 0.190223968 |
| AL691482.4 | 1.361052321 | 0.03328953 | 0.190389873 |
| AC016590.2 | -0.86572652 | 0.033727469 | 0.192420598 |
| AC138956.1 | -0.883979142 | 0.034288159 | 0.195139966 |
| AC095057.3 | -0.908629267 | 0.034588242 | 0.196366501 |
| LINC02303 | -1.244796532 | 0.034710041 | 0.196577356 |
| GK-IT1 | 1.675452055 | 0.034950796 | 0.197459239 |
| RP11-298C2.1 | -1.028490685 | 0.035669176 | 0.200541951 |
| AL157392.3 | -0.853403511 | 0.035859527 | 0.201125174 |
| DIRC3 | -1.15233851 | 0.037916722 | 0.209805662 |
| AL031768.1 | -1.171272439 | 0.038171442 | 0.210033383 |
| AC017060.1 | 2.083075615 | 0.039230185 | 0.214335267 |
| AC006116.11 | -1.23133667 | 0.03989865 | 0.215452708 |
| AC008011.2 | 2.200591784 | 0.03971695 | 0.215452708 |
| AC093673.1 | 0.799136955 | 0.039688598 | 0.215452708 |
| LINC01992 | -1.205846163 | 0.04019968 | 0.216574611 |
| AL590666.2 | 1.228189351 | 0.040422552 | 0.217271219 |
| LINC00707 | 2.381086407 | 0.041594815 | 0.222541845 |
| DLEU2 | 1.024281114 | 0.042032795 | 0.224368163 |
| AC004803.1 | -0.849294847 | 0.042674077 | 0.226231065 |
| AC026369.2 | 1.612045505 | 0.043202757 | 0.227992731 |
| AC053513.2 | -1.001627853 | 0.043708458 | 0.228781154 |
| C2orf48 | 1.592902707 | 0.043746267 | 0.228781154 |
| RP13-514E23.1 | -1.072583392 | 0.0445425 | 0.231381843 |
| ZNF582-AS1 | -1.117644167 | 0.0445425 | 0.231381843 |
| AL359513.1 | 1.28081357 | 0.044877743 | 0.232602943 |
| AC103591.3 | 1.19402017 | 0.045656663 | 0.235224291 |
| GBP1P1 | 1.117329907 | 0.045687405 | 0.235224291 |
| TM4SF19-AS1 | 1.690037691 | 0.046365959 | 0.238189728 |
| DLGAP1-AS2 | 0.987016094 | 0.04658477 | 0.238785511 |
| AC006942.1 | -1.044770266 | 0.047101135 | 0.240900519 |
| TREML3P | 2.445022083 | 0.048245235 | 0.244597023 |
| TUBBP5 | 1.22110079 | 0.050019194 | 0.252488193 |
| AC003965.2 | 1.332570289 | 0.050779526 | 0.254963635 |
| AC002451.1 | -1.191801418 | 0.051874501 | 0.256636157 |
| CTA-292E10.9 | -0.982984296 | 0.051946164 | 0.256636157 |
| ZBED3-AS1 | -1.090719754 | 0.051874501 | 0.256636157 |
| AC010280.1 | 2.464336777 | 0.053263992 | 0.261477778 |
| HOXB-AS4 | 1.037499951 | 0.053445204 | 0.261813848 |
| GSN-AS1 | -1.055015619 | 0.054436117 | 0.26610666 |
| AC023157.3 | 0.888357588 | 0.055155725 | 0.26905797 |
| AL391056.1 | 1.785663715 | 0.055766497 | 0.271467101 |
| LOH12CR2 | -0.876161628 | 0.056038826 | 0.271653765 |
| AC010226.1 | -0.938408247 | 0.056192769 | 0.271832519 |
| AP003390.1 | 1.482290977 | 0.057013543 | 0.274311659 |
| AC073585.1 | 1.413683105 | 0.057595992 | 0.276317962 |
| AC020779.2 | -1.406668112 | 0.058732255 | 0.278193449 |
| AC129492.4 | -1.212348584 | 0.058732255 | 0.278193449 |
| AP005717.1 | -1.032311023 | 0.058732255 | 0.278193449 |
| ZNF793-AS1 | -0.993749852 | 0.059737533 | 0.281070996 |
| AL118516.1 | 0.743944093 | 0.060913533 | 0.284018523 |
| AC106798.1 | 1.487632911 | 0.062281202 | 0.288081575 |
| AC010300.1 | -0.944550452 | 0.062472263 | 0.288390843 |
| LINC00888 | 1.008275557 | 0.063551504 | 0.292211074 |
| AC091057.1 | 0.901819251 | 0.064861802 | 0.297244368 |
| GMDS-DT | -1.002556125 | 0.065382677 | 0.297244368 |
| RP11-495P10.8 | 1.683375754 | 0.065123912 | 0.297244368 |
| AL683887.1 | 2.038090193 | 0.066083496 | 0.299114773 |
| DRAIC | -1.234099974 | 0.067492592 | 0.303937619 |
| LUCAT1 | 1.674367481 | 0.067405922 | 0.303937619 |
| AC015921.1 | -1.045551698 | 0.068737683 | 0.306351056 |
| LINC02195 | 1.015750983 | 0.068410674 | 0.306351056 |
| AC007991.4 | 1.599638981 | 0.069389593 | 0.306436168 |
| AC090912.3 | 1.482230637 | 0.069696469 | 0.306436168 |
| AC135178.2 | -1.254356394 | 0.069812546 | 0.306436168 |
| AP001094.2 | -1.083882646 | 0.069812546 | 0.306436168 |
| BVES-AS1 | -1.086158251 | 0.069812546 | 0.306436168 |
| CYP4F35P | -1.202477303 | 0.06917127 | 0.306436168 |
| LINC02323 | 1.188721992 | 0.069400771 | 0.306436168 |
| AC010186.2 | 1.03614274 | 0.070738684 | 0.309915518 |
| MAGEA4-AS1 | 1.894128053 | 0.073027416 | 0.318739964 |
| CTC-428H11.2 | -0.821292452 | 0.074691278 | 0.324781176 |
| LINC02615 | -0.953559573 | 0.075645675 | 0.327703837 |
| AL365181.2 | 1.349112572 | 0.076670821 | 0.330910122 |
| AC012618.3 | -0.987942712 | 0.077736878 | 0.333035114 |
| AC020659.1 | -0.996135644 | 0.078300197 | 0.334215179 |
| HOTAIR | 1.388538781 | 0.079044752 | 0.336157353 |
| AL136018.1 | 1.766472506 | 0.0798838 | 0.338157512 |
| LINC01833 | 1.255444255 | 0.079676428 | 0.338157512 |
| AC010329.1 | -1.182928617 | 0.08201855 | 0.343147881 |
| AC011816.2 | -1.052451278 | 0.08201855 | 0.343147881 |
| AC051619.4 | -1.190787178 | 0.08201855 | 0.343147881 |
| AC138649.1 | -0.920681165 | 0.08201855 | 0.343147881 |
| TSPEAR-AS2 | 1.436967867 | 0.081287978 | 0.343147881 |
| AL022067.1 | 1.185764664 | 0.083092936 | 0.345501031 |
| LINC00885 | 1.559445645 | 0.083176174 | 0.345501031 |
| MIR3936HG | -0.775377326 | 0.084383957 | 0.349268356 |
| AL035461.3 | 0.865293844 | 0.08535115 | 0.352643008 |
| LINC02677 | 2.041380702 | 0.08555953 | 0.352876072 |
| AC087854.1 | -1.038932114 | 0.087695134 | 0.360145784 |
| MIR222HG | 0.843224974 | 0.087787474 | 0.360145784 |
| AC004594.1 | -0.895785941 | 0.088631558 | 0.36105698 |
| AL355076.2 | -1.11158493 | 0.088631558 | 0.36105698 |
| SGMS1-AS1 | -0.834001539 | 0.089828003 | 0.36465144 |
| LINC02688 | -1.197197668 | 0.090741004 | 0.367074236 |
| C5orf34-AS1 | 1.227762954 | 0.092237722 | 0.369906718 |
| AC106795.1 | 0.79085639 | 0.093107676 | 0.372751765 |
| AC009271.1 | 1.64504057 | 0.093872779 | 0.373665954 |
| AC011444.3 | -1.158736883 | 0.095352604 | 0.373665954 |
| AL021807.1 | 1.000881805 | 0.0942605 | 0.373665954 |
| AL356124.1 | -0.97394602 | 0.095352604 | 0.373665954 |
| LINC01410 | 1.18345359 | 0.095154501 | 0.373665954 |
| TUG1 | 0.260541794 | 0.095428041 | 0.373665954 |
| AL356599.1 | -0.945739867 | 0.097009535 | 0.376053658 |
| SUCLG2-AS1 | -1.040812557 | 0.097009535 | 0.376053658 |
| AC006305.2 | 1.561482346 | 0.098709409 | 0.381369798 |
| AC016738.1 | -0.949603497 | 0.099648261 | 0.382256188 |
| CEBPB-AS1 | -0.848146056 | 0.099648261 | 0.382256188 |
| LINC01508 | 1.663982571 | 0.101341985 | 0.38703304 |
| LNCTAM34A | 1.070473369 | 0.101706635 | 0.38778786 |
| AC058791.1 | -0.78316099 | 0.10358059 | 0.392996943 |
| FOXD2-AS1 | 0.611041906 | 0.103556159 | 0.392996943 |
| AL158166.1 | 1.144675902 | 0.103869431 | 0.393449951 |
| PIK3CD-AS2 | 1.019793629 | 0.105992443 | 0.400837872 |
| CHMP1B2P | -1.030244913 | 0.106892683 | 0.401040509 |
| PSMD6-AS2 | -0.967461074 | 0.106892683 | 0.401040509 |
| FAR2P4 | 2.222328936 | 0.108586044 | 0.406672248 |
| AC099487.1 | 1.307883747 | 0.109984801 | 0.407312136 |
| AL121957.1 | -0.950159428 | 0.109809459 | 0.407312136 |
| AL591767.1 | -1.125488577 | 0.109809459 | 0.407312136 |
| C21orf62-AS1 | -0.914146792 | 0.109809459 | 0.407312136 |
| RP11-527D7.1 | -1.047979895 | 0.109458025 | 0.407312136 |
| LINC02541 | 1.122058938 | 0.110368121 | 0.408080853 |
| AC010210.1 | -0.850673323 | 0.111624173 | 0.411414809 |
| AP000866.1 | -1.034900068 | 0.111624173 | 0.411414809 |
| AC034213.1 | 1.378171761 | 0.112067184 | 0.412393028 |
| SEMA3F-AS1 | -0.830841195 | 0.11279975 | 0.414431994 |
| LINC00393 | 2.155899858 | 0.11415957 | 0.418765436 |
| AC015727.1 | -0.841689428 | 0.117736541 | 0.426496486 |
| AC063950.1 | -0.88767335 | 0.117736541 | 0.426496486 |
| AL132657.1 | -0.95660358 | 0.11739918 | 0.426496486 |
| AL139383.1 | -0.868132056 | 0.11739918 | 0.426496486 |
| L3MBTL4-AS1 | -0.840905159 | 0.11739918 | 0.426496486 |
| LINC02562 | 0.691226902 | 0.117124684 | 0.426496486 |
| SNHG4 | 0.600166836 | 0.116828522 | 0.426496486 |
| KMT2E-AS1 | 0.568988022 | 0.118138607 | 0.427286363 |
| AC009005.1 | 1.056392931 | 0.120291506 | 0.43304942 |
| RP11-757O6.1 | -0.878003872 | 0.122254608 | 0.438756104 |
| PCAT6 | 0.834894198 | 0.123165732 | 0.441343873 |
| AL031846.2 | -0.870755201 | 0.124288437 | 0.443314517 |
| FAM66D | -0.791526224 | 0.124288437 | 0.443314517 |
| AC007128.2 | 1.367985298 | 0.126309535 | 0.447772124 |
| CLEC4O | -1.086292063 | 0.130062286 | 0.45758277 |
| CXCR2P1 | 1.255460664 | 0.132700893 | 0.464053424 |
| AL121655.1 | -0.729326197 | 0.133977779 | 0.467111717 |
| CHKB-DT | 0.925704711 | 0.134232814 | 0.46729924 |
| AP000695.1 | 1.076851689 | 0.134443853 | 0.467333275 |
| AC009126.1 | -0.689150697 | 0.135035178 | 0.467987587 |
| AC128709.2 | 1.49680278 | 0.136886875 | 0.472993041 |
| AL162171.2 | -0.924818657 | 0.137727772 | 0.474486477 |
| ARMC2-AS1 | -0.86845206 | 0.137727772 | 0.474486477 |
| AC012557.2 | -0.967516748 | 0.140153015 | 0.480702069 |
| AC114980.1 | -0.75276521 | 0.140153015 | 0.480702069 |
| AC004816.1 | -0.605839597 | 0.141758893 | 0.481502878 |
| AC009554.1 | -0.983017516 | 0.14203588 | 0.481502878 |
| AL022315.1 | -1.010832065 | 0.14203588 | 0.481502878 |
| AL590226.1 | -0.789586357 | 0.14203588 | 0.481502878 |
| LINC01518 | 2.000362118 | 0.142045423 | 0.481502878 |
| AC006206.2 | 1.263562491 | 0.143435548 | 0.484799624 |
| Z83851.4 | 0.543818601 | 0.144173019 | 0.485173552 |
| LINC01943 | 1.544228174 | 0.145393689 | 0.488573293 |
| AP002478.1 | 1.461850849 | 0.146370811 | 0.491145987 |
| MT1L | -0.78400805 | 0.146932525 | 0.491609977 |
| AP005262.2 | 1.756817533 | 0.148079171 | 0.494022752 |
| MANCR | 1.609543689 | 0.14790458 | 0.494022752 |
| AL355802.3 | 1.012619458 | 0.14943706 | 0.497124432 |
| AC112496.1 | 1.220942505 | 0.149840388 | 0.497233827 |
| AL772337.3 | 1.769729079 | 0.15029271 | 0.497831202 |
| LINC00663 | -0.910583656 | 0.152415664 | 0.501287779 |
| C8orf31 | 1.280850939 | 0.154379855 | 0.507029736 |
| LINC01419 | 2.409688899 | 0.155367922 | 0.509554119 |
| LINC00958 | 1.180962195 | 0.157147548 | 0.51466376 |
| AC114956.1 | 1.074672884 | 0.158511838 | 0.515232284 |
| CETN4P | -0.866796831 | 0.159761949 | 0.515232284 |
| RBM5-AS1 | -0.705760543 | 0.159597372 | 0.515232284 |
| RP11-160O5.1 | 0.678181443 | 0.159393198 | 0.515232284 |
| SCOC-AS1 | -0.878934544 | 0.159761949 | 0.515232284 |
| TRPM2-AS | 0.550559188 | 0.159381105 | 0.515232284 |
| AC147651.4 | 1.056729336 | 0.161498341 | 0.519389402 |
| AC087442.1 | 1.466338176 | 0.16177228 | 0.519550809 |
| AP001267.3 | -0.780134652 | 0.162281482 | 0.5204663 |
| AC010201.2 | -0.778062449 | 0.165264721 | 0.527873124 |
| AL021368.2 | -0.755907448 | 0.165264721 | 0.527873124 |
| AC010201.1 | -0.90111498 | 0.166901961 | 0.530159171 |
| LINC01545 | -0.749374641 | 0.166901961 | 0.530159171 |
| MIRLET7A1HG | -0.911308646 | 0.166901961 | 0.530159171 |
| MAP3K20-AS1 | 1.268022018 | 0.168003746 | 0.532202862 |
| RP9P | 0.466211586 | 0.169419467 | 0.535956408 |
| AC090970.2 | 1.118109851 | 0.170327508 | 0.537905763 |
| AP002449.1 | 0.871897524 | 0.171842701 | 0.539944182 |
| AC004585.1 | 1.528980592 | 0.172515027 | 0.541083045 |
| TUBBP1 | 0.645964548 | 0.173062475 | 0.541578256 |
| AC012363.2 | 0.708895193 | 0.17875241 | 0.549210592 |
| AC024941.1 | -0.843604024 | 0.179285801 | 0.549210592 |
| AC073957.1 | 1.602003311 | 0.17896545 | 0.549210592 |
| AC079322.1 | -0.686427063 | 0.17869494 | 0.549210592 |
| AF131215.4 | -0.714989193 | 0.178524983 | 0.549210592 |
| AP002784.1 | 1.381507012 | 0.177684154 | 0.549210592 |
| LYPLAL1-DT | -0.848759873 | 0.179285801 | 0.549210592 |
| RP11-932O9.10 | 1.057101151 | 0.176982208 | 0.549210592 |
| RPSAP36 | -0.7546234 | 0.17869494 | 0.549210592 |
| AC010203.2 | -0.750055722 | 0.182614586 | 0.557202456 |
| RPSAP52 | 1.398276294 | 0.183374244 | 0.558786081 |
| LINC02454 | 1.588944842 | 0.185430832 | 0.561839135 |
| RHPN1-AS1 | 0.984266344 | 0.185582172 | 0.561839135 |
| Z98257.1 | 1.312195783 | 0.185585968 | 0.561839135 |
| AC116049.2 | 1.042711102 | 0.187263348 | 0.565442776 |
| H2AC10P | 1.010355797 | 0.187263348 | 0.565442776 |
| AC009118.2 | 1.415126642 | 0.188453292 | 0.566824538 |
| LINC01010 | 1.297947595 | 0.188382947 | 0.566824538 |
| TMEM44-AS1 | 0.496241529 | 0.192073123 | 0.576219369 |
| LINC01816 | 0.784627409 | 0.196065574 | 0.585925692 |
| GK-AS1 | 1.188228382 | 0.198588586 | 0.587310092 |
| LINC01697 | -0.814673265 | 0.199058158 | 0.587310092 |
| MMP25-AS1 | 0.644666296 | 0.198789311 | 0.587310092 |
| SIGLEC16 | -0.906415413 | 0.19829019 | 0.587310092 |
| AP000487.1 | 0.881818044 | 0.200390311 | 0.589741828 |
| CTD-2521M24.9 | 0.490012989 | 0.201240982 | 0.590253806 |
| MYO16-AS1 | 1.257504725 | 0.201326879 | 0.590253806 |
| AL021408.1 | -0.93095713 | 0.205389508 | 0.593915863 |
| LINC02474 | 2.016104128 | 0.204865227 | 0.593915863 |
| TNFRSF10A-AS1 | 0.440132506 | 0.207736957 | 0.598166764 |
| AC091729.3 | 0.624326752 | 0.209749663 | 0.600299808 |
| AC092969.1 | 1.74584712 | 0.209113092 | 0.600299808 |
| AL023803.1 | 1.04100149 | 0.209923964 | 0.600299808 |
| AC004264.1 | 1.116511048 | 0.210638941 | 0.60160347 |
| AC016065.1 | 0.685220223 | 0.214122492 | 0.609421869 |
| AL135818.2 | 1.336436969 | 0.214163757 | 0.609421869 |
| LINC01633 | 1.210433518 | 0.214454192 | 0.609501388 |
| AC124319.1 | 0.736962627 | 0.215096404 | 0.61057928 |
| MAN1B1-DT | 0.705933399 | 0.215466302 | 0.610882483 |
| FZD4-DT | -0.73104778 | 0.21621495 | 0.61151171 |
| AC116407.2 | 0.859667367 | 0.216938124 | 0.612242215 |
| EIF1B-AS1 | -0.875544927 | 0.219018591 | 0.613463412 |
| RP11-499F3.1 | 1.225235217 | 0.218980861 | 0.613463412 |
| MIR924HG | 1.147132658 | 0.219355711 | 0.613667422 |
| PTMAP5 | 0.360786271 | 0.220367895 | 0.615757223 |
| LINC00839 | 1.030397957 | 0.221999641 | 0.618778832 |
| SNHG26 | 0.682023953 | 0.222213289 | 0.618778832 |
| AL133353.1 | 0.934870685 | 0.227682897 | 0.63085895 |
| LINC02535 | 0.999217591 | 0.229065087 | 0.633201349 |
| AC007277.1 | -0.726662995 | 0.232887743 | 0.634701102 |
| AC012404.2 | -0.813590455 | 0.232887743 | 0.634701102 |
| AC021205.3 | -0.908127878 | 0.232887743 | 0.634701102 |
| AC024581.1 | 1.415711609 | 0.230620142 | 0.634701102 |
| AC091544.2 | -0.774383646 | 0.232887743 | 0.634701102 |
| AC119150.1 | 1.428847242 | 0.230620142 | 0.634701102 |
| AL158068.2 | -0.769984305 | 0.232887743 | 0.634701102 |
| LNX1-AS2 | -0.667260333 | 0.232887743 | 0.634701102 |
| AP000251.1 | 1.227568944 | 0.234636131 | 0.638716408 |
| LINC01564 | 0.969152138 | 0.23656996 | 0.642474208 |
| AL160314.2 | -0.832482589 | 0.240667575 | 0.650558916 |
| AC002076.1 | 1.575801667 | 0.242831218 | 0.654123072 |
| AC091182.2 | 0.984356857 | 0.243172816 | 0.654284217 |
| AC087588.2 | 0.816742292 | 0.244872675 | 0.658095315 |
| AC100861.1 | 0.621864959 | 0.245937861 | 0.6593576 |
| LINC01932 | 1.116375456 | 0.245760842 | 0.6593576 |
| SNHG19 | 0.385683551 | 0.246194246 | 0.6593576 |
| AP002336.2 | 0.779327263 | 0.249369422 | 0.665558388 |
| AP003119.2 | 0.885708205 | 0.249882057 | 0.66616089 |
| AC008083.2 | 1.353998617 | 0.250865576 | 0.666487263 |
| LINC01605 | 0.893812452 | 0.254354192 | 0.672909565 |
| AC012640.4 | 0.773693622 | 0.254788793 | 0.673059815 |
| AC002401.4 | 1.204832231 | 0.257209422 | 0.673729314 |
| AC005899.8 | -0.625093189 | 0.258093692 | 0.673729314 |
| AC021739.2 | -0.667899551 | 0.258093692 | 0.673729314 |
| AL353764.1 | 1.324768026 | 0.255638114 | 0.673729314 |
| FAM157A | 0.866965947 | 0.257039091 | 0.673729314 |
| MIR1915HG | 1.099428795 | 0.256671395 | 0.673729314 |
| AC010261.1 | -0.615963309 | 0.261665719 | 0.677355406 |
| ANKRD20A21P | -0.602887107 | 0.261665719 | 0.677355406 |
| LINC01456 | 1.048911451 | 0.26113552 | 0.677355406 |
| AC093159.1 | 1.364460243 | 0.262360954 | 0.677992219 |
| NCK1-DT | 0.525451628 | 0.264464898 | 0.682319437 |
| AC018653.3 | 0.483541763 | 0.269794368 | 0.693756946 |
| AL138724.2 | 0.590222644 | 0.269343603 | 0.693756946 |
| AC022211.3 | 0.894432589 | 0.270656908 | 0.694855274 |
| AC245041.1 | 0.82404922 | 0.270819993 | 0.694855274 |
| AC104958.2 | -0.604722012 | 0.27136822 | 0.695493384 |
| LINC00944 | 0.966906878 | 0.274181138 | 0.700383501 |
| Z94721.1 | 0.505440069 | 0.275819733 | 0.703022414 |
| AC131097.2 | 1.239508211 | 0.276435605 | 0.703819599 |
| AC012368.1 | 0.942893675 | 0.276944696 | 0.704343465 |
| AC008556.1 | 0.710049759 | 0.279226815 | 0.707109055 |
| AC009902.2 | 0.797383937 | 0.279339842 | 0.707109055 |
| RPL30P11 | 0.999983078 | 0.279554743 | 0.707109055 |
| LINC01068 | 1.179940801 | 0.282306982 | 0.712518273 |
| AC083906.3 | 0.978116185 | 0.283138575 | 0.713841229 |
| AC128709.3 | 1.245647413 | 0.283584136 | 0.714189115 |
| HID1-AS1 | -0.784693983 | 0.286541851 | 0.720075949 |
| AL033384.1 | 1.027602766 | 0.288224873 | 0.723522331 |
| AC092134.1 | -0.569751015 | 0.291606914 | 0.723946268 |
| AL161891.1 | 0.855536196 | 0.289669115 | 0.723946268 |
| AL359853.1 | -0.55078179 | 0.291823302 | 0.723946268 |
| AP000879.2 | 1.234885873 | 0.291367502 | 0.723946268 |
| ATP2A1-AS1 | 0.903495906 | 0.289217228 | 0.723946268 |
| C2orf27A | 0.625830915 | 0.289231008 | 0.723946268 |
| KIF9-AS1 | -0.738565286 | 0.291542923 | 0.723946268 |
| AC083973.1 | 0.89461953 | 0.292546567 | 0.724965986 |
| AL031848.1 | 1.046214668 | 0.294258195 | 0.727654451 |
| AC099518.6 | 0.987967534 | 0.294697411 | 0.727965308 |
| AL031667.3 | 0.905198461 | 0.296792169 | 0.732360698 |
| MIR205HG | 1.059128212 | 0.300810392 | 0.739917087 |
| AC004080.2 | 0.773066475 | 0.30598693 | 0.751058829 |
| SNHG10 | 0.560560099 | 0.307119589 | 0.753042963 |
| LINC01235 | 0.848762315 | 0.307496266 | 0.753171233 |
| AP005137.2 | -0.945900429 | 0.310668427 | 0.756155227 |
| H2BC20P | 0.516456142 | 0.309377025 | 0.756155227 |
| LINC00202-1 | -0.600025033 | 0.310668427 | 0.756155227 |
| LINC02241 | 1.070387349 | 0.309528492 | 0.756155227 |
| MFF-DT | -0.755490215 | 0.310668427 | 0.756155227 |
| CLCA4-AS1 | -0.551968465 | 0.311925753 | 0.756835526 |
| HPN-AS1 | -0.724356442 | 0.311925753 | 0.756835526 |
| RP11-254F7.4 | 1.115213383 | 0.312398695 | 0.757191826 |
| LINC01705 | 1.059360929 | 0.313896348 | 0.760028487 |
| SLC47A1P2 | 1.168775591 | 0.319173222 | 0.76872337 |
| AC007848.1 | 0.972789583 | 0.322752352 | 0.772609239 |
| AC008676.1 | -0.827777538 | 0.322594436 | 0.772609239 |
| AC016683.1 | -0.886104356 | 0.322594436 | 0.772609239 |
| AC010186.3 | 0.653496607 | 0.326043146 | 0.774389673 |
| AC082651.3 | 0.971666571 | 0.326130177 | 0.774389673 |
| AL135924.2 | 0.917514997 | 0.324512503 | 0.774389673 |
| KDM7A-DT | 0.438936911 | 0.323981641 | 0.774389673 |
| AC026356.1 | 0.513083957 | 0.328508679 | 0.777571002 |
| RP11-199F11.2 | 0.523663467 | 0.328508679 | 0.777571002 |
| AC025244.1 | 1.094633104 | 0.329493056 | 0.778314219 |
| AC011352.3 | 1.113264423 | 0.333004198 | 0.785808688 |
| MKNK1-AS1 | -0.67847904 | 0.335519092 | 0.790939423 |
| LINC00920 | 0.642408103 | 0.336638316 | 0.791138139 |
| CNOT2-DT | 0.787716895 | 0.338512591 | 0.792845863 |
| KRT17P6 | 0.76018493 | 0.339742306 | 0.792845863 |
| AL109976.1 | 0.756788064 | 0.343818822 | 0.79914645 |
| AC046143.1 | 0.595265787 | 0.344445609 | 0.799802703 |
| AC139720.1 | 0.63074331 | 0.34641236 | 0.800990478 |
| SNHG32 | 0.24086185 | 0.347376464 | 0.801797365 |
| DLGAP1-AS1 | 0.388584733 | 0.350435332 | 0.807252818 |
| CYP4A22-AS1 | 0.903755287 | 0.350964902 | 0.80767146 |
| AC040169.1 | 0.566692008 | 0.351749744 | 0.808676145 |
| AC008687.3 | 1.256853276 | 0.355163086 | 0.809311762 |
| AC074117.1 | 0.323137668 | 0.354914326 | 0.809311762 |
| AC099518.1 | 1.079818325 | 0.355163086 | 0.809311762 |
| ETV7-AS1 | 0.924306814 | 0.355163086 | 0.809311762 |
| MAGI1-IT1 | -0.7871345 | 0.354512183 | 0.809311762 |
| KRT17P1 | 0.806394993 | 0.356264678 | 0.810231715 |
| AC022973.5 | 0.816640186 | 0.364440098 | 0.815250394 |
| AC024230.1 | 0.892834917 | 0.362602819 | 0.815250394 |
| AC079160.1 | 1.058643161 | 0.364111307 | 0.815250394 |
| AL358334.2 | 1.007065112 | 0.364111307 | 0.815250394 |
| DLG5-AS1 | 0.625082736 | 0.363323983 | 0.815250394 |
| LINC01050 | 1.183015469 | 0.362602819 | 0.815250394 |
| LINC02605 | 0.886797579 | 0.364440098 | 0.815250394 |
| MSL3P1 | 0.685932993 | 0.364440098 | 0.815250394 |
| SCAT2 | 0.73601592 | 0.363323983 | 0.815250394 |
| TFAP2A-AS1 | 0.825746164 | 0.364440098 | 0.815250394 |
| AF124730.2 | 0.930849922 | 0.365356751 | 0.815780383 |
| AC004241.3 | 0.6915281 | 0.37195893 | 0.826496302 |
| MHENCR | 0.408641458 | 0.371838773 | 0.826496302 |
| PLA2G4E-AS1 | 1.146487739 | 0.377249287 | 0.837450138 |
| AL096828.3 | 0.822657042 | 0.378339186 | 0.83803066 |
| AL136162.1 | 0.985083849 | 0.378339186 | 0.83803066 |
| AC002470.1 | 0.673095128 | 0.382472566 | 0.839580268 |
| AC026782.2 | 1.373895731 | 0.382617815 | 0.839580268 |
| AC093382.1 | 0.871933764 | 0.382617815 | 0.839580268 |
| AC126407.1 | 0.993211893 | 0.382617815 | 0.839580268 |
| AC145098.1 | 0.536233725 | 0.382510458 | 0.839580268 |
| C10orf91 | 0.73877624 | 0.380332341 | 0.839580268 |
| KRT17P2 | 0.861167977 | 0.381011256 | 0.839580268 |
| AC006213.2 | -0.546590964 | 0.387245807 | 0.840359592 |
| AC015802.1 | 0.896787364 | 0.386378557 | 0.840359592 |
| AC129507.1 | -0.617745142 | 0.387245807 | 0.840359592 |
| AC245100.7 | 0.73397595 | 0.386834643 | 0.840359592 |
| AL732314.8 | -0.668328508 | 0.387178683 | 0.840359592 |
| ZNF658B | -0.591575678 | 0.387178683 | 0.840359592 |
| AC025423.1 | 0.836540074 | 0.391913962 | 0.848903189 |
| AC027763.2 | 0.55443174 | 0.392816443 | 0.849273538 |
| AC116025.2 | 0.72484388 | 0.392669705 | 0.849273538 |
| ERICH6-AS1 | 0.639470643 | 0.393361704 | 0.849661281 |
| CCDC162P | 0.590901778 | 0.394803399 | 0.851982799 |
| AL357143.1 | 1.007547362 | 0.396692146 | 0.854470466 |
| RPL6P27 | -0.192882396 | 0.396566776 | 0.854470466 |
| LINC01389 | 0.421409352 | 0.400449147 | 0.860965665 |
| AL391845.2 | 1.380233318 | 0.400903391 | 0.860975725 |
| AC005041.3 | 0.533519609 | 0.402873666 | 0.862186776 |
| AP005436.3 | 0.786273201 | 0.40283756 | 0.862186776 |
| AL139288.1 | 0.690198807 | 0.404177091 | 0.863384733 |
| SLC2A1-AS1 | 0.826674264 | 0.405037265 | 0.864426957 |
| AL353194.1 | 0.403549815 | 0.407460781 | 0.868003609 |
| AL512625.2 | 0.890961406 | 0.407438932 | 0.868003609 |
| MIAT | 0.647127224 | 0.408898868 | 0.870268717 |
| PRRT3-AS1 | 0.38347493 | 0.409347634 | 0.870426013 |
| AC078788.1 | 0.917804289 | 0.413486919 | 0.876818836 |
| LINC00505 | 0.960991834 | 0.413486919 | 0.876818836 |
| LINC02527 | 0.635545558 | 0.413486919 | 0.876818836 |
| AC144652.1 | 0.6751064 | 0.414713563 | 0.877816676 |
| AC011997.1 | 1.039084096 | 0.419881051 | 0.884722141 |
| AC061992.2 | 0.774662128 | 0.419881051 | 0.884722141 |
| AC009495.2 | -0.663021468 | 0.420683524 | 0.884807195 |
| AC007996.1 | 0.457280249 | 0.422833123 | 0.885716946 |
| AC039056.2 | 0.762392615 | 0.424549079 | 0.885716946 |
| AC092803.1 | 0.79783045 | 0.424549079 | 0.885716946 |
| AC106772.1 | 0.552539696 | 0.423828543 | 0.885716946 |
| LINC00239 | 0.499482561 | 0.42681499 | 0.888847001 |
| AC090709.1 | 1.247966398 | 0.430811745 | 0.893963245 |
| LINC02542 | 0.251559398 | 0.43515723 | 0.901369391 |
| AC109322.1 | 0.550985208 | 0.436254763 | 0.902837397 |
| AC018616.1 | 0.827516965 | 0.44154812 | 0.905719731 |
| CLEC12A-AS1 | 0.863056719 | 0.44154812 | 0.905719731 |
| CTD-2574D22.4 | 0.374716008 | 0.441367012 | 0.905719731 |
| AC010761.1 | 0.436830696 | 0.443400139 | 0.906811335 |
| AC069360.1 | 0.621793167 | 0.443264228 | 0.906811335 |
| COA6-AS1 | 0.493586592 | 0.443521835 | 0.906811335 |
| AL354892.2 | 0.333817455 | 0.446718759 | 0.912296357 |
| AC034231.1 | 0.459850161 | 0.44819852 | 0.914514028 |
| LINC01560 | 0.573977976 | 0.449604112 | 0.916576601 |
| AL590617.2 | 0.480480665 | 0.452771128 | 0.921415039 |
| ALG1L13P | 0.764200411 | 0.45444696 | 0.922586509 |
| DDX11-AS1 | 1.020216942 | 0.455770252 | 0.922586509 |
| PRR7-AS1 | 0.646156264 | 0.45635347 | 0.922586509 |
| AC002091.1 | 0.683504154 | 0.459442248 | 0.925665048 |
| AC004130.2 | 0.446760967 | 0.459643325 | 0.925665048 |
| AC115522.1 | 0.500868516 | 0.459502654 | 0.925665048 |
| EXOC3-AS1 | 0.472105171 | 0.459292889 | 0.925665048 |
| LINC01730 | 0.645365799 | 0.462263446 | 0.929693398 |
| AC027031.2 | 0.718129733 | 0.464705582 | 0.931818965 |
| LINC01597 | -0.542634803 | 0.468746835 | 0.938301853 |
| AL021392.1 | 0.945730793 | 0.471037285 | 0.94059829 |
| AL035587.1 | 0.455539174 | 0.471309492 | 0.94059829 |
| HLA-V | 0.810174258 | 0.471037285 | 0.94059829 |
| LINC01414 | 0.806835955 | 0.471514388 | 0.94059829 |
| LINC01249 | 1.006386369 | 0.472273116 | 0.941303156 |
| AC002091.2 | 0.602540802 | 0.473404652 | 0.942749229 |
| AC007406.3 | 1.014611986 | 0.474639192 | 0.943589215 |
| AL021578.1 | 0.56218392 | 0.475075319 | 0.943648325 |
| AC138207.5 | 0.360368024 | 0.480536206 | 0.95124047 |
| AC004812.2 | 0.446579754 | 0.495826207 | 0.973210865 |
| AC002398.1 | 0.435873364 | 0.496888332 | 0.974471881 |
| AP002807.1 | 0.352001227 | 0.498164498 | 0.975529829 |
| RP11-465N4.5 | 0.219700685 | 0.498978235 | 0.976097273 |
| AC098934.1 | 0.422334889 | 0.501326545 | 0.978218687 |
| CDK6-AS1 | 0.936817763 | 0.502993721 | 0.978378305 |
| LINC00114 | 0.75265663 | 0.503515105 | 0.978378305 |
| NORAD | -0.108381853 | 0.501851121 | 0.978378305 |
| AC233992.3 | 0.320880094 | 0.505537176 | 0.978719207 |
| RP11-148O21.2 | -0.451010766 | 0.504963892 | 0.978719207 |
| SNHG11 | 0.279602456 | 0.504982409 | 0.978719207 |
| AC005330.1 | 0.673220061 | 0.512189863 | 0.981230167 |
| AC113143.1 | 0.407318369 | 0.510187867 | 0.981230167 |
| AC133644.2 | 0.93142675 | 0.510493402 | 0.981230167 |
| AC139530.1 | 0.488950434 | 0.512849947 | 0.981230167 |
| AL137802.2 | 0.712086969 | 0.512189863 | 0.981230167 |
| RUNDC3A-AS1 | 0.745188998 | 0.512189863 | 0.981230167 |
| SBF2-AS1 | 0.406487315 | 0.509876257 | 0.981230167 |
| KTN1-AS1 | 0.626903219 | 0.519295766 | 0.989175365 |
| AC004846.1 | 0.729730588 | 0.523334555 | 0.99416753 |
| AC011445.2 | 0.486457922 | 0.526626211 | 0.99416753 |
| AL122125.1 | 0.804469309 | 0.525462154 | 0.99416753 |
| LINC01138 | 0.451457466 | 0.523605916 | 0.99416753 |
| SAMD12-AS1 | 0.677254914 | 0.525462154 | 0.99416753 |
| SUZ12P1 | 0.333862993 | 0.525581843 | 0.99416753 |
| AC008687.2 | 1.010309996 | 0.535070414 | 0.995125244 |
| AC011352.1 | 0.8295817 | 0.537688443 | 0.995125244 |
| AC023825.2 | 0.666866719 | 0.535070414 | 0.995125244 |
| AC092422.1 | -0.549217982 | 0.532010108 | 0.995125244 |
| AC093866.1 | 0.85398356 | 0.537688443 | 0.995125244 |
| AC125807.2 | 0.291003031 | 0.528506829 | 0.995125244 |
| AL356417.2 | 0.831556045 | 0.537688443 | 0.995125244 |
| AP000424.2 | 0.569607273 | 0.537688443 | 0.995125244 |
| AP002336.3 | 0.660266107 | 0.537132804 | 0.995125244 |
| AP002907.1 | 0.502373314 | 0.531993526 | 0.995125244 |
| AP005057.1 | 1.079363055 | 0.537688443 | 0.995125244 |
| LINC01977 | 0.805006787 | 0.535070414 | 0.995125244 |
| LINC02466 | 0.514358332 | 0.537688443 | 0.995125244 |
| MIPEPP3 | 0.36685062 | 0.534627983 | 0.995125244 |
| MLIP-IT1 | 0.718865415 | 0.537132804 | 0.995125244 |
| OR9K1P | 1.042531077 | 0.537688443 | 0.995125244 |
| RALY-AS1 | 0.372995873 | 0.533539372 | 0.995125244 |
| AC091153.3 | 0.800068652 | 0.542214019 | 0.996849527 |
| RNASEH1-AS1 | 0.272005826 | 0.541871314 | 0.996849527 |
| AL157871.2 | 0.485549667 | 0.543179171 | 0.997833889 |
| ABALON | 0.458755851 | 0.559349707 | 1 |
| AC002066.1 | 0.410123291 | 1 | 1 |
| AC002511.2 | -0.414330874 | 0.560318952 | 1 |
| AC002558.3 | 0.179287875 | 0.991482023 | 1 |
| AC003070.1 | 0.212882881 | 0.715875868 | 1 |
| AC004069.1 | 0.594818237 | 0.802087002 | 1 |
| AC004447.2 | 0.011663855 | 1 | 1 |
| AC004471.1 | 0.560852989 | 0.903665 | 1 |
| AC004540.1 | 0.488182652 | 0.777794345 | 1 |
| AC004551.1 | 0.293010725 | 0.846507141 | 1 |
| AC004706.1 | 0.48781077 | 0.728475381 | 1 |
| AC004847.1 | 0.492120344 | 0.838796668 | 1 |
| AC004908.2 | 0.211303131 | 0.924912232 | 1 |
| AC004920.1 | 0.385813481 | 0.746674564 | 1 |
| AC004943.1 | 0.097063663 | 1 | 1 |
| AC004943.2 | 0.421218214 | 0.586283583 | 1 |
| AC004988.1 | 0.726270516 | 0.613153245 | 1 |
| AC004990.1 | 0.284194105 | 0.790716299 | 1 |
| AC005224.3 | -0.079327549 | 0.753310459 | 1 |
| AC005229.4 | 0.258585461 | 0.871409139 | 1 |
| AC005253.1 | -0.076928746 | 0.854781776 | 1 |
| AC005332.4 | 0.203922543 | 0.938818742 | 1 |
| AC005479.1 | 0.518378876 | 0.578635818 | 1 |
| AC005515.1 | 0.882593411 | 0.741761665 | 1 |
| AC005537.2 | 0.69830571 | 0.699389446 | 1 |
| AC005562.1 | -0.01593521 | 0.821376806 | 1 |
| AC005808.1 | 0.051174903 | 1 | 1 |
| AC005954.1 | -0.091107261 | 1 | 1 |
| AC006064.2 | -0.121507238 | 1 | 1 |
| AC006252.1 | 0.511007902 | 0.959054194 | 1 |
| AC006262.1 | 0.329626537 | 1 | 1 |
| AC006449.5 | 0.565605215 | 0.852017995 | 1 |
| AC006460.1 | 0.433588171 | 0.730600653 | 1 |
| AC007032.1 | 0.308195736 | 1 | 1 |
| AC007285.2 | 0.290468404 | 0.826831754 | 1 |
| AC007365.1 | 0.66580862 | 0.752475649 | 1 |
| AC007448.4 | 0.289748991 | 1 | 1 |
| AC007540.2 | 0.417407294 | 1 | 1 |
| AC007663.3 | 0.430679719 | 1 | 1 |
| AC007728.3 | 0.333128497 | 1 | 1 |
| AC007773.1 | 0.459263328 | 0.907564694 | 1 |
| AC007823.1 | 0.280613174 | 1 | 1 |
| AC007938.3 | 0.505582244 | 0.649354715 | 1 |
| AC007952.4 | 0.57830674 | 1 | 1 |
| AC008083.3 | 0.512682316 | 0.730600653 | 1 |
| AC008114.1 | 0.072079001 | 1 | 1 |
| AC008443.1 | 0.06524157 | 1 | 1 |
| AC008543.1 | 0.353183156 | 1 | 1 |
| AC008610.1 | 0.30182573 | 0.637790098 | 1 |
| AC008622.2 | 0.239000824 | 1 | 1 |
| AC008764.8 | 0.217617898 | 1 | 1 |
| AC009093.1 | 0.706913439 | 0.601424313 | 1 |
| AC009097.2 | 0.517484951 | 0.699389446 | 1 |
| AC009226.1 | 0.363743672 | 1 | 1 |
| AC009269.5 | 0.287777053 | 1 | 1 |
| AC009902.3 | 0.483623149 | 0.96170965 | 1 |
| AC009950.1 | 0.12282132 | 0.952888774 | 1 |
| AC010148.1 | -0.107942148 | 1 | 1 |
| AC010247.2 | 0.416143474 | 1 | 1 |
| AC010255.2 | 0.692172989 | 1 | 1 |
| AC010331.1 | 0.677383128 | 0.576869839 | 1 |
| AC010359.2 | 0.770054303 | 0.563103158 | 1 |
| AC010486.3 | 0.671293907 | 1 | 1 |
| AC010491.1 | 0.298568294 | 1 | 1 |
| AC010969.2 | 0.097691823 | 1 | 1 |
| AC011298.1 | 0.814073224 | 0.794586982 | 1 |
| AC011337.1 | 0.62450948 | 0.57039592 | 1 |
| AC011446.1 | 0.295304671 | 0.8887282 | 1 |
| AC011483.1 | 0.221810531 | 0.749899185 | 1 |
| AC012063.1 | 0.170311565 | 1 | 1 |
| AC012158.1 | 0.46906677 | 1 | 1 |
| AC012306.2 | 0.007911048 | 0.98521057 | 1 |
| AC012313.3 | 0.554634713 | 0.752475649 | 1 |
| AC012360.3 | 0.282381636 | 0.83414591 | 1 |
| AC012615.1 | 0.078139353 | 1 | 1 |
| AC013652.1 | 0.585959432 | 0.631734974 | 1 |
| AC015712.2 | 0.0853061 | 1 | 1 |
| AC015712.6 | 0.612890734 | 0.96170965 | 1 |
| AC015802.5 | 0.394736988 | 1 | 1 |
| AC015849.5 | 0.834994287 | 0.741761665 | 1 |
| AC015982.2 | 0.304463112 | 0.832020055 | 1 |
| AC016542.1 | 0.341286398 | 1 | 1 |
| AC016737.1 | 0.684552193 | 0.670220024 | 1 |
| AC016876.3 | 0.281355489 | 0.872324558 | 1 |
| AC016877.3 | 0.671177341 | 0.808267502 | 1 |
| AC017083.1 | 0.415707841 | 0.984092508 | 1 |
| AC018645.3 | 0.051660776 | 0.977796783 | 1 |
| AC018695.3 | 0.231132688 | 1 | 1 |
| AC019186.1 | 0.747248151 | 0.752475649 | 1 |
| AC019205.1 | 0.415281151 | 0.891710642 | 1 |
| AC020558.2 | 0.319794123 | 1 | 1 |
| AC020765.2 | 0.211894921 | 1 | 1 |
| AC020900.1 | 0.579148953 | 1 | 1 |
| AC020934.2 | 0.102126264 | 1 | 1 |
| AC021321.1 | 0.30479526 | 1 | 1 |
| AC021851.1 | 0.46028808 | 0.637565721 | 1 |
| AC022126.1 | 0.490468059 | 0.846507141 | 1 |
| AC022400.2 | 0.249981824 | 1 | 1 |
| AC022509.1 | 0.374640491 | 1 | 1 |
| AC022893.2 | -0.326676185 | 0.769454043 | 1 |
| AC023644.1 | 0.194550085 | 0.988382786 | 1 |
| AC023813.3 | -0.079521019 | 1 | 1 |
| AC023906.5 | 0.210239426 | 0.887684748 | 1 |
| AC023908.3 | 0.074560414 | 1 | 1 |
| AC025031.3 | 0.740258433 | 0.601424313 | 1 |
| AC025171.4 | 0.086361899 | 1 | 1 |
| AC025176.1 | 0.392821782 | 0.621011217 | 1 |
| AC026304.1 | 0.571533082 | 0.826831754 | 1 |
| AC026470.2 | 0.421591634 | 0.655139043 | 1 |
| AC026803.2 | 0.049025774 | 1 | 1 |
| AC027601.2 | 0.139109679 | 1 | 1 |
| AC027627.1 | 0.343173023 | 1 | 1 |
| AC027702.2 | 0.622363457 | 0.559349707 | 1 |
| AC034236.2 | 0.053204448 | 1 | 1 |
| AC036176.3 | 0.263233389 | 0.754014388 | 1 |
| AC046134.2 | 0.273697818 | 0.950956638 | 1 |
| AC046143.2 | 0.225228431 | 0.707171521 | 1 |
| AC053503.1 | 0.265112806 | 1 | 1 |
| AC063948.1 | 0.292420844 | 0.984092508 | 1 |
| AC064836.3 | 0.143784608 | 1 | 1 |
| AC067945.3 | 0.335702046 | 1 | 1 |
| AC068025.1 | 0.697445231 | 0.590125506 | 1 |
| AC068189.1 | 0.126519035 | 1 | 1 |
| AC068228.2 | 0.168779604 | 1 | 1 |
| AC068473.4 | 0.945916208 | 0.883755103 | 1 |
| AC069257.2 | 0.429164277 | 0.612642242 | 1 |
| AC073195.2 | 0.615329558 | 0.650788016 | 1 |
| AC073517.1 | 0.29637802 | 0.95691224 | 1 |
| AC073869.1 | 0.006208718 | 0.947179371 | 1 |
| AC073964.1 | -0.020601673 | 1 | 1 |
| AC083841.2 | 0.880216892 | 0.883755103 | 1 |
| AC083862.2 | 0.25124516 | 1 | 1 |
| AC084117.1 | 0.200660918 | 0.69778435 | 1 |
| AC084346.1 | 0.401363312 | 1 | 1 |
| AC087239.1 | 0.858039616 | 0.578635818 | 1 |
| AC087499.2 | 0.032210065 | 1 | 1 |
| AC087741.2 | 0.469287696 | 0.688165767 | 1 |
| AC087742.1 | 0.352588894 | 1 | 1 |
| AC090282.1 | 0.464833273 | 1 | 1 |
| AC090517.2 | 0.260356083 | 0.875634772 | 1 |
| AC090907.1 | 0.451312094 | 0.866699695 | 1 |
| AC090912.2 | 0.568906959 | 0.618223032 | 1 |
| AC092115.4 | 0.42131271 | 0.685308089 | 1 |
| AC092171.3 | 0.134500091 | 1 | 1 |
| AC092614.1 | 0.523146188 | 0.826831754 | 1 |
| AC092718.6 | -0.26840832 | 1 | 1 |
| AC092807.3 | 0.715733188 | 1 | 1 |
| AC092903.2 | 0.367101555 | 0.9312872 | 1 |
| AC092910.3 | 0.051784952 | 1 | 1 |
| AC093520.1 | 0.250213319 | 1 | 1 |
| AC093635.1 | 0.56838188 | 0.837159161 | 1 |
| AC093677.2 | 0.263827723 | 1 | 1 |
| AC093817.1 | 0.019418605 | 1 | 1 |
| AC096711.1 | 0.962613044 | 0.579421574 | 1 |
| AC097468.1 | -0.649702855 | 0.637734603 | 1 |
| AC098487.1 | 0.487607212 | 0.641954264 | 1 |
| AC099518.2 | 0.164084254 | 0.730600653 | 1 |
| AC099518.4 | 0.139911191 | 1 | 1 |
| AC099522.2 | 0.197029468 | 1 | 1 |
| AC099548.2 | -0.116818966 | 0.736786492 | 1 |
| AC100791.2 | 0.175198927 | 1 | 1 |
| AC100791.3 | 0.529557139 | 1 | 1 |
| AC103706.1 | 0.480462371 | 0.77847024 | 1 |
| AC105429.1 | 0.016880026 | 1 | 1 |
| AC106052.1 | 0.191841088 | 1 | 1 |
| AC106820.3 | 0.53418065 | 0.641363028 | 1 |
| AC107081.2 | 0.239316218 | 1 | 1 |
| AC108134.2 | 0.381194849 | 0.603352437 | 1 |
| AC108463.2 | 0.622650316 | 0.624890729 | 1 |
| AC108463.3 | 0.62606908 | 0.618223032 | 1 |
| AC108673.2 | 0.640689468 | 0.594774508 | 1 |
| AC108751.4 | 0.328614425 | 1 | 1 |
| AC109479.1 | -0.078648564 | 1 | 1 |
| AC112484.1 | 0.536325577 | 0.655139043 | 1 |
| AC112484.3 | -0.166416097 | 0.845298482 | 1 |
| AC112493.1 | 0.764251184 | 0.654770587 | 1 |
| AC114341.1 | 0.500171315 | 0.816506194 | 1 |
| AC114763.1 | 0.123682009 | 1 | 1 |
| AC115837.2 | 0.393497657 | 1 | 1 |
| AC116903.2 | 0.52858037 | 1 | 1 |
| AC116914.1 | -0.081884996 | 1 | 1 |
| AC117490.2 | 0.386555779 | 0.857662541 | 1 |
| AC120114.1 | 0.374443505 | 0.796807606 | 1 |
| AC124016.2 | 0.257989584 | 1 | 1 |
| AC126773.4 | 0.731037758 | 0.600559015 | 1 |
| AC130650.2 | 0.162270319 | 1 | 1 |
| AC131009.1 | 0.472034003 | 0.852017995 | 1 |
| AC131025.1 | 0.455745929 | 1 | 1 |
| AC132008.2 | 0.150129395 | 0.636615776 | 1 |
| AC132192.2 | 0.120584437 | 1 | 1 |
| AC133785.1 | 0.536320522 | 0.991014609 | 1 |
| AC134043.2 | 0.430681068 | 1 | 1 |
| AC135279.4 | 0.443513522 | 1 | 1 |
| AC137932.2 | 0.129704996 | 1 | 1 |
| AC138028.4 | 0.182684281 | 0.8631059 | 1 |
| AC138207.4 | 0.390760688 | 0.573377997 | 1 |
| AC138696.2 | 0.278137817 | 0.660877138 | 1 |
| AC138904.1 | 0.865106708 | 0.677727507 | 1 |
| AC139769.2 | -0.431399902 | 0.626493041 | 1 |
| AC140479.4 | -0.478395906 | 0.665861261 | 1 |
| AC145285.6 | 0.194572169 | 0.867629226 | 1 |
| AC145343.1 | 0.257155919 | 1 | 1 |
| AC145423.2 | 0.05568732 | 1 | 1 |
| AC234772.2 | 0.607033206 | 0.585377054 | 1 |
| AC245100.4 | 0.116414552 | 1 | 1 |
| AC245128.3 | 0.173061875 | 1 | 1 |
| ACAP2-IT1 | 0.284120168 | 0.719117551 | 1 |
| AF127936.5 | 0.547833681 | 0.741761665 | 1 |
| AF274858.1 | 0.462365121 | 0.746750268 | 1 |
| AFG3L1P | -0.013994707 | 0.881568103 | 1 |
| AL008718.3 | 0.202324203 | 1 | 1 |
| AL009178.2 | 0.795838894 | 0.647417218 | 1 |
| AL009179.1 | 0.598270265 | 0.601424313 | 1 |
| AL021707.6 | 0.128297653 | 0.731213903 | 1 |
| AL022100.1 | 0.426014588 | 1 | 1 |
| AL023755.1 | 0.634207358 | 0.846507141 | 1 |
| AL023803.3 | 0.45655274 | 1 | 1 |
| AL024507.3 | 0.555831465 | 0.730600653 | 1 |
| AL033527.3 | 0.579772914 | 0.775510622 | 1 |
| AL035588.1 | -0.345430549 | 0.778844801 | 1 |
| AL078644.1 | 0.058744478 | 1 | 1 |
| AL078644.2 | 0.445652287 | 0.931081105 | 1 |
| AL109804.1 | 0.38133703 | 1 | 1 |
| AL110115.2 | 0.409715757 | 0.852017995 | 1 |
| AL117332.1 | 0.550831119 | 0.691205704 | 1 |
| AL118556.2 | 0.191013635 | 1 | 1 |
| AL121603.2 | -0.063374868 | 0.855003895 | 1 |
| AL121658.1 | 0.132080484 | 1 | 1 |
| AL121832.3 | 0.49169015 | 0.670220024 | 1 |
| AL121852.1 | 0.107405694 | 1 | 1 |
| AL132780.2 | 0.129634827 | 1 | 1 |
| AL133230.2 | 0.371101109 | 1 | 1 |
| AL133325.3 | 0.204503098 | 1 | 1 |
| AL133338.1 | 0.343344671 | 0.655532024 | 1 |
| AL137782.1 | 0.356484813 | 0.796807606 | 1 |
| AL138759.1 | 0.593305742 | 0.810424879 | 1 |
| AL138962.1 | 0.655870406 | 1 | 1 |
| AL138976.2 | 0.467602462 | 0.753588009 | 1 |
| AL139089.1 | 0.366422285 | 0.800742328 | 1 |
| AL139289.1 | 0.333827073 | 0.726110586 | 1 |
| AL157394.1 | 0.273737237 | 0.907564694 | 1 |
| AL157400.4 | 0.415649281 | 0.810424879 | 1 |
| AL157838.1 | 0.284039463 | 0.844320312 | 1 |
| AL157904.1 | 0.818686844 | 0.578635818 | 1 |
| AL157932.1 | 0.455682196 | 0.655532024 | 1 |
| AL160408.5 | 0.983336403 | 0.810424879 | 1 |
| AL161421.1 | 0.26427223 | 0.738689597 | 1 |
| AL161452.1 | 0.151970311 | 0.943460122 | 1 |
| AL161756.1 | -0.048355969 | 1 | 1 |
| AL163051.1 | 0.095869522 | 0.952888774 | 1 |
| AL353593.2 | 0.624697823 | 0.808267502 | 1 |
| AL353719.1 | 0.331690913 | 1 | 1 |
| AL353768.1 | 0.280468034 | 1 | 1 |
| AL353796.1 | 0.064668274 | 1 | 1 |
| AL354793.1 | 0.40939879 | 0.96170965 | 1 |
| AL354813.1 | 0.464298782 | 1 | 1 |
| AL355075.4 | 0.036656913 | 1 | 1 |
| AL355303.1 | 0.395104136 | 1 | 1 |
| AL355304.1 | 0.248668311 | 1 | 1 |
| AL355312.2 | 0.124882659 | 1 | 1 |
| AL355388.1 | 0.109233906 | 1 | 1 |
| AL355607.2 | 0.805073406 | 0.587541077 | 1 |
| AL355803.1 | 0.727136912 | 0.599760123 | 1 |
| AL356299.3 | 0.266560436 | 0.9312872 | 1 |
| AL356752.1 | 0.279710482 | 1 | 1 |
| AL358334.3 | 0.571020473 | 0.725591696 | 1 |
| AL358472.3 | 0.101510844 | 1 | 1 |
| AL359198.1 | 0.326796044 | 1 | 1 |
| AL359643.3 | 0.233892422 | 0.780566095 | 1 |
| AL359922.2 | 0.516645975 | 0.931081105 | 1 |
| AL365295.1 | 0.299635436 | 1 | 1 |
| AL390728.4 | 0.072257608 | 0.819335804 | 1 |
| AL390879.1 | 0.375646352 | 0.778610028 | 1 |
| AL390961.2 | -0.020090753 | 1 | 1 |
| AL391069.2 | 0.459166031 | 0.838796668 | 1 |
| AL391152.1 | 0.805842796 | 0.775510622 | 1 |
| AL391422.4 | 0.109244384 | 1 | 1 |
| AL391825.1 | 0.18801208 | 1 | 1 |
| AL392172.1 | 0.186475709 | 0.709527164 | 1 |
| AL450992.2 | 0.675373928 | 1 | 1 |
| AL451050.2 | 0.537078204 | 0.794548637 | 1 |
| AL451070.1 | 0.321431147 | 1 | 1 |
| AL451085.3 | 0.17203082 | 0.959576178 | 1 |
| AL512408.1 | 0.323144676 | 1 | 1 |
| AL590708.1 | 0.785926565 | 0.808267502 | 1 |
| AL591806.1 | 0.455893557 | 0.991014609 | 1 |
| AL592435.1 | 0.137245207 | 1 | 1 |
| AL596094.1 | 0.439874641 | 0.673020092 | 1 |
| AL603832.1 | -0.027882419 | 1 | 1 |
| AL606534.1 | 0.704637925 | 0.578635818 | 1 |
| AL645608.8 | 0.770783959 | 0.647417218 | 1 |
| AL807757.2 | 0.256466511 | 0.949076677 | 1 |
| ANP32BP1 | 0.035529927 | 1 | 1 |
| AP000424.1 | 0.895109438 | 0.741761665 | 1 |
| AP000553.2 | 0.752913731 | 0.563103158 | 1 |
| AP000619.1 | -0.330933217 | 1 | 1 |
| AP000763.3 | 0.421318506 | 1 | 1 |
| AP000829.1 | 0.882153481 | 0.883755103 | 1 |
| AP001160.3 | 0.200039218 | 0.897965344 | 1 |
| AP001330.4 | 0.164550473 | 0.936607731 | 1 |
| AP001453.4 | 0.327456863 | 0.84786299 | 1 |
| AP001610.2 | 0.798933778 | 0.580597683 | 1 |
| AP001775.2 | 0.429675987 | 1 | 1 |
| AP001793.1 | 0.168023382 | 1 | 1 |
| AP002336.1 | 0.737340455 | 0.600559015 | 1 |
| AP002954.1 | -0.043382329 | 1 | 1 |
| AP003071.2 | -0.287931476 | 0.589696259 | 1 |
| AP003696.1 | 0.643305055 | 0.991014609 | 1 |
| AP005019.1 | 0.816558744 | 0.648339272 | 1 |
| AP005136.3 | 0.474010225 | 0.650788016 | 1 |
| AP005329.1 | 0.312160823 | 1 | 1 |
| ARHGAP5-AS1 | 0.268335112 | 0.759676143 | 1 |
| ARHGEF35-AS1 | 0.826416497 | 0.601424313 | 1 |
| ARRDC1-AS1 | -0.008821908 | 0.941271175 | 1 |
| ASH1L-AS1 | 0.197436146 | 0.793395243 | 1 |
| ATP1B3-AS1 | 0.144012042 | 0.951292696 | 1 |
| ATP2C2-AS1 | 0.440032652 | 0.673020092 | 1 |
| BHLHE40-AS1 | 0.209261534 | 1 | 1 |
| BMS1P17 | 0.69724707 | 0.613153245 | 1 |
| BTN2A3P | 0.294271592 | 0.655532024 | 1 |
| BX322234.1 | 0.291749862 | 0.677764273 | 1 |
| C1RL-AS1 | 0.301948938 | 0.701283982 | 1 |
| C2-AS1 | 0.215955597 | 0.922160762 | 1 |
| CAMTA1-DT | 0.633582921 | 0.603352437 | 1 |
| CAPN10-DT | 0.046007238 | 1 | 1 |
| CASC15 | 0.890581509 | 0.561126382 | 1 |
| CCT6P1 | -0.019228807 | 0.812078174 | 1 |
| CEP250-AS1 | -0.183892742 | 0.821376806 | 1 |
| CEP83-DT | 0.417352078 | 0.96170965 | 1 |
| CHROMR | 0.308238907 | 0.56841976 | 1 |
| CIRBP-AS1 | 0.20351259 | 0.900312537 | 1 |
| CLCA3P | 0.702169511 | 0.546491408 | 1 |
| CPNE8-AS1 | 0.798911427 | 0.594774508 | 1 |
| CRYBB2P1 | 0.026343184 | 0.945417967 | 1 |
| CYMP | -0.020950649 | 0.942276248 | 1 |
| CYMP-AS1 | -0.394797357 | 0.804457956 | 1 |
| DDX39B-AS1 | -0.306980158 | 0.778844801 | 1 |
| DGUOK-AS1 | 0.263113939 | 0.577442096 | 1 |
| DLEU1 | 0.294512895 | 0.83985939 | 1 |
| DLX6-AS1 | 1.095603656 | 0.647417218 | 1 |
| DNAH17-AS1 | 0.539023707 | 0.883755103 | 1 |
| DNAJC3-DT | 0.253405896 | 0.868948883 | 1 |
| DSCR9 | 0.448500731 | 0.969748238 | 1 |
| DSG1-AS1 | -0.292874721 | 0.608336532 | 1 |
| EGOT | 0.645822537 | 0.846507141 | 1 |
| EIPR1-IT1 | 0.295549504 | 1 | 1 |
| ELF3-AS1 | 0.146640959 | 0.58500776 | 1 |
| EML4-AS1 | 0.176415722 | 1 | 1 |
| EP400P1 | 0.04883241 | 1 | 1 |
| FAM111A-DT | 0.1913049 | 0.910476583 | 1 |
| FAM157B | 0.283618319 | 1 | 1 |
| FAM157C | 0.285441038 | 1 | 1 |
| FAM21EP | 0.086218643 | 1 | 1 |
| FAM86C2P | 0.295700148 | 0.640674637 | 1 |
| FMR1-IT1 | 0.234731465 | 1 | 1 |
| FRGCA | 0.60531655 | 1 | 1 |
| FRMD6-AS1 | 0.432817456 | 1 | 1 |
| GAPLINC | 0.30124165 | 0.802087002 | 1 |
| GBAP1 | 0.396758176 | 0.893769449 | 1 |
| HLA-DQB1-AS1 | -0.148004741 | 1 | 1 |
| HOTTIP | -0.012955049 | 1 | 1 |
| HOXA11-AS | 0.246810942 | 0.74320584 | 1 |
| HOXA-AS3 | 0.103285886 | 1 | 1 |
| HSP90AA4P | 0.217331061 | 1 | 1 |
| HSP90AA6P | 0.337986175 | 1 | 1 |
| HSP90AB2P | 0.366595103 | 0.83414591 | 1 |
| HYI-AS1 | 0.114894516 | 1 | 1 |
| IFNG-AS1 | -0.470717542 | 0.685363645 | 1 |
| IL10RB-DT | 0.11302508 | 1 | 1 |
| ILF3-DT | 0.187509339 | 0.599028527 | 1 |
| IPO9-AS1 | 0.085809013 | 1 | 1 |
| ITGB2-AS1 | 0.593147182 | 0.802087002 | 1 |
| JARID2-AS1 | 0.376235269 | 0.837159161 | 1 |
| KDM4A-AS1 | 0.383497392 | 0.624311903 | 1 |
| KIF26B-AS1 | 0.024894619 | 0.909350244 | 1 |
| LINC00240 | 0.533230173 | 1 | 1 |
| LINC00243 | 0.171485352 | 1 | 1 |
| LINC00337 | 0.745698014 | 0.647417218 | 1 |
| LINC00525 | 0.486156419 | 0.677727507 | 1 |
| LINC00539 | 0.160897348 | 1 | 1 |
| LINC00592 | 0.639297884 | 0.896874539 | 1 |
| LINC00630 | 0.120587355 | 1 | 1 |
| LINC00680 | 0.271156289 | 0.707566116 | 1 |
| LINC00853 | 0.264878028 | 0.77847024 | 1 |
| LINC00858 | 0.153899187 | 0.685308089 | 1 |
| LINC00866 | 0.460507322 | 1 | 1 |
| LINC00887 | 0.514447521 | 0.877633627 | 1 |
| LINC00898 | 0.70696318 | 0.654770587 | 1 |
| LINC01094 | 0.488473246 | 0.57039592 | 1 |
| LINC01096 | 0.832854301 | 0.664258298 | 1 |
| LINC01126 | 0.230584769 | 1 | 1 |
| LINC01213 | 0.362342294 | 0.922160762 | 1 |
| LINC01226 | 0.263684916 | 1 | 1 |
| LINC01270 | 0.772099693 | 0.649038999 | 1 |
| LINC01285 | 0.183984893 | 1 | 1 |
| LINC01300 | -0.057044018 | 1 | 1 |
| LINC01311 | 0.569723708 | 0.580597683 | 1 |
| LINC01397 | -0.212923552 | 1 | 1 |
| LINC01415 | 0.851643209 | 0.709170925 | 1 |
| LINC01424 | 0.354765558 | 0.96170965 | 1 |
| LINC01521 | 0.322864975 | 0.77847024 | 1 |
| LINC01572 | 0.359774744 | 1 | 1 |
| LINC01686 | 0.60250131 | 0.677727507 | 1 |
| LINC01857 | 0.26476061 | 0.881935304 | 1 |
| LINC01873 | 0.686126769 | 1 | 1 |
| LINC01905 | 1.071361553 | 0.677727507 | 1 |
| LINC01952 | 0.474430419 | 1 | 1 |
| LINC02043 | 0.567938275 | 0.883755103 | 1 |
| LINC02166 | 0.249973623 | 0.894549473 | 1 |
| LINC02320 | 0.260343392 | 1 | 1 |
| LINC02328 | 0.740906977 | 0.725591696 | 1 |
| LINC02345 | 0.559045336 | 0.618223032 | 1 |
| LINC02528 | 0.512429414 | 0.699213714 | 1 |
| LINC02574 | 0.769773568 | 0.613153245 | 1 |
| LINC02585 | 0.139905408 | 0.853021223 | 1 |
| LINC02611 | -0.092788344 | 1 | 1 |
| LINC02761 | 0.708640339 | 0.802087002 | 1 |
| LINC02806 | 0.692820313 | 0.613153245 | 1 |
| LINC02863 | 0.275578892 | 1 | 1 |
| LNCOC1 | 0.704344989 | 1 | 1 |
| LNCOG | 0.732052068 | 1 | 1 |
| LRRC37A7P | -0.36237087 | 0.637734603 | 1 |
| LRRC37BP1 | 0.030511299 | 1 | 1 |
| LUARIS | 0.4203986 | 1 | 1 |
| MAST4-AS1 | 0.369402793 | 0.950956638 | 1 |
| MATN1-AS1 | 0.096015734 | 0.922589537 | 1 |
| ZSCAN12P1 | 0.152237766 | 0.858011341 | 1 |
