# Supplementary material for: Identification of hub necroptosis-related lncRNAs for prognosis prediction of esophageal carcinoma
Source: Aging (Albany NY). 2023 Jun 1;15(11):4794–819. doi: 10.18632/aging.204763 (PMC10292891; doi:10.18632/aging.204763)
Supplement: Supplementary Tables 6 and 7 [file aging-15-204763-s006.pdf]

## Supplementary Table 6. Evaluation of 6-NRL signature based on time-dependent ROC and C-index.

| Models | 1 years | 3 years | 5 years | *p-value* | C-index[95%(CI)] | HR[95%(low, high)] | *p-value* | PMID |
|---|---|---|---|---|---|---|---|---|
| NRLs signature | 0.784 | 0.827 | 0.764 | <0.001 | 0.759[0.721, 0.797] | 1.322(1.158-1.510) | <0.001 | - |
| Song-JH signature | 0.593 | 0.729 | 0.683 | 0.015 | 0.615[0.584, 0.646] | 2.212(1.437-3.404) | <0.001 | 34291083 |
| Ye-JC signature | 0.611 | 0.704 | 0.709 | 0.006 | 0.599[0.569, 0.629] | 2.251(1.353-3.747) | 0.002 | 34764976 |
| Zhao-MN signature | 0.561 | 0.701 | 0.736 | 0.030 | 0.574[0.545, 0.603] | 1.909(1.018-3.581) | 0.044 | 35371305 |
| Yao-JN signature | 0.591 | 0.700 | 0.659 | 0.022 | 0.584[0.555, 0.613] | 2.018(1.201-3.390) | 0.008 | 34926275 |
| Hu-ZN signature | 0.573 | 0.700 | 0.780 | 0.028 | 0.552[0.524, 0.580] | 1.910(0.818-4.460) | 0.135 | 34840568 |
| Yi-LL signature | 0.747 | 0.688 | 0.627 | 0.002 | 0.659[0.626, 0.692] | 1.651(1.298-2.101) | <0.001 | 32810627 |
| Zhao-FC signature | 0.610 | 0.688 | 0.666 | 0.286 | 0.573[0.544, 0.602] | 2.430(0.750-7.872) | 0.139 | 35309940 |
| Wang-L signature | 0.588 | 0.678 | 0.740 | 0.023 | 0.624[0.593, 0.655] | 1.640(1.253-2.146) | <0.001 | 34257547 |
| Zhao-FC signature | 0.555 | 0.676 | 0.691 | 0.009 | 0.542[0.515, 0.569] | 2.238(0.986-5.077) | 0.054 | 35646892 |
| Xiong-K signature | 0.645 | 0.670 | 0.611 | 0.011 | 0.611[0.580, 0.642] | 2.632(1.533-4.517) | <0.001 | 35497331 |
| Zhuang-WT signature | 0.652 | 0.669 | 0.579 | 0.003 | 0.619[0.588, 0.650] | 1.924(1.058-3.500) | 0.032 | 34976055 |
| Liu-W signature | 0.584 | 0.663 | 0.688 | 0.046 | 0.546[0.519, 0.573] | 2.534(0.912-7.039) | 0.075 | 36180848 |
| Shi-XB signature | 0.603 | 0.661 | 0.574 | 0.133 | 0.604[0.574, 0.634] | 1.386(1.130-1.700) | 0.002 | 35121801 |
| Lian-L signature | 0.619 | 0.644 | 0.637 | 0.020 | 0.602[0.572, 0.632] | 1.973(1.305-2.982) | 0.001 | 35557566 |
| Zhang-CQ signature | 0.583 | 0.636 | 0.658 | 0.029 | 0.577[0.548, 0.606] | 1.952(1.009-3.774) | 0.047 | 32883946 |
| He-WW signature | 0.650 | 0.634 | 0.640 | 0.254 | 0.599[0.569, 0.629] | 2.367(1.389-4.036) | 0.002 | 31423201 |
| Xie-JH signature | 0.528 | 0.628 | 0.741 | 0.241 | 0.531[0.504, 0.558] | 1.512(1.123-2.036) | 0.006 | 35282133 |
| Xiao-WY signature | 0.522 | 0.625 | 0.666 | 0.141 | 0.545[0.518, 0.572] | 2.618(1.104-6.206) | 0.029 | 35789548 |
| Lan-T signature | 0.583 | 0.615 | 0.694 | 0.444 | 0.565[0.537, 0.593] | 2.456(1.180-5.112) | 0.016 | 31966072 |
| Chen-FN signature | 0.558 | 0.613 | 0.703 | 0.020 | 0.566[0.538, 0.594] | 1.939(1.247-3.013) | 0.003 | 35611939 |
| Zheng-ZJ signature | 0.526 | 0.611 | 0.467 | 0.193 | 0.552[0.524, 0.580] | 1.456(1.115-1.902) | 0.006 | 35432441 |
| Song-KW signature | 0.566 | 0.606 | 0.730 | 0.048 | 0.560[0.532, 0.588] | 2.038(1.172-3.544) | 0.012 | 36072903 |
| Zhang-CQ signature | 0.581 | 0.599 | 0.602 | 0.203 | 0.558[0.530, 0.586] | 2.753(0.776-9.763) | 0.117 | 32898328 |
| Sun-K signature | 0.559 | 0.599 | 0.534 | 0.632 | 0.555[0.527, 0.583] | 2.407(0.874-6.632) | 0.089 | 35840882 |
| He-ZK signature | 0.598 | 0.594 | 0.693 | 0.121 | 0.596[0.566, 0.626] | 2.523(1.126-5.650) | 0.025 | 35574385 |
| Cui-HY signature | 0.531 | 0.593 | 0.562 | 0.218 | 0.524[0.498, 0.550] | 2.801(0.353-22.198) | 0.329 | 34336650 |
| Zhao-FC signature | 0.684 | 0.592 | 0.609 | 0.160 | 0.616[0.585, 0.647] | 2.486(1.469-4.205) | <0.001 | 35912250 |
| Liu-Y signature | 0.608 | 0.592 | 0.522 | 0.120 | 0.596[0.566, 0.626] | 2.499(1.449-4.310) | <0.001 | 32133283 |
| Zhang-JF signature | 0.606 | 0.589 | 0.535 | 0.223 | 0.587[0.558, 0.616] | 1.947(1.398-2.711) | <0.001 | 35578166 |
| Wu-D signature | 0.682 | 0.583 | 0.579 | 0.113 | 0.604[0.574, 0.634] | 2.242(1.100-4.567) | 0.026 | 34170806 |
| Peng-L signature | 0.524 | 0.577 | 0.619 | 0.892 | 0.510[0.485, 0.536] | 2.605(0.436-15.564) | 0.294 | 31815134 |
| Feng signature | 0.608 | 0.568 | 0.598 | 0.630 | 0.577[0.548, 0.606] | 1.506(1.199-1.892) | <0.001 | 34404882 |
| Zhao-MN signature | 0.573 | 0.568 | 0.571 | 0.313 | 0.574[0.545, 0.603] | 2.435(0.826-7.174) | 0.106 | 35899307 |
| Xu-T signature | 0.504 | 0.561 | 0.746 | 0.447 | 0.501[0.476, 0.526] | 2.359(0.524-10.628) | 0.264 | 35116551 |
| Zhu-T signature | 0.524 | 0.558 | 0.480 | 0.336 | 0.542[0.515, 0.569] | 2.468(0.839-7.263) | 0.101 | 33748133 |
| Pang-JJ signature | 0.671 | 0.554 | 0.730 | 0.049 | 0.629[0.598, 0.660] | 2.602(1.168-5.800) | 0.002 | 34659345 |
| Du-HL signature | 0.540 | 0.552 | 0.529 | 0.213 | 0.542[0.515, 0.569] | 2.264(0.713-7.192) | 0.166 | 33708944 |
| Pu-Y signature | 0.577 | 0.548 | 0.497 | 0.221 | 0.573[0.544, 0.602] | 2.200(0.673-7.190) | 0.192 | 35284129 |
| Chen-YH signature | 0.586 | 0.545 | 0.535 | 0.238 | 0.550[0.523, 0.578] | 2.199(1.024-4.721) | 0.043 | 33960364 |
| Zhang-HP signature | 0.622 | 0.537 | 0.561 | 0.485 | 0.579[0.550, 0.608] | 1.782(1.111-2.859) | 0.017 | 34234806 |
| Chen-FF signature | 0.511 | 0.528 | 0.492 | 0.639 | 0.504[0.479, 0.529] | 2.442(0.362-16.458) | 0.359 | 35928921 |
| Meng-J signature | 0.620 | 0.524 | 0.469 | 0.179 | 0.594[0.564, 0.624] | 2.782(1.202-6.440) | 0.017 | 29160958 |
| Zhang-WG signature | 0.473 | 0.523 | 0.691 | 0.731 | 0.468[0.445, 0.491] | 2.514(0.378-16.734) | 0.341 | 36071753 |
| Tan-LL signature | 0.546 | 0.489 | 0.465 | 0.494 | 0.560[0.532, 0.588] | 2.135(0.935-4.876) | 0.072 | 34814273 |
| Lu-T signature | 0.523 | 0.464 | 0.348 | 0.514 | 0.595[0.565, 0.625] | 2.193(0.591-8.144) | 0.241 | 33981829 |
| Gao-JY signature | 0.534 | 0.455 | 0.360 | 0.937 | 0.536[0.509, 0.563] | 2.349(0.243-22.697) | 0.460 | 33718151 |
| Zhang-CQ signature | 0.614 | 0.451 | 0.424 | 0.304 | 0.576[0.547, 0.605] | 2.335(0.947-5.754) | 0.065 | 33392181 |
| Zhang-CQ signature | 0.544 | 0.408 | 0.302 | 0.695 | 0.547[0.520, 0.574] | 3.217(0.038-269.472) | 0.605 | 35224157 |

**Supplementary Table 7. Top 20 categories with their representative enriched terms (one per category).**

| GO | Category | Description | Count | % | Log10(P) | Log10(q) |
|---|---|---|---|---|---|---|
| hsa04217 | KEGG Pathway | Necroptosis | 27 | 100 | -62.53 | -58.18 |
| hsa05164 | KEGG Pathway | Influenza A | 12 | 44.44 | -19.92 | -15.88 |
| WP4630 | WikiPathways | Measles virus infection | 8 | 29.63 | -12.55 | -8.89 |
| R-HSA-9645723 | Reactome Gene Sets | Diseases of programmed cell death | 7 | 25.93 | -11.37 | -7.98 |
| R-HSA-5218859 | Reactome Gene Sets | Regulated Necrosis | 6 | 22.22 | -10.86 | -7.52 |
| hsa04621 | KEGG Pathway | NOD-like receptor signaling pathway | 7 | 25.93 | -9.62 | -6.61 |
| GO:0009615 | GO Biological Processes | response to virus | 8 | 29.63 | -9.13 | -6.25 |
| GO:0070663 | GO Biological Processes | regulation of leukocyte proliferation | 7 | 25.93 | -8.51 | -5.76 |
| GO:0000302 | GO Biological Processes | response to reactive oxygen species | 6 | 22.22 | -8.08 | -5.41 |
| GO:0043124 | GO Biological Processes | negative regulation of I-kappaB kinase/NF-kappaB signaling | 4 | 14.81 | -6.91 | -4.49 |
| R-HSA-1280215 | Reactome Gene Sets | Cytokine Signaling in Immune system | 8 | 29.63 | -6.78 | -4.38 |
| GO:0002260 | GO Biological Processes | lymphocyte homeostasis | 4 | 14.81 | -6.48 | -4.16 |
| GO:0080135 | GO Biological Processes | regulation of cellular response to stress | 7 | 25.93 | -5.58 | -3.52 |
| GO:0038034 | GO Biological Processes | signal transduction in absence of ligand | 3 | 11.11 | -5.47 | -3.44 |
| WP5083 | WikiPathways | Neuroinflammation and glutamatergic signaling | 4 | 14.81 | -5.14 | -3.17 |
| GO:0001933 | GO Biological Processes | negative regulation of protein phosphorylation | 5 | 18.52 | -4.93 | -2.99 |
| GO:0009612 | GO Biological Processes | response to mechanical stimulus | 4 | 14.81 | -4.41 | -2.54 |
| WP2037 | WikiPathways | Prolactin signaling pathway | 3 | 11.11 | -4.37 | -2.51 |
| GO:1901216 | GO Biological Processes | positive regulation of neuron death | 3 | 11.11 | -4.12 | -2.29 |
| GO:0071496 | GO Biological Processes | cellular response to external stimulus | 4 | 14.81 | -3.76 | -1.97 |
